# A systematic scoping review on group non-written reflections in medical education

**DOI:** 10.1186/s12909-024-06117-3

**Published:** 2024-10-10

**Authors:** Neha Burla, Rui Song Ryan Ong, Ryan Choon Hoe Chee, Ruth Si Man Wong, Shao Yun Neo, Nur Amira Binte Abdul Hamid, Crystal Lim, Eng Koon Ong, Nagavalli Somasundaram, Lalit Kumar Radha Krishna

**Affiliations:** 1 Yong Loo Lin School of Medicine, National University of Singapore, NUHS Tower Block, 1E Kent Ridge Road, Level 11, Singapore, 119228 Singapore; 2 Division of Supportive and Palliative Care, National Cancer Centre Singapore, 30 Hospital Boulevard, Singapore, 168583 Singapore; 3 Division of Cancer Education, National Cancer Centre Singapore, 30 Hospital Boulevard, Singapore, 168583 Singapore; 4 Medical Social Services, Singapore General Hospital, Outram Road, Singapore, 169608 Singapore; 5 Duke-NUS Medical School, National University of Singapore, 8 College Rd, Singapore, 169857 Singapore; 6 Assisi Hospice, 832 Thomson Road, Singapore, 574627 Singapore; 7 Division of Medical Oncology, National Cancer Centre Singapore, 30 Hospital Boulevard, Singapore, 168583 Singapore; 8 Palliative Care Institute Liverpool, Academic Palliative & End of Life Care Centre, University of Liverpool, 200 London Rd, Liverpool, L3 9TA UK; 9 Centre for Biomedical Ethics, National University of Singapore, Blk MD11, 10 Medical Drive, #02-03, Singapore, 117597 Singapore; 10 PalC, The Palliative Care Centre for Excellence in Research and Education, PalC C/O Dover Park Hospice, 10 Jalan Tan Tock Seng, Singapore, 308436 Singapore; 11 Health Data Science, University of Liverpool, Whelan Building The Quadrangle, Brownlow Hill, Liverpool, L69 3GB UK

**Keywords:** Medicine, Physicians, Medical students, Professional identity formation, Reflection, Medical education, Group reflection, Non-written reflection

## Abstract

**Background:**

Medical education is tasked with shaping how medical students and physicians think, feel and act as professionals, or their Professional Identity Formation (PIF). This process has traditionally rested upon imparting knowledge; integrating sociocultural, professional and organizational expectations and codes of conduct; inculcating program and practice beliefs, values and principles (belief systems); and imbuing shared identities – quintessential elements that, together, comprise the socialization process. Key to supporting this socialization process is reflective practice. However, regnant approaches to mobilizing reflective cycles are faced with resource, personnel and time constraints, hindering efforts to nurture PIF. Group non-written reflections (GNWR) – broadly defined as facilitator-led discussions of shared reflective experiences within groups of learners – may prove to be an effective compromise. To address diverse approaches and a lack of effective understanding, we propose a systematic scoping review (SSR) to map the current use of GNWR in medical training and its role in shaping PIF.

**Methods:**

Guided by the Systematic Evidence-Based Approach (SEBA)’s constructivist ontological and relativist epistemological position, this SSR in SEBA searched for articles on GNWR published in PubMed, Embase, Psychinfo, CINAHL, ERIC, ASSIA, SCOPUS, Google Scholar, Open Grey, GreyLit and ProQuest databases. The data found was concurrently analyzed using thematic and direct content analysis. Complementary themes and categories identified were combined, creating the domains that framed the discussion.

**Results:**

Of the 8560 abstracts and 336 full-text articles reviewed, 98 articles were included. The four domains identified were: (1) Indications of use and their value; (2) Structure and how they can be used; (3) Models of reflective practice in GNWR; and (4) Features of communities of practice and the socialisation process.

**Conclusion:**

This SSR in SEBA concludes that GNWR does impact PIF when effectively structured and supported. The Krishna-Pisupati Model for PIF platforms a model that explains GNWR’s effects of PIF and advances fourteen recommendations to maximize GNWR use.

**Supplementary Information:**

The online version contains supplementary material available at 10.1186/s12909-024-06117-3.

## Introduction

Medical education is tasked with shaping how medical students and physicians think, feel and act as professionals, a phenomenon described as Professional Identity Formation (PIF). This process traditionally revolves around imparting key knowledge; integrating sociocultural, professional and organizational expectations and codes of conduct; inculcating program and practice beliefs, values and principles (belief systems); and imbuing shared identities. Cumulatively, these processes scaffold the socialization process that facilitates a learner’s transition from layperson to medical professional.

Key to supporting this socialization process are the program boundaries, structures, codes of conduct and support systems that liken it to a Community of Practice (CoP). It is within this *“persistent, sustaining social network of individuals who share and develop an overlapping knowledge base, set of beliefs, values and history and* *experiences* *focused on a common practice and/or enterprise”* [1] that a mix of role modelling; large group teaching and personalized tutoring; supervised immersion into the clinical field; supervised nurturing of desired competencies; and personalized remediation occurs to shape PIF. Supporting meaning-making exercises, shifting belief systems and identities, as well as a developing sense of belonging and deeper associations, are guided reflective cycles and their accompanying supervised debriefs and personalised reviews. Defined as the metacognitive process of stepping back, reviewing and recognizing how thoughts, feelings, emotions and experiences shape a clinician’s decision-making, clinical reasoning, sense-making and professionalism, reflection is deemed a pivotal constituent of professionalism. Critical reflections on *“disorientating dilemmas”* [2] or threats to professionalism can lead to improvements in future behaviors, attitudes and thinking [2–5]. Unsurprisingly, reflective practice is thus seen to play a critical role in shaping PIF [2, 6, 7].

Yet, facilitating reflective cycles is resource-heavy [8–11]. Hampered by a lack of time, opportunity, structure and trained mentors or supervisors, reflective practice is often compromised, jeopardizing efforts to shape PIF. Recent studies, however, may offer a solution in the form of group non-written reflections (henceforth GNWR). Loosely defined as a facilitator-led discussion of shared reflective experiences in a group of learners, GNWR is less time-consuming and offers a less resource-intensive option to discuss, deconstruct and enrich shared experiences. Its efficacy as a form of reflection, however, remains unclear, alongside other questions that also persist. Whilst it is posited that a multiprofessional team participating in GNWR offers frank discourse on stereotypes, social exclusion and marginalization; differentiates *“social location and subject position in wider socio-economic structures”* [12]; unmasks gender, ethnicity, age, inequality, political issues and multidirectional power relations within the hierarchical medical setting [13]; and proffers multidimensional perspectives [13] and individual views on a shared experience beyond what an individual’s skills and ‘technical rationalism’ permit [12, 14–19], its impact on an individual’s meaning-making remains to be proven. This is concerning, in light of ineffective communications, breaches in professional relationships, unprofessional conduct and clinician burnout that have negatively affected PIF [20, 21].

Nonetheless, Feudtner and Christakis [22], in their discussion of the ethical dilemmas faced by clinical clerks, note that group reflections are indeed successful in unearthing the encouraging and disheartening facets of their clinical experiences [23]. This allows some unfavorable effects to be allayed [23]. Key, however, is the establishment of a safe environment for reflection and discussion in GNWR [23].

Yet, a scarcity of data to suggest a direct association between GNWR and PIF persists. This gap presents yet more reason for evaluation of this approach, as does the use of varied platforms, such as drawing [24] and comic-making [15–18, 25]. Thus, to shape our understanding and guide the design, assessment and oversight of GNWR in medical schools and postgraduate medical training, we propose a systematic scoping review (SSR) to map *“What is known of GNWR in medical education?”.*

## Methodology

### Theoretical framework

**Fig. 1:**
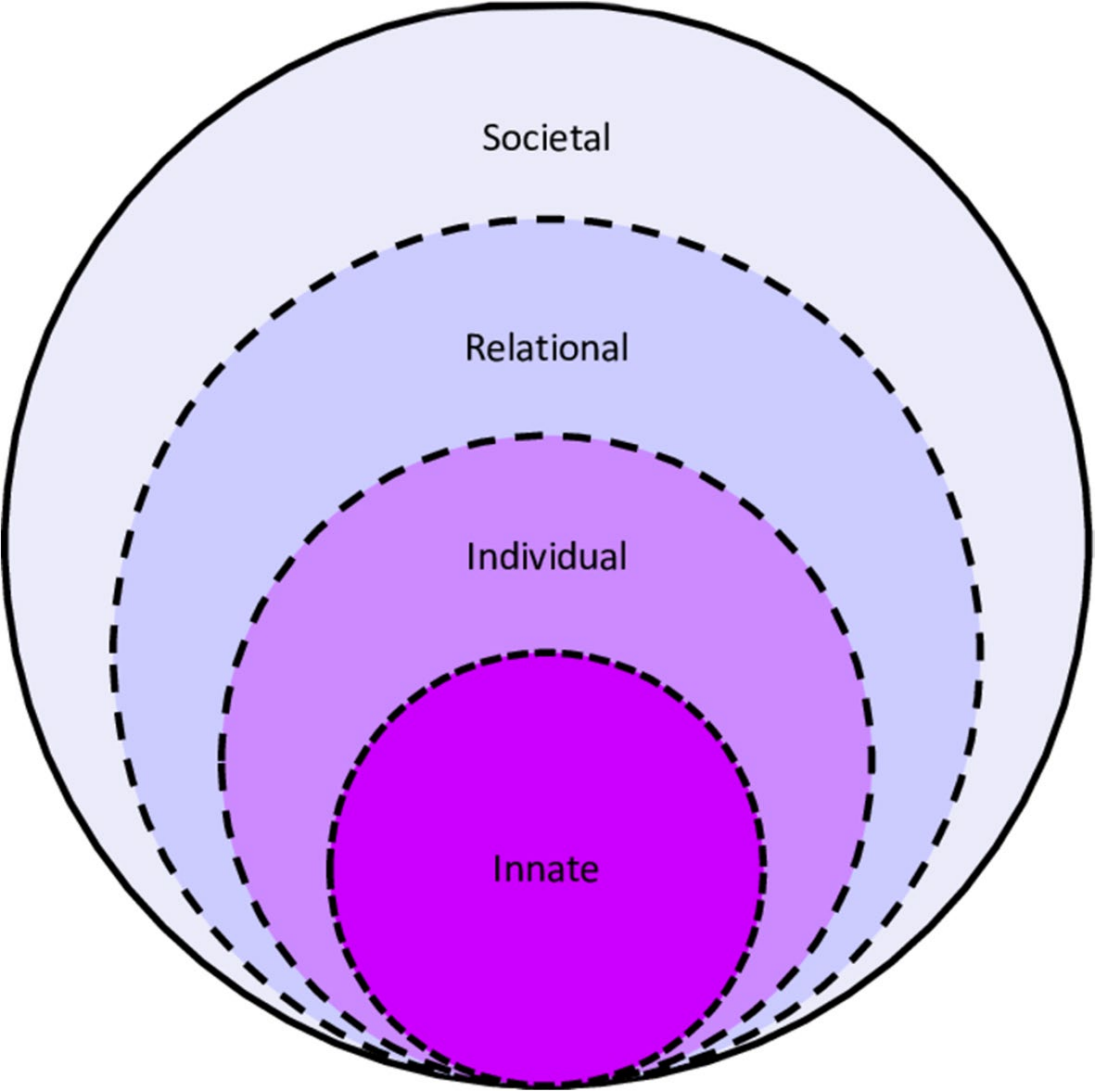
The ring theory of personhood [41]

We adopt a constructivist approach and relativist lens [26–35] to contend with the complex somato-psycho-social-semiotic perspectives of clinicians and facilitators in GNWR [19, 36–38]. This lens also allows us to build on Lim et al. [39]’s review on reflective writing and its siting of GNWR discussions within a structured CoP that promotes private, respectful and open discussions vital to the effective use of this approach. Lim et al. [39]’s review also found that reflective practice within a CoP supports the socialization process that inculcates the desired practice characteristics, beliefs, values and principles (henceforth belief systems); guides meaning-making of experiences, insights and new reflections; and ushers shifts in self-concepts of personhood and identity. These shifts can be envisaged through the Krishna-Pisupati Model of Professional Identity Formation (henceforth KPM). Viewing GNWR data through the lens of the KPM would lend support for GNWR as a viable alternative to tradition reflective practice. At the heart of the KPM is the Ring Theory of Personhood (RToP). The RToP posits that changes in the clinician’s belief system inspire shifts in self-concepts of identity and personhood (Fig. 1). The KPM further proposes that changes in the belief systems within the innate, individual, relational and societal rings feed changes in PIF [40].

**Fig. 2:**
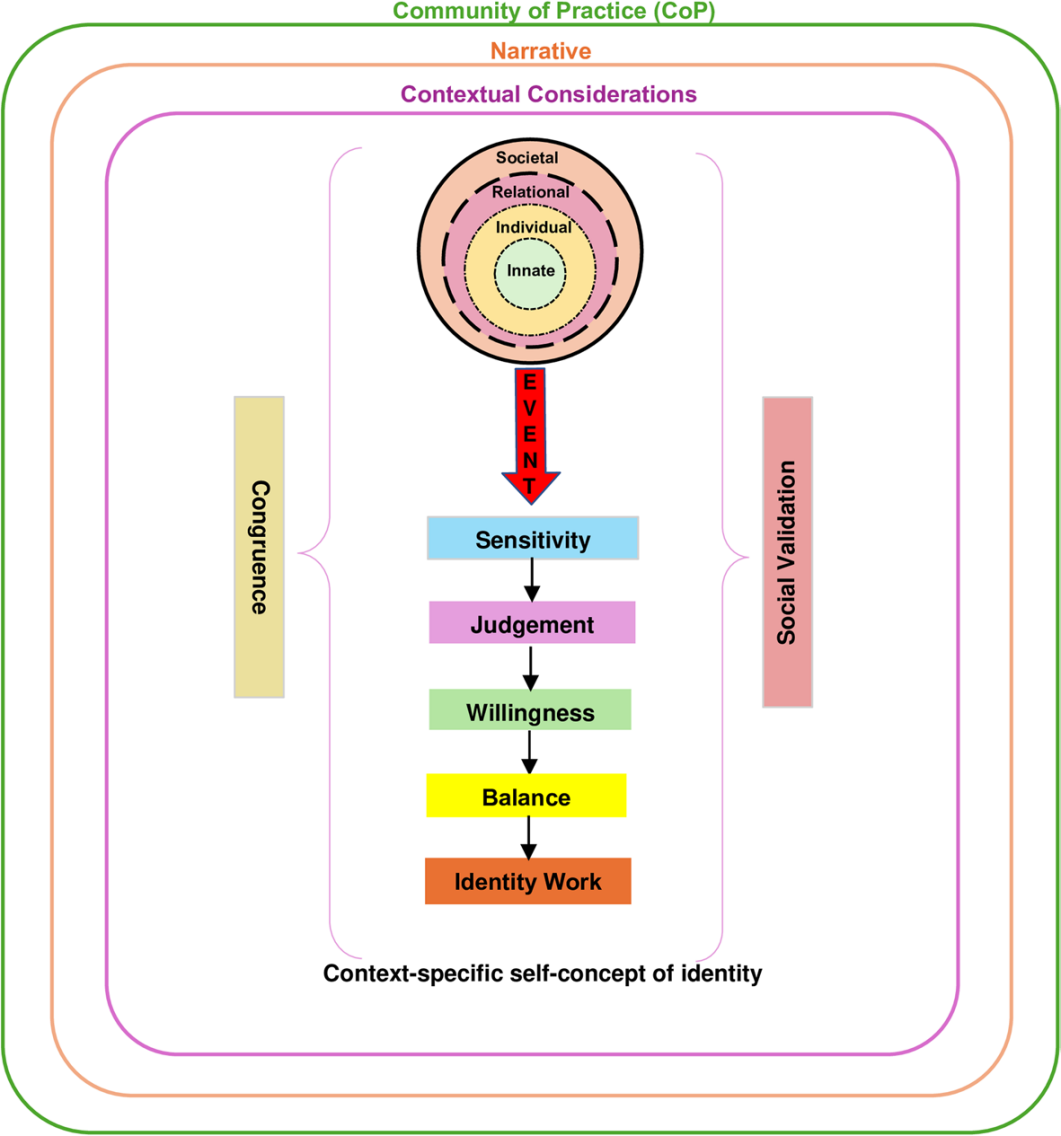
The Krishna-Pisupati model of professional identity formation [14]

The KPM goes on to explicate that norms, reflections, expectations, insights and considerations (collectively *life experiences*) may resonate or conflict with regnant religious and cultural belief systems in the innate ring; notions of autonomous function and individual characteristics behind the belief systems in the individual ring; the belief systems governing personal relationships housed within the relational ring; and/or the belief systems guiding peripheral relationships and societal, professional and legal expectations within the societal ring [41–44]. When detected (*sensitivity*)*,* the individual determines if these life experiences represent a threshold *event* and ascertains whether a response is required (*judgment*) and if they are willing, motivated and able to adapt their identity (*willingness*) [14]. *Balance* reflects the prioritization of these adaptations to preserve identity. The iterative process of *identity work* allow physicians to adapt their identity [14, 28] (Fig. 2)*.*

We posit that GNWR will help participants make sense and find meaning in their experiences shared in the facilitated group discussion. Making use of the KPM, we believe that GNWR would also fill the gaps in reshaping belief systems left by the lack of structured reflections in many programs.

### The systematic evidenced-based approach (SEBA)

A Systematic Scoping Review (SSR) was conducted to map the current use, structuring and assessment of GNWR in medical education. In particular, to facilitate the synthesis of a coherent narrative from multiple angles of GNWR, Krishna’s Systematic Evidence-based Approach (SEBA) was adopted to guide this SSR (henceforth SEBA-guided SSR). The six-staged SEBA methodology utilizes an expert team comprising medical librarians, local educational experts and clinicians to formulate, search and analyze the data; steer the synthesis of the findings; and review the conclusions drawn from each stage of the methodological process. Pivotally, the inclusion of the expert team serves to strengthen the accountability, reproducibility and structure of the review, as well as to attenuate personal biases in the interpretation of the data and its findings.

**Fig. 3:**
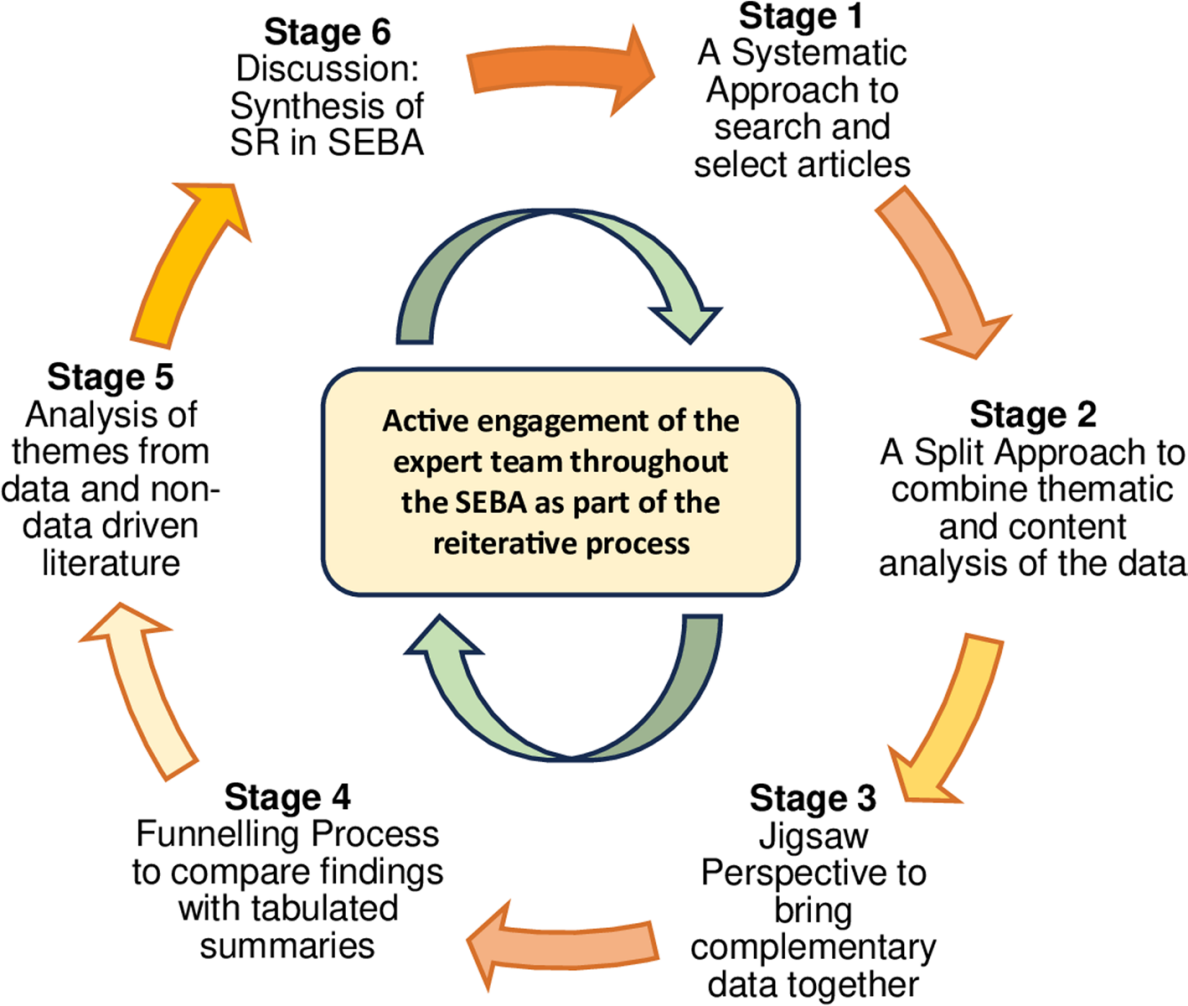
Stages of the systematic evidence-based approach [39]

Delineated in Fig. 3, the stages of SEBA are described in brief in the following section whilst a detailed description of the SEBA methodology is enclosed in Additional file 1. The SEBA-guided SSR meets the PRISMA-ScR criteria (see Additional file 2).

#### Stage 1 of SEBA: the systematic approach

Our primary research question*,* *“What is known of GNWR in medical education?”* and secondary research questions, *“How is GNWR structured and supported in medical education?”* and *“What are the outcomes of GNWR?”* were guided by a Population, Comparison and Context (PCC) framework (Table 1).

**Table 1:** Population, comparison and context (PCC), inclusion criteria and exclusion criteria applied to database search

	**Inclusion Criteria**	**Exclusion Criteria**
**Population**	Doctors in training positions and medical students	Allied health specialties such as dietetics, nursing, psychology, chiropractic, midwifery, social work Non-medical specialties such as clinical and translational science, veterinary, dentistry
**Comparison/ context**	Comparison of accounts and group non-written reflective practice (henceforth GNWR) approaches	

Here, the iterative process of the SEBA methodology also led to the addition of the following research objectives: *“How is GNWR assessed?”* and *“What barriers and facilitators exist to the applications of GNWR?”*.

#### Stage 2 of SEBA: split approach

The data from the searches were independently and concurrently analyzed by two teams using the Split Approach. One team adopted Braun and Clarke [45]’s approach to thematic analysis. This entailed the synthesis of codes from the ‘surface’ meaning of the included articles. Semantic themes were derived from ‘detail-rich’ codes [46] on different facets of GNWR, including its general principles of use, modalities, content, benefits, cons and enabling and hindering factors. With each member of the research team grouping the codes and listing the themes identified, online and in-person meetings were organised where consensus on the key themes was attained through *“negotiated consensual validation”* [47]. This practice of articulating, defending and persuading others of the strengths of their perspectives or relinquishing untenable views is key to reaching unanimity in such a collaborative research process [47]. Inter-rater reliability was not evaluated as the teams held regular meetings to discuss and compare their findings following their reviews of a specified number of similar articles.

Simultaneously, the second research team employed Hsieh and Shannon [48]’s approach to directed content analysis. This method utilized predetermined codes on GWNR drawn from Mann et al. [49]’s article entitled, *“Reflection and Reflective Practice in Health Professions Education: A Systematic Review”*, and Wald and Reis’ [50] *“Beyond the Margins: Reflective Writing and Development of Reflective Capacity in Medical Education”.* Text of similar meaning were classified into categories whilst any data uncaptured by the pre-existing codes were prescribed new ones. Consensus on the key categories was similarly achieved through *“negotiated consensual validation”* [47].

A third team of researchers prepared tabulated summaries of the included articles, with a focus on the study aims, key findings, methodology and conclusions (see Additional file 3). This ensures that vital aspects of the included articles were preserved.

#### Stage 3 of SEBA: the jigsaw perspective

Resting on the notion that complementary qualitative data gives *“a richer, more nuanced understanding of a given phenomenon”* [51] when reviewed together, the Jigsaw Perspective [52, 53] saw overlaps in themes and categories derived from the thematic and content analyses combined to create broader ‘themes/categories’—painting a more holistic picture of available data on GNWR. Here, the research team compared and grouped the themes and categories, based on the commensurate focus of the included articles from which they were derived. The similarity of the themes and categories enabled the use of reciprocal translation and mapping of the various themes/categories in Phase 6. A summary of the extracted data that formed the themes/categories is enclosed in Additional file 4.

#### Stage 4 of SEBA: the funnelling process

The Funnelling Process ensured that the resulting themes/categories were compared with the tabulated summaries of the included articles. This was to verify that the ‘jigsaw pieces’ appropriately echoed vital insights from the extant data and determine the consistency of the domains created. The resulting domains formed the basis of the ensuing discussion.

### The iterative process within SEBA

A key aspect of the SEBA process is its iterative process. Detection of the features of CoPs and the socialization process (discussed later in Table 2) that highlight the role of reflective practice on PIF led to the adoption of the KPM and use of this lens in the review of the data.

**Table 2:** Key features of communities of practice and the socialization process

Features of a CoP	References
Structured program	[2, 8, 10, 12, 15–19, 23–25, 36–38, 54–90]
A consistent approach	[9, 15–17, 19, 23–25, 36–38, 54–60, 62–67, 70, 71, 73–75, 77, 79, 80, 82, 83, 91–98]
Common objectives	[15, 16, 19, 24, 36–38, 54, 56, 58, 62, 64–66, 68, 71–73, 75, 78–80, 82, 91, 93, 95, 99, 100]
Nurturing environment	[12, 15, 58–60, 69, 74, 78, 91, 95, 101–105]
Consistent support	[37, 39, 99, 106]
**Features of the Socialization Process**	
Recognition of an *event*/sensitivity	[15, 18, 37, 56, 59, 65, 67–69, 74, 76, 79, 91, 94, 96, 107–112]
Judgment	[15, 17, 37, 54, 56, 58–60, 63, 64, 66, 67, 69, 71, 78, 79, 91, 93, 95, 107, 108]
Willingness	[17, 59, 62, 63, 78, 79, 94, 113]
Balance	[15, 19, 24, 36, 58, 59, 61, 74, 79, 84, 96, 108]
Identity work	[15, 36, 55, 56, 58, 64, 69, 76, 79, 108]

#### Stage 5 of SEBA: analysis of evidence-based and non-data driven literature

Whilst non-peer-reviewed or non-evidence-based grey literature comprise only a minority of the data sources (11 out of 98 included articles, or 11%), there remain concerns regarding the plausibility of grey literature biasing the synthesis of discussion. To assuage these concerns, the research team thematically analyzed and compared the themes from grey literature with that of research-based peer-reviewed data. Found to be similar in themes, data from grey literature was thus concluded to have unlikely influenced the data analysis process.

## Results

**Fig. 4:**
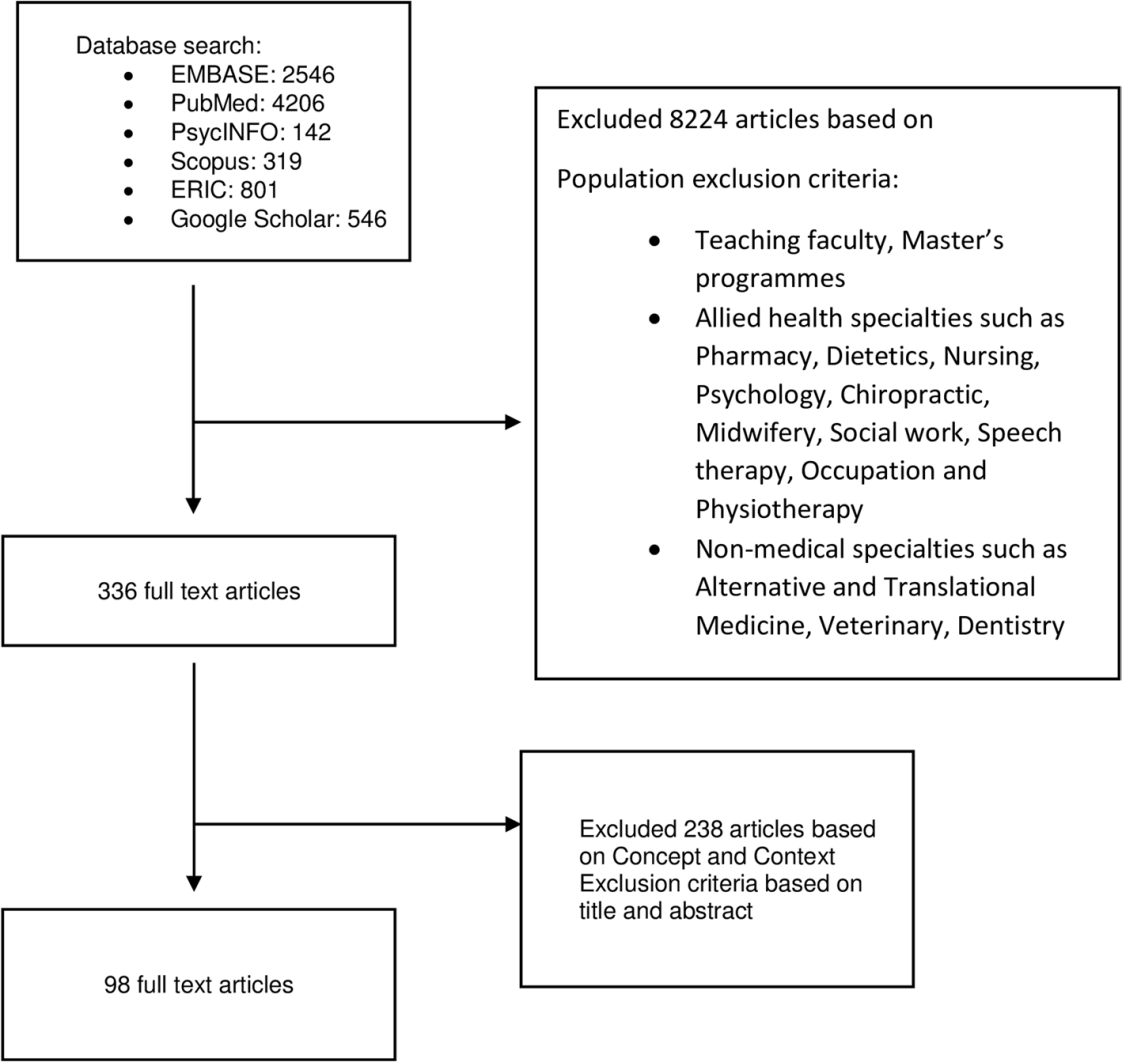
PRISMA-ScR flow chart

A total of 8560 abstracts and 336 full text articles were reviewed and 98 articles were included (Fig. 4). The domains identified were 1) Indications for use and their value; 2) Structure and how they can be used; 3) Models of reflective practice in GNWR; and 4) Features of communities of practice and the socialization process.

### Domain 1: Indications for use and their value

As a form of ‘disciplined self-surveillance’, GNWR impacts participants at a personal, professional and interprofessional level [54, 55, 114].

On a personal level, GNWR helps clinicians unburden their feelings of stress, anxiety and frustration whilst facilitating effective debriefs [17, 38, 55, 114] that afford facilitators the opportunity to address any potential maladaptive strategies [56, 57, 114] and institute appropriate supportive measures early [37, 38, 114]. Concurrently, GNWR moves clinicians away from ‘self-focused’ [58] reflections towards more holistic perspectives that hone greater appreciation of their personal values, growth [18, 59–61, 91, 114], learning styles, needs [62–64, 107], strengths and weaknesses [62], alongside improving emotional capacity [92] and empathy [15, 25, 36, 58, 63, 65–67, 92].

On a professional level, GNWR fosters ‘positive PIF’ [2, 16, 17, 56, 57, 62, 63, 68, 69, 93, 94, 107] by promoting greater self-awareness; boosting receptivity to feedback [95]; increasing adaptability [15, 17, 56, 96, 115], competencies [54, 56, 93], skills [55, 93] and professionalism [56, 61]; and providing clinicians with an avenue to apply their knowledge [18, 67, 70]. Further, GNWR enhances the appreciation of core ethical principles [68, 70, 114] and the ‘hidden curriculum’ [18, 61, 99, 114], as well as promotes desired professional attributes [62] and lifelong learning [15, 62, 70–72, 99, 115].

On an interprofessional level, GNWR strengthens interprofessional collaborations by promoting communication [24, 70], enhancing teamwork [18, 57, 59, 96] and boosting clinical competency [18, 68, 115] through a mix of role modelling [2, 55], mentoring [19, 73, 116, 117], supervision [118, 119], reflective dialogue [16, 61, 74, 120, 121], feedback [118] and experiential learning [69, 75, 114]. GNWR also aids clinicians to better understand their patient’s needs [25, 59, 61, 67, 76, 96] and the impact of the disease on patients and their families [18, 67, 96]. GNWR, in effect, boosts patient and interprofessional communication [18, 57, 59, 61, 96].

### Domain 2: Structure and how they can be used

GNWR may be used in tandem with different forms of individual [16, 19, 36] and group approaches [55, 68, 74, 99] and/or in combination with written reflections [60, 67, 73]. A consistent requirement, however, is a conducive, safe, open and supportive reflective environment [17, 19, 23, 37, 55, 63, 72, 74, 77, 78, 99] that safeguards confidentiality [17, 57, 70, 78]. This curated environment reflects local sociocultural, academic, clinical, professional and practical considerations [58, 74, 79]; facilitates alignment of expectations; boosts facilitator-clinician engagement [24, 37, 62, 71]; and provides dedicated time for reflective practice, feedback and debriefs [55, 62, 78, 97, 101]. Shaping such a program requires a structure around which the culture is built and clear boundaries to encapsulate them.

The ideal frequency of GNWR is open to debate with some arguing that bi-weekly interventions would cultivate more meaningful experiences [114, 122]. Durations of GNWR may range between 90–120 min [114]. Whilst a variety of mediums, including poetry [2], films [18] and lectures [108], have been proposed to platform these sessions, the through-line lies in their structured approach, consistent codes of conduct and a clear summary of program expectations [75]. These underline the importance of the facilitator’s motivations [62, 123, 124], skills, goals, availability [99] and ability [47] to build rapport and trust [62], as well as provide timely, personalized, appropriate and holistic feedback [17, 125, 126] and role modelling [37, 69, 73, 74].

Several tools have been used to assess GNWR’s impact on PIF. The validated Penn State College of Medicine Professionalism Questionnaire [33, 35], for instance, has been adopted as a pre-session and post-session survey for self-reporting attitudes regarding professionalism or key takeaways from reflective sessions [36]. Other frameworks such as the Brown Educational Guide to the Analysis of Narrative (BEGAN); the Reflection Evaluation For Learners’ Enhanced Competencies Tool (REFLECT); and the Self-reflection and Insight Scale (SRIS) for formative assessment of the reflective capacity within students [25, 37] are similarly employed to measure the impact of GNWR on PIF.

It is also imperative to recognize the prevailing barriers to effective GNWR. Prime amongst these are a lack of formal facilitator training, resulting in poorly selected, unmotivated, untrained and inexperienced facilitators [20–23] with poor attitudes that may compromise open discussions and frank exchange of ideas and honest reflections [15, 24–26]. Similarly, GNWR is hampered by poorly matched, untrained and unmotivated [33] participants with misaligned expectations who may show discomfort in sharing in a group setting [16, 28, 31–33] and/or in receiving feedback [27, 34]. Program-related factors, such as inadequate time for reflection [22, 27], unclear program goals [26, 28, 29] and the lack of a professional program assessment, also infringe on the quality of the reflections [29, 30].

### Domain 3: Models of reflective practice in GNWR

GNWR is a variation of reflective practice, with three main approaches.

Holmes et al. [23], describe the four-step reflective competency curriculum approach. This is centred on practice within a safe environment. It begins with *priming* clinicians to consider relevant scenarios whilst reflecting on related experiences. Clinicians are then told to self-monitor or keep diaries of stressors and their experiences [72, 80]. This stage of *noticing* gives way to *reflectin*g or sense-making. The final stage of *choosing* pivots on whether the insights and changes in thinking during meaning-making are to be integrated into their current repertoire of practices [36, 81, 91]. Cumulatively, this four-step reflective model serves to spotlight the internal motivations to conform to the hidden curriculum—that not only propagates desired attitudes and behaviors, but also inappropriate practices—promote transparency in existing and impending workplace pressures and co-devise strategies to make sense of past and current experiences [23]. Such reflective practice aids clinicians in withstanding the pressure to emulate unprofessional practices by negative role models and instead, engage in ‘positive deviance’, exemplified even in small acts, such as washing hands before entering a patient’s room despite the neglected practice by the rest of the team, or larger acts that include intervening when senior colleagues exhibit lapses in professional behaviors [23]. Through this reflective competency curriculum, effective PIF is concomitantly fostered as clinicians reflect and consider their own reasoning and decisions in *“enact[ing] best professional behaviors, innovat[ing] when appropriate and yet, resist[ing] conforming to complacency, overconfidence, and arrogance”* [23].

Spampinato et al. [2]’s adoption of Brookfield [127]’s steps of reflection sees clinicians analyze their assumptions, challenges, expected and intended conduct, response and/or behavior. This *assumption analysis* gives rise to *contextual* and *imaginative awareness* that ask clinicians how others would perceive and respond to the situation. Finally, clinicians engage in *reflective scepticism* on the conclusions they arrive at. This reflective framework was utilized in Spampinato et al. [2]’s professionalism case discussion intervention implemented during a gross anatomy course where case topics as such patient dehumanization, emotional suppression, teamwork and balance and sacrifice were discussed and reflected upon with first-year medical students. Whilst the intervention did not significantly increase the reflection scores, students conveyed their gratitude for a safe space to openly reflect and discuss professionalism issues associated with cadaver dissection, with 25 of 28 (89.2%) students reporting their recommendation for such sessions to be continued in subsequent anatomy courses [2].

Smith and Karban [12] draw attention to interprofessional reflections which promise due consideration of emotional or affective factors, power relationships and structures vis-a-vis socioeconomic considerations of a shared event, as well as re-evaluation of individual positions, actions and conduct in the face of new information [128]. This shift in reflecting on the self to broader issues beyond the individual is imperative in the evolving health and social care landscape that increasingly calls for stronger collaboration, communication and coordination between various professional groups. Failure to forge *“mutual trust and respect for different professional backgrounds”* [12] and dismantle *“the ‘othering’ of other professions within stereotyped expectations”* [12] may have deleterious consequences. Hodge [129] underlines this in the wake of the Inquiry into the death of Victoria Climbie in the United Kingdom. Interprofessional reflections also facilitate the development of the professional identity that transcends mere technical know-how. Such reflection engages with the individual’s ability to position oneself in the social, political and economic world where class, gender and race, for example, can varyingly account for treating illnesses and engagement with healthcare services [12]. Interprofessional reflections thereby nurture PIF by guiding clinicians to cultivate mutual understanding of the world and collaborative approaches through fostering common dialogue within CoPs [12].

Further building on these notions, van Braak et al. [19]’s Concentric circles of value, Lutz et al. [59]’s Clinical Reflective Training, O’Loughlin et al. [119]’s three stages of reflection that focused on ‘do, review and plan’ and Kolb [130]’s four-stage cycle all highlight inclusivity, diversity, safety and efficiency of collaborative reflections. These models also focus on uninterrupted sharing, replete with contextual associated attitudes, feelings, urgency and relevant professional standards and expectations [17]. At its heart, these models underline how reflection seeks to integrate new beliefs, values, principles, experiences, insights and skills into current belief systems to deconstruct *“traditional barriers, compartmentalised thinking and professional ‘tribalism’”* [12]. Further, they variously highlight the need for the exploration, discussion and conclusion stages [67, 91], as well as deeper insights into decisions to act and adapt belief systems, sense-making, refinement of thinking, adaptation of practice and shaping of conduct [36, 81, 91, 131] to enhance competence and nurture effective PIF.

### Domain 4: Features of communities of practice and the socialization process

Smith and Karban [12] suggest that effective GNWR works best as a CoP [1]. Within its structured program and curated environment, trusting relationships that are open to frank discussions, sharing, active listening and maintaining privacy and confidentiality may be forged [70, 78, 132]. These trusting relationships also provide an avenue to explore the challenges posed by the hidden curriculum [17, 23, 81, 114].

Inspired by the possibility that GNWR may pivot on CoPs and the socialization process, the expert and research teams reviewed the data for elements of these two entities (Table 2).

## Discussion

The Best Evidence Medical Education (BEME) Collaboration Guide and the Structured approach to the Reporting In healthcare education of Evidence Synthesis (STORIES) were used to guide Stage 6 of SEBA – discussion. This stage encompasses the synthesis of the scoping review in SEBA, reviewed by the expert team to boost balance and transparency and ensure that the conclusions drawn are practical, sustainable and adoptable within local practice setting.

In answering the primary research question, “*What is known of GNWR in medical educati*on*?”,* this SSR in SEBA affords a few key insights. To begin, reliance on the presence of a CoP extends beyond a safe, private and physical area for discussion, replete with protected time for reflection and meaning-making on a shared experience. Structure is key. This includes establishing and policing the codes of conduct, practice expectations, curated environment and approach used to guide the session and reflective cycle. Privacy, respectful engagement and the exchange of ideas must be facilitated, highlighting the role of the facilitator.

The role of the trained facilitator, their approach and the format taken to support the discussion and guide the reflective process have been stressed in the data. Facilitators also ought to participate in reflective dialogue and feedback [118] to consolidate and crystallise reflections, as well as support experiential learning.

**Fig. 5:**
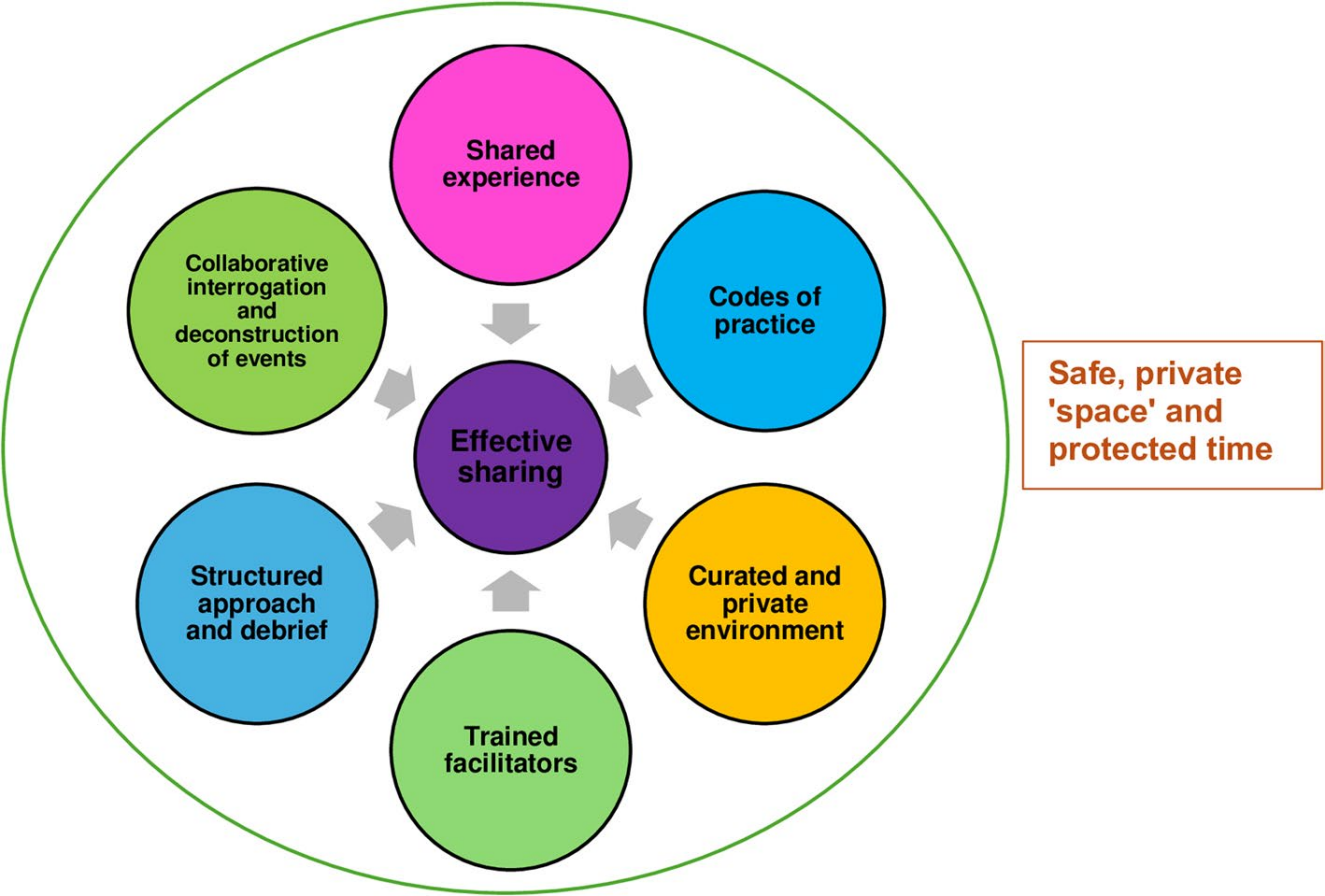
Wider concept of communities of practice in group non-written reflections

Cumulatively, these intrinsic factors come together to forward a wider concept of CoP in GNWR depicted in Fig. 5.

The process of introducing broader contextual considerations; integrating wider perspectives and guided-review of thinking and practice; and shifting belief systems and thus self-concepts of identity and personhood in GNWR invite comparisons with the socialization process. Indeed supported by a CoP-like structure, the reliance on trusting relationships to bolster timely, individualized, context-specific, appropriate and guided discussions and reflections vis-à-vis reviews and re-evaluations of shared events draw strong similarities to the socialization process. Moreover, GWNR’s clinically-relevant facilitation that caters to the participant’s abilities, needs, goals and opportunities, along with regnant psychoemotional, personal, relational, spiritual, existential and sociocultural effects surrounding the shared *event;* individualized, prompt and constructive feedback; context-specific advice; stage-specific, assessment-led coaching; and longitudinal, personalized, timely and holistic mentored support further underline similarities with the socialization process. Evidence of features of *sensitivity*, *judgment*, *willingness*, *balance* and *identity work* add credence to these comparisons [133] and draw comparisons to a threshold concept or *event* described in the KPM [134–136].

**Fig. 6:**
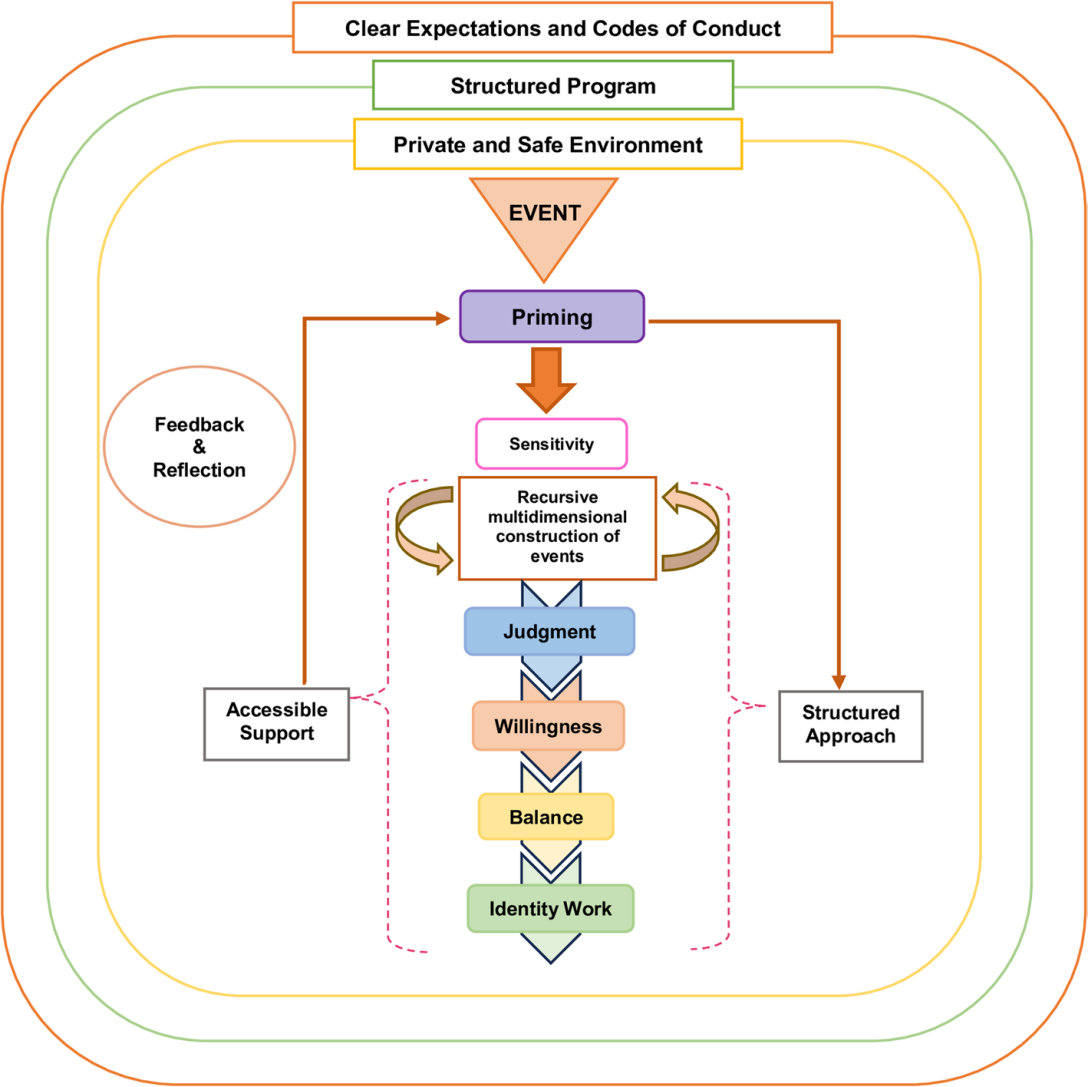
Adapted Krishna-Pisupati model in group non-written reflections

Building on the posit that GNWR functions as a CoP supporting the socialization process, we forward an adapted KPM model (Fig. 6). Here, there are critical differences. The KPM in GNWR highlights that the identification of a particular event is often predetermined in group reflections. However, rather than reducing the importance of *sensitivity*—directed by trained facilitators and guided gradually—imbuing the *event* with multidimensional perspectives helps with *priming* participants and focuses their *sensitivity* [137]. In some cases, frank exchange of ideas and reflections helps build a multidimensional perspective of the *event* imbued with the clinician’s personal perspectives and psychoemotional considerations, thus role modelling a holistic appreciation of an *event*. This role modelling by peers and facilitators also shape *judgment* and influence the clinician’s *willingness* to address the need for change in their belief systems [138]. Here, the presence of the same team members within familiar settings and a consistent set up would also likely hasten the shifting belief systems and practice of GNWR participants. These processes facilitate deeper reflections; re-evaluation of individual positions, actions and conduct; analysis of assumptions, challenges, expected and intended conduct, response and behavior; and/or sense-making. As Spampinato et al. [2] would suggest, this process will aid in *assumption analysis* and facilitate *contextual awareness* and *imaginative awareness* to enhance the exploration of context, power relationships and structures, socioeconomic considerations, exigency of the *event* and the need for its review and relevance, alongside current professional standards and regnant expectations [17, 67, 91]—therein shifting belief systems, thinking, feeling and conduct beyond what an individual’s skills and ‘technical rationalism’ would permit [12, 14–19]. Such ‘deeper dives’ underscore the importance of feedback, insights, and guided immersion by trained faculty within a structured GNWR process. Together, these processes map GNWR’s effects on PIF.

### Limitations of this review

Focus upon data published in English may have confined findings to practices in North America and Europe whilst neglecting evidence from Asian, South American and African settings which could inject greater sociocultural and contextual considerations into our results. Furthermore, introducing data from grey literature may also bias findings, particularly when such accounts are prone to reporting bias.

### Recommendations

The findings of this SSR in SEBA provides an opportunity to structure effective use of GNWR. These include the following:

**Table:** 

1. Host organizations [104] should allocate dedicated time (45–120 min) [55, 61] for reflective sessions in order to provide an adequate platform for learners to concentrate on reflective practices
2. A conducive environment [17, 55, 58, 101] for sharing and maintenance of privacy needs to be created. Van Braak et al. [19] suggest that inclusivity, diversity, safety and efficiency must be supported
3. Consistent membership of GNWR sessions should be encouraged to build rapport, enhance collaborative interrogation and deconstruction of events [19, 126] and foster better reflective sessions. Smith and Karban [12] suggest that these groups should be considered CoPs
4. The timing, duration and method of reflection should be established [71], as should the setting [84, 126]
5. The group sessions [55, 90, 106] may be structured on Balint Groups [17, 59, 77, 119]. Here, a mix of [76, 80, 81, 117, 139] individual [15, 56, 72, 79, 104, 140] and group written and GNWR may be employed
6. New participants should be provided with the role and benefits [126], aims, structure, and expectations of GNWRs [125, 138]. Where possible, the approach and questions used should be consistent [114]. A clearly established curriculum [115] with specified contents, expectations, learning objectives and codes of practice can be used to guide case-based discussions [2, 73, 115, 119], Schwartz Rounds [63] vignette-focused approach [2], or used in tandem with flashcards [114]. This helps align expectations
7. Carefully selected participants must be motivated [12, 54], invested in the process, self- directed and display self-awareness [131]
8. Learners should be pre-empted with contextualized, specific [114, 121] and meaningful events [19, 55, 82] to stimulate thoughts [125, 138]
9. The reflections should be facilitated by trained faculty [17, 25, 80, 88, 101, 114, 126, 141–143]. The process [55] needs to be structured, interactive and flexible, exemplified by Brookfield’s steps of reflection [2, 127], van Braak et al. [19]’s Concentric circles of value and Clinical Reflective Training [59]
10. GNWRs may be a mix of role modelling [2, 55], mentoring [19, 73, 116, 117], supervision [118, 119], reflective dialogue [16, 61, 74, 120], feedback [118] and experiential learning
11. Reflections should be timely reviewed by facilitators [2]
12. Assessment tools such as the Self-reflection and Insight scale [83] may be employed, though this should only be to guide discussions [17]
13. Specific time should be set aside for individual debriefs and to address individual emotional responses and distress, as well as to discuss issues and answer points of clarification [126]
14. Follow-up action after the reflective sessions and extending lessons learnt to medical education should be encouraged alongside daily practices to promote greater maintenance of reflective practices amongst learners

## Conclusion

In scoping what is known on the current practice of GNWR and how it is structured and supported, this SSR in SEBA has synthesised a practical evidence-guided scaffold for the application of GNWR in practice. With implications on PIF, our guide also highlights effective assessment and facilitation approaches, as well as means of circumnavigating some of the key barriers to the use of GNWR.

We acknowledge that closer evaluations of the dynamics within GNWR discussions and facilitating styles are required, as is a deeper study of the impact of GNWR on an individual’s PIF. This then will be the focus of our forthcoming studies as we look forward to engaging further in this expanding area of medical education.

## Supplementary Information

Additional file 1. Full SEBA Methodology

Additional file 2. PRISMA-ScR Checklist

Additional file 3. Tabulated Summaries of Included Full-Text Articles

Additional file 4. Summary of Extracted Data

## Data Availability

The datasets supporting the conclusions of this article are included within the article and its additional files.

## Abbreviations

PIF: Professional Identity Formation
GNWR: Group Non-Written Reflections
CoP: Community of Practice
KPM: Krishna-Pisupati Model of Professional Identity Formation
RToP: Ring Theory of Personhood
SSR: Systematic Scoping Review
SEBA: Systematic Evidence-Based Approach
PCC: Population, Comparison and Context

## References

[1] Barab S, Makinster J. Designing system dualities: Characterizing an online professional development community. In: Barab S, Kling R, Gray J, editors. Designing for virtual communities in the service of learninges. Cambridge, UK: Cambridge University Press; 2004. p. 53–90.

[2] Spampinato CM, Wittich CM, Beckman TJ, Cha SS, Pawlina W. “Safe harbor”: Evaluation of a professionalism case discussion intervention for the gross anatomy course. Anat Sci Educ. 2014;7(3):191–8. 24039220 10.1002/ase.1395

[3] Mezirow J. Transformative learning: Theory to practice. In: Cranton P, editor. Transformative learning in action: Insights from practice. 1st ed. San Francisco, CA: Jossey-Bass; 1997. p. 5–12.

[4] Kember D, Leung D, Jones A, Loke AY, McKay J, Sinclair K, et al. Development of a questionnaire to measure the level of reflective thinking. Assess Eval High Educ. 2000;25:381–95.

[5] Wittich CM, Reed DA, McDonald FS, Varkey P, Beckman TJ. Perspective: Transformative learning: a framework using critical reflection to link the improvement competencies in graduate medical education. Acad Med. 2010;85(11):1790–3. 20881823 10.1097/ACM.0b013e3181f54eed

[6] Stark P, Roberts C, Newble D, Bax N. Discovering professionalism through guided reflection. Med Teach. 2006;28(1):e25–31. 16627318 10.1080/01421590600568520

[7] Wilkinson TJ, Wade WB, Knock LD. A blueprint to assess professionalism: Results of a systematic review. Acad Med. 2009;84(5):551–8. 19704185 10.1097/ACM.0b013e31819fbaa2

[8] Aronson L. Twelve tips for teaching reflection at all levels of medical education. Med Teach. 2011;33(3):200–5. 20874014 10.3109/0142159X.2010.507714

[9] Sandars J. The use of reflection in medical education: AMEE guide no. 44. Med Teach. 2009;31(8):685–95. 19811204 10.1080/01421590903050374

[10] Uygur J, Stuart E, De Paor M, Wallace E, Duffy S, O’Shea M, et al. A best evidence in medical education systematic review to determine the most effective teaching methods that develop reflection in medical students: BEME guide no. 51. Med Teach. 2019;41(1):3–16. 30634872 10.1080/0142159X.2018.1505037

[11] Hargreaves K. Reflection in medical education. J Univ Teach Learn Pract. 2016;13(2):6.

[12] Smith S, Karban K. Developing critical reflection within an interprofessional learning programme. 36th Annual SCUTREA Conference; 04 July 2006 - 06 April 2006. Leeds: Leeds Beckett University; 2006. p. 1–13.

[13] Than ZO, Chang MA, Sim SW, Krishna LKR. The role of the multidisciplinary team in decision making at the end of life. Adv Med Ethics. 2015;2(2).

[14] Teo KJH, Teo MYK, Pisupati A, Ong RSR, Goh CK, Seah CHX, et al. Assessing professional identity formation (PIF) amongst medical students in oncology and palliative medicine postings: a SEBA guided scoping review. BMC Palliat Care. 2022;21(1):200. 36397067 10.1186/s12904-022-01090-4 PMC9673314

[15] Scheide L, Teufel D, Wijnen-Meijer M, Berberat PO. (self-)reflexion and training of professional skills in the context of “being a doctor” in the future - a qualitative analysis of medical students’ experience in let me ... Keep you real! GMS J Med Educ. 2020;37(5):Doc47. 32984506 10.3205/zma001340 PMC7499461

[16] Haidet P, Hatem DS, Fecile ML, Stein HF, Haley HL, Kimmel B, et al. The role of relationships in the professional formation of physicians: Case report and illustration of an elicitation technique. Patient Educ Couns. 2008;72(3):382–7. 18619760 10.1016/j.pec.2008.05.016

[17] Lack L, Yielder J, Goodyear-Smith F. Evaluation of a compulsory reflective group for medical students. J Prim Health Care. 2019;11(3):227–34. 32171375 10.1071/HC18030

[18] Lumlertgul N, Kijpaisalratana N, Pityaratstian N, Wangsaturaka D. Cinemeducation: A pilot student project using movies to help students learn medical professionalism. Med Teach. 2009;31(7):e327–332. 19811142 10.1080/01421590802637941

[19] van Braak M, Giroldi E, Huiskes M, Diemers AD, Veen M, van den Berg P. A participant perspective on collaborative reflection: Video-stimulated interviews show what residents value and why. Adv Health Sci Educ Theory Pract. 2021;26(3):865–79. 33590384 10.1007/s10459-020-10026-7 PMC8338865

[20] Lee FQH, Chua WJ, Cheong CWS, Tay KT, Hian EKY, Chin AMC, et al. A systematic scoping review of ethical issues in mentoring in surgery. J Med Educ Curric Dev. 2019;6:2382120519888915. 31903425 10.1177/2382120519888915 PMC6923696

[21] Cheong CWS, Chia EWY, Tay KT, Chua WJ, Lee FQH, Koh EYH, et al. A systematic scoping review of ethical issues in mentoring in internal medicine, family medicine and academic medicine. Adv Health Sci Educ Theory Pract. 2020;25(2):415–39. 31705429 10.1007/s10459-019-09934-0

[22] Feudtner C, Christakis DA. Making the rounds. The ethical development of medical students in the context of clinical rotations. Hastings Cent Rep. 1994;24(1):6–12. 8045775

[23] Holmes CL, Harris IB, Schwartz AJ, Regehr G. Harnessing the hidden curriculum: a four-step approach to developing and reinforcing reflective competencies in medical clinical clerkship. Adv Health Sci Educ Theory Pract. 2015;20(5):1355–70. 25319835 10.1007/s10459-014-9558-9

[24] Zumwalt AC. Anticipatory feelings about dissection: An exercise for the first day of a gross anatomy course. Anat Sci Educ. 2021;14(6):828–35. 33369234 10.1002/ase.2048

[25] Green MJ. Comics and medicine: peering into the process of professional identity formation. Acad Med. 2015;90(6):774–9. 25853686 10.1097/ACM.0000000000000703

[26] Ng Y, Koh Z, Yap H, Tay K, Tan X, Ong Y. Assessing mentoring: A scoping review of mentoring assessment tools in internal medicine between 1990 and 2019. PLoS One. 2020;8(5):e0232511. 10.1371/journal.pone.0232511 PMC7209188 32384090

[27] Bok C, Ng C, Koh J, Ong Z, Ghazali H, Tan L. Interprofessional communication (IPC) for medical students: a scoping review. BMC Med Educ. 2020;20(1):372. 33081781 10.1186/s12909-020-02296-x PMC7574565

[28] Ngiam L, Ong Y, Ng J, Kuek J, Chia J, Chan N. Impact of caring for terminally ill children on physicians: a systematic scoping review. Am J Hosp Palliat Care. 2020;38(4):396–418. 32815393 10.1177/1049909120950301

[29] Krishna L, Tan L, Ong Y, Tay K, Hee J, Chiam M. Enhancing mentoring in palliative care: an evidence based mentoring framework. J Med Educ Curric Dev. 2020;7:2382120520957649. 33015366 10.1177/2382120520957649 PMC7517982

[30] Wyatt T, Rockich-Winston N, White D, Taylor T. “Changing the narrative”: A study on professional identity formation among Black/African American physicians in the U.S. Adv Health Sci Educ Theory Pract. 2021;26(1):183–98. 32572728 10.1007/s10459-020-09978-7

[31] Crotty M. The foundations of social research: Meaning and perspective in the research process. 1st ed. New South Wales, Australia: SAGE Publications; 1998. p. 256.

[32] Pring R. The ‘false dualism’ of educational research. J Philos Educ. 2000;34(2):247–60.

[33] Ford D, Downey L, Engelberg R, Back A, Curtis J. Discussing religion and spirituality is an advanced communication skill: an exploratory structural equation model of physician trainee self-ratings. J Palliat Med. 2012;15(1):63–70. 22242716 10.1089/jpm.2011.0168

[34] Schick-Makaroff K, MacDonald M, Plummer M, Burgess J, Neander W. What synthesis methodology should I use? A review and analysis of approaches to research synthesis. AIMS Public Health. 2016;3(1):172–215. 29546155 10.3934/publichealth.2016.1.172 PMC5690272

[35] Deshpande A, Sanders Thompson V, Vaughn K, Kreuter M. The use of sociocultural constructs in cancer screening research among African Americans. Cancer Control. 2009;16(3):256–65. 19556966 10.1177/107327480901600308 PMC3614350

[36] Tait GR, Hodges BD. Residents learning from a narrative experience with dying patients: a qualitative study. Adv Health Sci Educ Theory Pract. 2013;18(4):727–43. 23053870 10.1007/s10459-012-9411-y

[37] Wald HS, Anthony D, Hutchinson TA, Liben S, Smilovitch M, Donato AA. Professional identity formation in medical education for humanistic, resilient physicians: pedagogic strategies for bridging theory to practice. Acad Med. 2015;90(6):753–60. 25901874 10.1097/ACM.0000000000000725

[38] Hall P, Byszewski A, Sutherland S, Stodel EJ. Developing a sustainable electronic portfolio (eportfolio) program that fosters reflective practice and incorporates CanMEDS competencies into the undergraduate medical curriculum. Acad Med. 2012;87(6):744–51. 22534601 10.1097/ACM.0b013e318253dacd

[39] Lim JY, Ong SYK, Ng CYH, Chan KLE, Wu SYEA, So WZ, et al. A systematic scoping review of reflective writing in medical education. BMC Med Educ. 2023;23(1):12. 36624494 10.1186/s12909-022-03924-4 PMC9830881

[40] Krishna LKR. Accounting for personhood in palliative sedation: The ring theory of personhood. Med Humanit. 2014;40(1):17–21. 24072720 10.1136/medhum-2013-010368

[41] Ong RSR, Wong RSM, Chee RCH, Quek CWN, Burla N, Loh CYL, et al. A systematic scoping review moral distress amongst medical students. BMC Med Educ. 2022;22(1):466. 35710490 10.1186/s12909-022-03515-3 PMC9203147

[42] Ho CY, Kow CS, Chia CHJ, Low JY, Lai YHM, Lauw SK, et al. The impact of death and dying on the personhood of medical students: A systematic scoping review. BMC Med Educ. 2020;20(1):516. 33371878 10.1186/s12909-020-02411-y PMC7768997

[43] Chan NPX, Chia JL, Ho CY, Ngiam LXL, Kuek JTY, Ahmad Kamal NHB, et al. Extending the ring theory of personhood to the care of dying patients in intensive care units. Asian Bioeth Rev. 2021;14(1):1–16. 34691261 10.1007/s41649-021-00192-0 PMC8526529

[44] Kuek JTY, Ngiam LXL, Kamal NHA, Chia JL, Chan NPX, Abdurrahman ABHM, et al. The impact of caring for dying patients in intensive care units on a physician’s personhood: a systematic scoping review. Philos Ethics Humanit Med. 2020;15(1):12. 33234133 10.1186/s13010-020-00096-1 PMC7685911

[45] Braun V, Clarke V. Using thematic analysis in psychology. Qual Res Psychol. 2006;3(2):77–101.

[46] Tan YS, Teo SWA, Pei Y, Sng JH, Yap HW, Toh YP, et al. A framework for mentoring of medical students: thematic analysis of mentoring programmes between 2000 and 2015. Adv Health Sci Educ Theory Pract. 2018;23(4):671–97. 29550907 10.1007/s10459-018-9821-6

[47] Sandelowski M, Barroso J. Handbook for synthesizing qualitative research. New York, NY: Springer Publishing Company; 2006. p. 284.

[48] Hsieh H-F, Shannon SE. Three approaches to qualitative content analysis. Qual Health Res. 2005;15(9):1277–88. 16204405 10.1177/1049732305276687

[49] Mann K, Gordon J, MacLeod A. Reflection and reflective practice in health professions education: a systematic review. Adv in Health Sci Educ Theory Pract. 2009;14(4):595–621. 18034364 10.1007/s10459-007-9090-2

[50] Wald HS, Reis SP. Beyond the margins: Reflective writing and development of reflective capacity in medical education. J Gen Intern Med. 2010;25(7):746–9. 20407840 10.1007/s11606-010-1347-4 PMC2881974

[51] Moss PA, Haertel EH. Engaging methodological pluralism. In: Gitomer D, Bell C, editors. Handbook of research on teaching. Washington, DC: AERA; 2016. p. 127–247.

[52] France EF, Wells M, Lang H, Williams B. Why, when and how to update a meta-ethnography qualitative synthesis. Syst Rev. 2016;5(1):44. 26979748 10.1186/s13643-016-0218-4 PMC4791806

[53] Noblit GW, Hare RD. In: Maanen JV, Manning PK, Miller ML, editors. Meta-ethnography: Synthesizing qualitative studies. Newbury Park, CA: Sage Publications; 1988. p. 88.

[54] McNeill H, Brown JM, Shaw NJ. First year specialist trainees’ engagement with reflective practice in the e-portfolio. Adv Health Sci Educ Theory Pract. 2010;15(4):547–58. 20058073 10.1007/s10459-009-9217-8

[55] Nothnagle M, Reis S, Goldman RE, Anandarajah G. Fostering professional formation in residency: Development and evaluation of the “forum” seminar series. Teach Learn Med. 2014;26(3):230–8. 25010233 10.1080/10401334.2014.910124

[56] Shiozawa T, Glauben M, Banzhaf M, Griewatz J, Hirt B, Zipfel S, et al. An insight into professional identity formation: qualitative analyses of two reflection interventions during the dissection course. Anat Sci Educ. 2020;13(3):320–32. 31509334 10.1002/ase.1917

[57] Sheehan D, Wilkinson TJ, Bowie E. Becoming a practitioner: Workplace learning during the junior doctor’s first year. Med Teach. 2012;34(11):936–45. 22938684 10.3109/0142159X.2012.717184

[58] Rucker L, Shapiro J. Becoming a physician: Students’ creative projects in a third-year im clerkship. Acad Med. 2003;78(4):391–7. 12691972 10.1097/00001888-200304000-00015

[59] Lutz G, Scheffer C, Edelhaeuser F, Tauschel D, Neumann M. A reflective practice intervention for professional development, reduced stress and improved patient care - a qualitative developmental evaluation. Patient Educ Couns. 2013;92(3):337–45. 23642894 10.1016/j.pec.2013.03.020

[60] Gordon J. Assessing students’ personal and professional development using portfolios and interviews. Med Educ. 2003;37(4):335–40. 12654118 10.1046/j.1365-2923.2003.01475.x

[61] Branch WT Jr, George M. Reflection-based learning for professional ethical formation. AMA J Ethics. 2017;19(4):349–56. 28430568 10.1001/journalofethics.2017.19.4.medu1-1704

[62] Schrempf S, Herrigel L, Pohlmann J, Griewatz J, Lammerding-Köppel M. Everybody is able to reflect, or aren’t they? Evaluating the development of medical professionalism via a longitudinal portfolio mentoring program from a student perspective. GMS J Med Educ. 2022;39(1):Doc12. 35368842 10.3205/zma001533 PMC8953193

[63] Stocker C, Cooney A, Thomas P, Kumaravel B, Langlands K, Hearn J. Schwartz rounds in undergraduate medical education facilitates active reflection and individual identification of learning need. MedEdPublish. 2018;7:230. 38089201 10.15694/mep.2018.0000230.1 PMC10712006

[64] Alizadeh M, Mirzazadeh A, Parmelee DX, Peyton E, Mehrdad N, Janani L, et al. Leadership identity development through reflection and feedback in team-based learning medical student teams. Teach Learn Med. 2018;30(1):76–83. 28753047 10.1080/10401334.2017.1331134

[65] Lid TG, Eraker R, Malterud K. “I recognise myself in that situation…” using photographs to encourage reflection in general practitioners. BMJ. 2004;329(7480):1488–90. 15604194 10.1136/bmj.329.7480.1488 PMC535994

[66] Markham Jr F, Westlake E, DeSantis J. The effect of reflection rounds on medical students’ empathy. Med Res Arch. 2021;9(7).

[67] Chua IS, Bogetz AL, Bhansali P, Long M, Holbreich R, Kind T, et al. The patient experience debrief interview: How conversations with hospitalized families influence medical student learning and reflection. Acad Med. 2019;94(11S):S86–94. 31365398 10.1097/ACM.0000000000002914

[68] Xu D, Atkinson M, Yap T, Yap M, Hossain R, Chong F, et al. Reflecting on exchange students’ learning: Structure, objectives and supervision. Med Teach. 2020;42(3):278–84. 31718353 10.1080/0142159X.2019.1676886

[69] Branch WT Jr. The road to professionalism: Reflective practice and reflective learning. Patient Educ Couns. 2010;80(3):327–32. 20570461 10.1016/j.pec.2010.04.022

[70] Ohta R, Sano C. Reflection in rural family medicine education. Int J Environ Res Public Health. 2022;19(9):5137. 35564531 10.3390/ijerph19095137 PMC9100794

[71] Goldie J, Dowie A, Cotton P, Morrison J. Teaching professionalism in the early years of a medical curriculum: a qualitative study. Med Educ. 2007;41(6):610–7. 17518842 10.1111/j.1365-2923.2007.02772.x

[72] Riskin A, Kerem NC, Van-Raalte R, Kaffman M, Yakov G, Aizenbud D, et al. ’Becoming a physician’-medical students get acquainted with disadvantaged populations, and practise sensitive and effective communication. Perspect Med Educ. 2015;4(6):339–43. 26481394 10.1007/s40037-015-0222-8 PMC4673065

[73] Matsuyama Y, Nakaya M, Leppink J, van der Vleuten C, Asada Y, Lebowitz AJ, et al. Limited effects from professional identity formation-oriented intervention on self-regulated learning in a preclinical setting: a randomized-controlled study in Japan. BMC Med Educ. 2021;21(1):30. 33413338 10.1186/s12909-020-02460-3 PMC7791888

[74] Lutz G, Pankoke N, Goldblatt H, Hofmann M, Zupanic M. Enhancing medical students’ reflectivity in mentoring groups for professional development - a qualitative analysis. BMC Med Educ. 2017;17(1):122. 28709462 10.1186/s12909-017-0951-y PMC5512833

[75] Rousseau A, Saucier D, Côté L. Introduction to core competencies in residency: A description of an intensive, integrated, multispecialty teaching program. Acad Med. 2007;82(6):563–8. 17525540 10.1097/ACM.0b013e3180555b29

[76] Levi BH, Green MJ. Humanities in full retreat. Teach Learn Med. 2003;15(4):252–6. 14612258 10.1207/S15328015TLM1504_07

[77] Schulz C, Wenzel-Meyburg U, Karger A, Scherg A, der InSchmitten J, Trapp T, et al. Implementation of palliative care as a mandatory cross-disciplinary subject (QB13) at the medical faculty of the Heinrich-Heine-University Düsseldorf, Germany. GMS Z Med Ausbild. 2015;32(1):6. 10.3205/zma000948 PMC4330636 25699109

[78] Dijkhuizen K, Bustraan J, de Beaufort AJ, Velthuis SI, Driessen EW, van Lith JMM. Encouraging residents’ professional development and career planning: the role of a development-oriented performance assessment. BMC Med Educ. 2018;18(1):207. 30185174 10.1186/s12909-018-1317-9 PMC6125996

[79] Jarvis-Selinger S, MacNeil KA, Costello GRL, Lee K, Holmes CL. Understanding professional identity formation in early clerkship: a novel framework. Acad Med. 2019;94(10):1574–80. 31192797 10.1097/ACM.0000000000002835

[80] Reis SP, Wald HS. Contemplating medicine during the third reich: Scaffolding professional identity formation for medical students. Acad Med. 2015;90(6):770–3. 25853685 10.1097/ACM.0000000000000716

[81] Soo J, Brett-MacLean P, Cave M-T, Oswald A. At the precipice: A prospective exploration of medical students’ expectations of the pre-clerkship to clerkship transition. Adv Health Sci Educ Theory Pract. 2016;21(1):141–62. 26164285 10.1007/s10459-015-9620-2

[82] Svantesson M, Löfmark R, Thorsén H, Kallenberg K, Ahlström G. Learning a way through ethical problems: Swedish nurses’ and doctors’ experiences from one model of ethics rounds. J Med Ethics. 2008;34(5):399–406. 18448726 10.1136/jme.2006.019810

[83] Roberts C, Stark P. Readiness for self-directed change in professional behaviours: factorial validation of the self-reflection and insight scale. Med Educ. 2008;42(11):1054–63. 19141007 10.1111/j.1365-2923.2008.03156.x

[84] Raut AV, Gupta SS. Reflection and peer feedback for augmenting emotional intelligence among undergraduate students: a quasi-experimental study from a rural medical college in central India. Educ Health (Abingdon). 2019;32(1):3–10. 31512586 10.4103/efh.EfH_31_17

[85] Park J, Woodrow SI, Reznick RK, Beales J, MacRae HM. Observation, reflection, and reinforcement: surgery faculty members’ and residents’ perceptions of how they learned professionalism. Acad Med. 2010;85(1):134–9. 20042839 10.1097/ACM.0b013e3181c47b25

[86] Maitra A, Lin S, Rydel T, Schillinger E. Balancing forces: Medical students’ reflections on professionalism challenges and professional identity formation. Fam Med. 2021;53(3):200–6. 33723818 10.22454/FamMed.2021.128713

[87] Dixon WW, Gallegos M, Williams SR. Coaching pilot enhances professional identity formation and clinical skills in clerkship shortened by coronavirus. Acad Emerg Med. 2021;28(SUPPL 1):S364–5. 10.5811/westjem.2021.12.53917 PMC8782139 35060857

[88] Chambers S, Brosnan C, Hassell A. Introducing medical students to reflective practice. Educ Prim Care. 2011;22(2):100–5. 21439141 10.1080/14739879.2011.11493975

[89] Bennett D, Kelly M, O'Flynn S. CanMEDS reflect? Mapping student reflections on professionalism to a competency framework.Association for the Study of Medical Education (ASME); July 2011; Edinburgh, Scotland; 2011. p. 45-46.

[90] Allison CJ, Pullen GP. Student discussion groups on doctor-patient relationships: A critical assessment. Med Educ. 1981;15(6):392–7. 7329366 10.1111/j.1365-2923.1981.tb02421.x

[91] Zuo SW, Cichowitz C, Shochet R, Venkatesan A. Peer-led, postanatomy reflection exercise in dissection teams: Curriculum and training materials. MedEdPORTAL. 2017;13:10565. 30800767 10.15766/mep_2374-8265.10565 PMC6342053

[92] Daryazadeh S, Adibi P, Yamani N, Mollabashi R. Impact of narrative medicine program on improving reflective capacity and empathy of medical students in Iran. J Educ Eval Health Prof. 2020;17:3. 31986248 10.3352/jeehp.2020.17.3 PMC7061215

[93] Taylor DA, Gorski V, Burge SK. Using reflections to evaluate the stfm behavioral science/family systems educator fellowship. Fam Med. 2017;49(7):522–6. 28724149

[94] Wyatt TR, Kleinheksel AJ, Tews M. Linking patient care ownership and professional identity formation through simulation. Teach Learn Med. 2021;33(2):164–72. 33840311 10.1080/10401334.2020.1813583

[95] Raski B, Eissner A, Gummersbach E, Wilm S, Hempel L, Dederichs M, et al. Implementation of online peer feedback for student self-reflection–first steps on the development of a feedback culture at a medical faculty. GMS J Med Educ. 2019;36(4):Doc42. 31544142 10.3205/zma001250 PMC6737261

[96] Choo Hwee P, Hwee Sing K, Yong Hwang MK, Mei AHY. The informal curriculum: What do junior doctors learn from a palliative care rotation? BMJ Support Palliat Care. 2020;10(1):114–7. 10.1136/bmjspcare-2018-001625 30425051

[97] Wittich CM, Pawlina W, Drake RL, Szostek JH, Reed DA, Lachman N, et al. Validation of a method for measuring medical students’ critical reflections on professionalism in gross anatomy. Anat Sci Educ. 2013;6(4):232–8. 23212713 10.1002/ase.1329

[98] Schön DA. The reflective practitioner: How professionals think in action. London, UK: Routledge; 1992. p. 384.

[99] Stanley J, Fellus IA, Rojas D, Talarico S, Radhakrishnan S, Leslie K. Students-as-teachers: Fostering medical educators. Clin Teach. 2022;19(3):235–9. 35174642 10.1111/tct.13471

[100] Stern DT, Cohen JJ, Bruder A, Packer B, Sole A. Teaching humanism. Perspect Biol Med. 2008;51(4):495–507. 18997352 10.1353/pbm.0.0059

[101] Seddon K, Anderson L. Benefits of reflection: a study into the value of small group tutorials to promote reflection in psychiatry students. Health Soc Care Educ. 2012;1(1):5–7.

[102] Siedsma M, Emlet L. Physician burnout: can we make a difference together? Crit Care. 2015;19(1):273. 26134266 10.1186/s13054-015-0990-x PMC4489124

[103] Parsi K, List J. Preparing medical students for the world: Service learning and global health justice. Medscape J Med. 2008;10(11):268. 19099018 PMC2605111

[104] Frankford DM, Patterson MA, Konrad TR. Transforming practice organizations to foster lifelong learning and commitment to medical professionalism. Acad Med. 2000;75(7):708–17. 10926021 10.1097/00001888-200007000-00012

[105] Chou CL, Johnston CB, Singh B, Garber JD, Kaplan E, Lee K, et al. A “safe space” for learning and reflection: One school’s design for continuity with a peer group across clinical clerkships. Acad Med. 2011;86(12):1560–5. 22030757 10.1097/ACM.0b013e31823595fd

[106] Parker S, Leggett A. Teaching the clinical encounter in psychiatry: A trial of balint groups for medical students. Australas Psychiatry. 2012;20(4):343–7. 22767937 10.1177/1039856212447965

[107] Winkel AF, Yingling S, Jones A-A, Nicholson J. Reflection as a learning tool in graduate medical education: a systematic review. J Grad Med Educ. 2017;9(4):430–9. 28824754 10.4300/JGME-D-16-00500.1 PMC5559236

[108] Maxwell TL, Passow ES, Plumb J, Sifri RD. Experience with hospice: Reflections from third-year medical students. J Palliat Med. 2002;5(5):721–7. 12572971 10.1089/109662102320880543

[109] Dhónaill RN, Clarke M, McGarvey A, Joyce P, Holland JC. Physician associate students and their experiences of human cadaveric dissection. J Anat. 2019;234(3):406.

[110] Delany C, Gaunt H. “I left the museum somewhat changed”: Visual arts and health ethics education. Camb Q Healthc Ethics. 2018;27(3):511–24. 29845924 10.1017/S0963180117000913

[111] Byars L, Denton GD. Using art to enhance reflection on professional attributes. J Gen Intern Med. 2012;27:S572.

[112] Artioli G, Deiana L, De Vincenzo F, Raucci M, Amaducci G, Bassi MC, et al. Health professionals and students’ experiences of reflective writing in learning: a qualitative meta-synthesis. BMC Med Educ. 2021;21(1):394. 34294058 10.1186/s12909-021-02831-4 PMC8299581

[113] Cavazos Montemayorr RN, Elizondo-Leal JA, Ramírez Flores YA, Cors Cepeda X, Lopez M. Understanding the dimensions of a strong-professional identity: a study of faculty developers in medical education. Med Educ Online. 2020;25(1):1808369. 32794441 10.1080/10872981.2020.1808369 PMC7482622

[114] Seymour P, Watt M, MacKenzie M, Gallea M. Professional competencies toolkit: Using flash cards to teach reflective practice to medical students in clinical clerkship. MedEdPORTAL. 2018;14:10750. 30800950 10.15766/mep_2374-8265.10750 PMC6342359

[115] Kung JW, Slanetz PJ, Huang GC, Eisenberg RL. Reflective practice: assessing its effectiveness to teach professionalism in a radiology residency. Acad Radiol. 2015;22(10):1280–6. 25863796 10.1016/j.acra.2014.12.025

[116] van Braak M, de Groot E, Veen M, Welink L, Giroldi E. Eliciting tacit knowledge: the potential of a reflective approach to video-stimulated interviewing. Perspect Med Educ. 2018;7(6):386–93. 30446951 10.1007/s40037-018-0487-9 PMC6283779

[117] Quaintance JL, Arnold L, Thompson GS. What students learn about professionalism from faculty stories: an “appreciative inquiry” approach. Acad Med. 2010;85(1):118–23. 20042837 10.1097/ACM.0b013e3181c42acd

[118] O’Sullivan B, Hickson H, Kippen R, Wallace G. Exploring attributes of high-quality clinical supervision in general practice through interviews with peer-recognised GP supervisors. BMC Med Educ. 2021;21(1):441. 34416905 10.1186/s12909-021-02882-7 PMC8376628

[119] O’Loughlin K, Guerandel A, Malone K. A reflection on continuing professional development. Psychiatrist. 2012;36(5):189–93.

[120] Pinto-Powell R, Lahey T. Just a game: The dangers of quantifying medical student professionalism. J Gen Intern Med. 2019;34(8):1641–4. 31147979 10.1007/s11606-019-05063-x PMC6667566

[121] Olive KE, Abercrombie CL. Developing a physician׳s professional identity through medical education. Am J Med Sci. 2017;353(2):101–8. 28183408 10.1016/j.amjms.2016.10.012

[122] Larsen DP, London DA, Emke AR. Using reflection to influence practice: Student perceptions of daily reflection in clinical education. Perspect Med Educ. 2016;5(5):285–91. 27638391 10.1007/s40037-016-0293-1 PMC5035279

[123] Rabow MW, Remen RN, Parmelee DX, Inui TS. Professional formation: extending medicine’s lineage of service into the next century. Acad Med. 2010;85(2):310–7. 20107361 10.1097/ACM.0b013e3181c887f7

[124] Riskin A, Yakov G, Flugelman AA. Group mentoring for junior medical students-the mentor in the reflection cycle. Med Sci Educ. 2021;31(1):137–45. 34457874 10.1007/s40670-020-01146-1 PMC8368910

[125] Wen CC, Lin MJ, Lin CW, Chu SY. Exploratory study of the characteristics of feedback in the reflective dialogue group given to medical students in a clinical clerkship. Med Educ Online. 2015;20:25965. 25661500 10.3402/meo.v20.25965 PMC4320997

[126] Sargeant J, Mann K, van der Vleuten C, Metsemakers J. “Directed” self-assessment: Practice and feedback within a social context. J Contin Educ Health Prof. 2008;28(1):47–54. 18366127 10.1002/chp.155

[127] Brookfield S. Critically reflective practice. J Contin Educ Health Prof. 1998;18(4):197–205.

[128] Fook J. Social work: Critical theory and practice. London, UK: SAGE Publications; 2002. p. 179.

[129] Batty D. Climbié inquiry: The issue explained: The Guardian; 2005 [Available from: https://www.theguardian.com/society/2005/aug/05/climbie.

[130] Kolb D. Experiential learning: Experience as the source of learning and development. 2nd ed. Upper Saddle River, New Jersey: Person Education; 1984. p. 416.

[131] Saucier D, Shaw E, Kerr J, Konkin J, Oandasan I, Organek AJ, et al. Competency-based curriculum for family medicine. Can Fam Physician. 2012;58(6):707–8. 22700736 PMC3374694

[132] Cohn RJ, Plack MM. A cloud with a silver lining: Helping students learn about professionalism. Teach Learn Med. 2017;29(3):304–12. 28632008 10.1080/10401334.2016.1274658

[133] Cruess RL, Cruess SR, Boudreau JD, Snell L, Steinert Y. Reframing medical education to support professional identity formation. Acad Med. 2014;89(11):1446–51. 25054423 10.1097/ACM.0000000000000427

[134] Meyer J, Land R. Threshold concepts and troublesome knowledge (2): Epistemological considerations and a conceptual framework for teaching and learning. High Educ. 2005;49(3):373–88.

[135] Davies P, Mangan J. Threshold concepts and the integration of understanding in economics. Stud High Educ. 2007;32(6):711–26.

[136] Jones H, Hammond L. Threshold concepts in medical education: A scoping review. Med Educ. 2022;56(10):983–93. 35775904 10.1111/medu.14864 PMC9543879

[137] Verweij H, van Ravesteijn H, van Hooff MLM, Lagro-Janssen ALM, Speckens AEM. Does mindfulness training enhance the professional development of residents? A qualitative study. Acad Med. 2018;93(9):1335–40. 29697426 10.1097/ACM.0000000000002260

[138] Pololi L, Clay MC, Lipkin M Jr, Hewson M, Kaplan C, Frankel RM. Reflections on integrating theories of adult education into a medical school faculty development course. Med Teach. 2001;23(3):276–83. 12098399 10.1080/01421590120043053

[139] Vaizer R, Aslam S, Pearson WG Jr, Rockich-Winston N. What does it mean to be a physician? Exploring social imaginaries of first-year medical students. Int J Med Educ. 2020;11:76–80. 32221044 10.5116/ijme.5e30.8f73 PMC7246111

[140] O’Leary K, Farnan J, Didwania A, Anderson A, Wayne D, Humphrey H, et al. Promoting professionalism via a videobased educational workshop for academic hospitalists and housestaff. J Hosp Med. 2012;8(7):386–9. 10.1002/jhm.2056 23780912

[141] Passi V, Johnson S, Peile E, Wright S, Hafferty F, Johnson N. Doctor role modelling in medical education: BEME guide no. 27. Med Teach. 2013;35(9):e1422–1436. 23826717 10.3109/0142159X.2013.806982

[142] Stephenson A, Higgs R, Sugarman J. Teaching professional development in medical schools. Lancet. 2001;357(9259):867–70. 11265967 10.1016/S0140-6736(00)04201-X

[143] Reiser SJ. The ethics of learning and teaching medicine. Acad Med. 1994;69(11):872–6. 7945682 10.1097/00001888-199411000-00002

