# Supplementary material for: A systematic scoping review on group non-written reflections in medical education
Source: BMC Med Educ. 2024 Oct 10;24:1119. doi: 10.1186/s12909-024-06117-3 (PMC11468106; doi:10.1186/s12909-024-06117-3)
Supplement: Supplementary file 1 — Additional file 1. Full SEBA Methodology [file 12909_2024_6117_MOESM1_ESM.docx]

**ADDITIONAL FILE 1. FULL SEBA METHODOLOGY**

**The Systematic Evidenced-based Approach (SEBA)**

To synthesize a coherent narrative from the multiple accounts of GNWR, we adopted Krishna’s Systematic Evidenced-based Approach (SEBA) (1-7). A SEBA-guided Systematic Scoping Review (SEBA-guided SSR) (8-24) that met the PRISMA-ScR criteria (see Additional File 2) facilitated a reproducible, accountable and transparent analysis of patterns, relationships and disagreements from multiple angles (25).

The SEBA process (Figure 1) comprised the following elements: 1) Systematic approach, 2) Split Approach, 3) Jigsaw perspective, 4) Funnelling, 5) Comparing data from evidence-based sources and grey literature, 6) Synthesis of Discussion (1-7, 26-29). Each stage of SEBA was overseen by an expert team that included medical librarians from the Yong Loo Lin School of Medicine (YLLSoM) at the National University of Singapore (NUS), as well as local educational experts and clinicians at the National Cancer Centre Singapore (NCCS), the Palliative Care Institute Liverpool, YLLSoM and Duke-NUS Medical School.

**Active engagement of the expert team throughout the SEBA as** **part of the reiterative process**

**Figure 1.** Stages of the Systematic Evidence-Based Approach (30)

**STAGE 1 of SEBA: The Systematic Approach**

1. Determining the title and background of the review

Ensuring a systematic approach to the synthesis of SSRs in SEBA, the expert team, stakeholders and the research team agreed upon the overall goals of the SSR and the Population, Comparison, and Context (PCC) to be evaluated. The PCC is featured in Table 1.

**Table 1.** PCC, Inclusion Criteria and Exclusion Criteria Applied to Database Search

|  | **Inclusion Criteria** | **Exclusion Criteria** |
| --- | --- | --- |
| **Population** | Doctors in training positions and medical students | Allied health specialties such as dietetics, nursing, psychology, chiropractic, midwifery, social work  Non-medical specialties such as clinical and translational science, veterinary, dentistry |
| **Comparison/ context** | Comparison of accounts and non-written reflective practice (henceforth GNWR) approaches |  |

1. Identifying the research question

Ten members of the research team discussed the research question with medical librarians from the medical library at the Yong Loo Lin School of Medicine at the National University of Singapore (NUS), and local clinicians and educational experts from NCCS, NUS, Assisi Hospice, and Singapore General Hospital.

Our primary research question, *“What is known of GNWR in medical education?”* guided the PCC elements of the inclusion criteria. The secondary research questions were “*How is GNWR structured, assessed and supported in medical education?*” and *“What are the outcomes of GNWR?”.*

Here, the iterative process of the SEBA methodology also led to addition of the following research objectives: *“How is GNWR assessed?”* and *“What barriers and facilitators exist to the applications of GNWR?”*.

1. Inclusion criteria

All grey literature, peer-reviewed articles, narrative reviews, systematic, scoping, and systematic scoping reviews published between 1^st^ January 1990 to 30^th^ June 2023 were included in the PCC (population, concept, and context) inclusion criteria (31, 32). We consider data on medical students and physicians from all levels of training (henceforth clinicians).

1. Searching

Ten members of the research team carried out independent searches from seven bibliographic databases (Pubmed, EMbase, Psychinfo, CINAHL, ERIC, ASSIA, Scopus) and four grey literature databases (Google Scholar, Open Grey, GreyLit, ProQuest) using variations of the terms “non-reflective writing”, “physicians and medical students”, and “medical education” between 15 May 2022 and 15 May 2023. Here, we also considered all accounts of reflective practice and removed accounts of written reflections as a means of enhancing the inclusion of key articles. Table 2 shows an example of the search strategy for PubMed database,

**Table 2**. Example Search Strategy for PubMed database

| **PubMed**  With year filter (from year 1990 to 2021), with language filter (English)  Full strategy: (((("Professionalism"[MeSH] OR "Social Identification"[MeSH] OR "Professional Role"[MeSH] OR "Social Values"[MeSH] OR professional identit*[tiab] OR professionalism*[tiab] OR e-professionalism[tiab] OR identity formation*[tiab] OR socialization[tiab])) AND (("students, medical"[MeSH] OR ((medical[tiab] OR medicine[tiab]) AND (student[tiab] OR students[tiab] OR undergraduate*[tiab] OR postgraduate*[tiab]))) OR ("Physicians"[MeSH] OR "physician"[tiab] OR "Physicians"[tiab] OR "doctor"[tiab] OR "doctors"[tiab] OR "clinician"[tiab] OR "clinicians"[tiab] OR "resident"[tiab] OR "residents"[tiab] OR "residencies"[tiab] OR "residency"[tiab] OR "general practitioner*"[tiab]))) AND ("Thinking"[MeSH] OR "thinking"[tiab] OR "thought"[tiab] OR "thoughts"[tiab] OR "reflect"[tiab] OR "reflects"[tiab] OR "reflection"[tiab] OR "reflections"[tiab] OR "reflective"[tiab])) | |
| --- | --- |
| **Physicians** | "Physicians"[MeSH] OR "physician"[tiab] OR "Physicians"[tiab] OR "doctor"[tiab] OR "doctors"[tiab] OR "clinician"[tiab] OR "clinicians"[tiab] OR "resident"[tiab] OR "residents"[tiab] OR "residencies"[tiab] OR "residency"[tiab] OR "general practitioner*"[tiab] OR |
| **Medical students** | "students, medical"[MeSH] OR (("medical"[tiab] OR "medicine"[tiab]) AND ("student"[tiab] OR "students"[tiab] OR "undergraduate*"[tiab] OR "postgraduate*"[tiab]))) |
| **Reflection** | "Thinking"[MeSH] OR "thinking"[tiab] OR "thought"[tiab] OR "thoughts"[tiab] OR "reflect"[tiab] OR "reflects"[tiab] OR "reflection"[tiab] OR "reflections"[tiab] OR "reflective"[tiab] |
| **Professional identity formation** | ("Professionalism"[Mesh] OR "Social Identification"[Mesh] OR “Profe"trainee"[tiab] OR  "trainees"[tiab]ssional Role”[MeSH] OR "Social Values"[Mesh] OR professional identit*[tiab] OR professionalism*[tiab] OR e-professionalism[tiab] OR identity formation*[tiab] OR socialization[tiab]) |

Each research team member then compiled their own lists of included articles. Through Sandelowski and Barroso (33)’s ‘negotiated consensual validation’ that saw *“research team members articulate, defend, and persuade others of the ‘cogency’ or ‘incisiveness’ of their points of view”*, the shortlisted set of articles was determined and consolidated into a master list for further sieving to ascertain their suitability.

1. Extracting and charting

Using an abstract screening tool, the titles and abstracts were then independently reviewed by members of the research team to further identify relevant articles that met the inclusion criteria outlined in Table 1. Thereafter, full-text articles were filtered by the independent reviewers, facilitating the shortlisting of the included articles. Discussed at online reviewer meetings, the team similarly applied Sandelowski and Barroso (34)’s approach to ‘negotiated consensual validation’ for the deconflicting process to attain consensus on the final list of articles to be included.

1. Assessing quality of articles

The quality of final list of quantitative and qualitative studies included were subsequently appraised by individual members of the research team using the Medical Education Research Study Quality Instrument (MERSQI) (35) and Consolidated Criteria for Reporting Qualitative Studies (COREQ) (36) (see Additional File 3).

**Stage 2 of SEBA: Split Approach**

The Split Approach adopted the simultaneous application of Hsieh and Shannon (37)’s approach to directed content analysis and Braun and Clarke (38)’s thematic analysis. Combined use of these approaches guided the understanding of terminology among various team members, as well as circumvented the limitations of each data analysis method. Content analysis, for instance, was able to account for contradictory data and negative results often excluded within thematic analyses (39-51).

1. Braun and Clarke’s Thematic Analysis

In reviewing the included articles, the first team of researchers adopted Braun and Clarke (52)’s approach to thematic analysis to extract relevant findings. Using the extracted data, the team then formulated a code book categorized in accordance with the emerging themes. Each new emerging code was merged with previous codes in an iterative step-by-step analysis process (53). This led to the synthesis of fresh themes derived from the raw data without prior classification (54). The final list of themes was shortlisted through ‘negotiated consensual validation’ (33) during team meetings.

1. Hsieh and Shannon’s Directed Content Analysis

Simultaneously, the second team of researchers utilized Hsieh and Shannon (55)’s approach to directed content analysis that saw the identification and operationalizing of *a priori coding categories* (55-60). Here, the team drew the codes and categories from Mann, Gordon and MacLeod (61)’s article entitled, *“Reflection and Reflective Practice in Health Professions Education: A Systematic Review”* and Wald and Reis’ (62)*“Beyond the Margins: Reflective Writing and Development of Reflective Capacity in Medical Education”.* As part of the ‘coding agenda’ (63, 64), these pre-existing codes served as a template for coding the included articles. This helped to mitigate concerns on the incoherence, inconsistency, and omission of negative results observed in thematic analysis (3, 42-47, 65, 66). Data uncaptured by the prior codes were also prescribed new codes (63). Similarly, an agreement on the final categories was reached through ‘negotiated consensual validation’ (33, 58).

1. Tabulated Summaries of Included Full-Text Articles

To ensure that the vital facets of the included articles were not lost, the third research team crafted tabulated summaries of the featured articles (see Additional File 3), as guided by Wong, Greenhalgh (25)’s RAMESES publication standards: meta-narrative reviews and Popay, Roberts (67)’s *“Guidance on the conduct of narrative synthesis in systematic reviews”.*

**Stage 3 of SEBA: The Jigsaw Perspective**

The Jigsaw Perspective (68, 69) was guided by Phases 4 to 6 of France et al. (70)’s adaptation of Noblit et al. (71)’s seven phases of meta-ethnographic approach. Reimagined as pieces of a jigsaw puzzle, this stage entailed the merging of complementary/overlapping themes and categories from the thematic and content analyses to create broader ‘themes/categories’ that formed the puzzle. To do so, the themes and subthemes were first compared with the categories and subcategories identified. Similarities were verified by comparing the codes contained within them. A summary of the extracted data that formed the themes/categories is enclosed in Additional File 4.

**Stage 4 of SEBA: The Funnelling Process**

The Funnelling Process encompassed the comparison of the resulting ‘themes/categories’ with that of the tabulated summaries, as well quality appraisals using MERSQI and COREQ (35, 36). This led to the creation of domains that outlined the basis of the ensuing discussion in Stage 6 of SEBA.

**The iterative process within SEBA**

A pivotal feature of the SEBA process is its iterative process. Identification of PIF-related facets called for the contextualisation of these features and the introduction of relevant lenses within the discussion section.

**Stage 5 of SEBA: Analysis of Evidence-based and Non-data driven Literature**

Concerns regarding the plausibility of data from grey literature—which were neither peer-reviewed nor necessarily evidence-based—biasing the synthesis of the discussion led to the research team thematically analysing reflective and opinion papers, editorials, commentaries, letters, forum discussions, blogs, governmental reports, policy statements and surveys included from the academic databases. The emerging themes from the non-data driven literature were then compared with themes from research-based, peer-reviewed data. With the themes from both types of data sources found to be similar, it was unlikely that data from grey literature would have influenced the analysis.

**Stage 6 of SEBA: Synthesis of Scoping Review in SEBA**

The Best Evidence Medical Education (BEME) Collaboration Guide and the Structured approach to the Reporting In healthcare education of Evidence Synthesis (STORIES) were used to guide the discussion.

**REFERENCES**

1. Chua KZY, Quah ELY, Lim YX, Goh CK, Lim J, Wan DWJ, et al. A systematic scoping review on patients’ perceptions of dignity. BMC Palliative Care. 2022;21(1):118.

2. Ong YT, Quek CWN, Pisupati A, Loh EKY, Venktaramana V, Chiam M, et al. Mentoring future mentors in undergraduate medical education. PLOS ONE. 2022;17(9):e0273358.

3. Chan NPX, Chia JL, Ho CY, Ngiam LXL, Kuek JTY, Ahmad Kamal NHB, et al. Extending the Ring Theory of Personhood to the Care of Dying Patients in Intensive Care Units. Asian Bioethics Review. 2022;14(1):71-86.

4. Kuek JTY, Ngiam LXL, Kamal NHA, Chia JL, Chan NPX, Abdurrahman ABHM, et al. The impact of caring for dying patients in intensive care units on a physician’s personhood: a systematic scoping review. Philosophy, Ethics, and Humanities in Medicine. 2020;15(1):12.

5. Ngiam L, Ong, Y. T., Ng, J. X., Kuek, J., Chia, J. L., Chan, N., Ho, C. Y., Abdurrahman, A., Kamal, N., Cheong, C., Ng, C. H., Tan, X. H., Tan, L., Chin, A., Mason, S., Jumat, M. R., Chiam, M., & Krishna, L. Impact of Caring for Terminally Ill Children on Physicians: A Systematic Scoping Review. The American journal of hospice & palliative care. 2021;38(4):396–418.

6. Ho CY, Lim N-A, Ong YT, Lee ASI, Chiam M, Gek GPL, et al. The impact of death and dying on the personhood of senior nurses at the National Cancer Centre Singapore (NCCS): a qualitative study. BMC palliative care. 2022;21(1):1-10.

7. Goh S, Wong RSM, Quah ELY, Chua KZY, Lim WQ, Ng ADR, et al. Mentoring in palliative medicine in the time of covid-19: a systematic scoping review. BMC Medical Education. 2022;22(1):359.

8. Teo YH PT, Abdurrahman A, Lee ASI, Chiam M, Fong W, et al. A modified Delphi approach to enhance nurturing of professionalism in postgraduate medical education in Singapore. Singapore Med J. 2021.

9. Venktaramana V LE, Wong CJW, Yeo JW, Teo AYT, Chiam CSY, et al. . A systematic scoping review of communication skills training in medical schools between 2000 and 2020. Med Teach. 2022;44:997-1006.

10. Goh S WR, Quah ELY, Chua KZY, Lim WQ, Ng ADR, et al. Mentoring in palliative medicine in the time of covid-19: a systematic scoping review. BMC Med Educ. 2022;22.

11. Cheong CWS QE, Chua KZY, Lim WQ, Toh RQE, Chiang CLL, et al. Post graduate remediation programs in medicine: a scoping review. . BMC Med Educ. 2022;22(294).

12. Chan NPX CJ, Ho CY, Ngiam LXL, Kuek JTY, Ahmad Kamal NHB, et al. Extending the Ring Theory of Personhood to the Care of Dying Patients in Intensive Care Units. Asian Bioeth Rev. 2021;14:71-86.

13. Vig PS L, J.Y., Lee, R.W., Huang, H., Tan, X.H., Lim, W.Q., Lim, M.B., Lee, A.S., Chiam, M., Lim, C., Baral, V.R. Parental bereavement–impact of death of neonates and children under 12 years on personhood of parents: a systematic scoping review. BMC Palliative Care. 2021;20(136).

14. Huang H TR, Chiang CLL, Thenpandiyan AA, Vig PS, Lee RWL, et al. Impact of Dying Neonates on Doctors' and Nurses' Personhood: A Systematic Scoping Review. J Pain Symptom Manage. 2022;63:e59-e74.

15. Chia EW H, H., Goh, S., Peries, M.T., Lee, C.C., Tan, L.H., Khoo, M.S., Tay, K.T., Ong, Y.T., Lim, W.Q., Tan, X.H. A systematic scoping review of teaching and evaluating communications in the intensive care unit. Asia Pac Schol. 2021;6:3-29.

16. Zhou JX GC, Chiam M, Krishna LKR. Painting and Poetry From a Bereaved Family and the Caring Physician. Journal of Pain and Symptom Management. 2023;65(5):e503-e6.

17. Chiam M H, C.Y., Quah, E. et al. . Changing self-concept in the time of COVID-19: a close look at physician reflections on social media. Philosophy, Ethics and Humanities in Medicine. 2022;17(1).

18. Tay J CS, Phua G, Zhuang Q, Neo S, Lee G, et al. Perceptions of healthcare professionals towards palliative care in internal medicine wards: a cross-sectional survey. BMC Palliative Care. 2021;20(101):1-8.

19. Chua KZY, Quah, E.L.Y., Lim, Y.X. et al. A systematic scoping review on patients’ perceptions of dignity. BMC Palliative Care. 2022;21.

20. Toh RQE, Koh, K.K., Lua, J.K. et al. The role of mentoring, supervision, coaching, teaching and instruction on professional identity formation: a systematic scoping review. BMC Med Educ. 2022;22.

21. Ho CY, Lim, NA., Ong, Y.T. et al. The impact of death and dying on the personhood of senior nurses at the National Cancer Centre Singapore (NCCS): a qualitative study. BMC Palliative Care. 2022;21(83).

22. Ong YT QC, Pisupati A, Loh EKY, Venktaramana V, Chiam M, et al. Mentoring future mentors in undergraduate medical education. PLoS One. 2022;17.

23. Quah ELY CK, Lua JK, Wan DWJ, Chong CS, Lim YX, Krishna L. A Systematic Review of Stakeholder Perspectives of Dignity and Assisted Dying. J Pain Symptom Manage. 2022;65:e123-e36.

24. Quek CWN OR, Wong RSM, et al. Systematic Scoping Review on Moral Distress among Physicians. BMJ Open. 2022;12.

25. Wong G, Greenhalgh T, Westhorp G, Buckingham J, Pawson R. RAMESES publication standards: meta-narrative reviews. BMC medicine. 2013;11(1):20.

26. Pring R. The ‘false dualism’of educational research. Journal of Philosophy of Education. 2000;34(2):247-60.

27. Crotty M. The Foundations of Social Research: Meaning and Perspective in the Research Process: SAGE; 1998.

28. Ford K. Taking a Narrative Turn: Possibilities, Challenges and Potential Outcomes. OnCUE Journal. 2012.

29. Schick-Makaroff K, MacDonald M, Plummer M, Burgess J, Neander W. What Synthesis Methodology Should I Use? A Review and Analysis of Approaches to Research Synthesis. AIMS Public Health. 2016;3:172-215.

30. Lim JY, Ong SYK, Ng CYH, Chan KLE, Wu SYEA, So WZ, et al. A systematic scoping review of reflective writing in medical education. BMC Medical Education. 2023;23(1):12.

31. Peters M, Godfrey C, McInerney P, Soares C, Khalil H, Parker D. The Joanna Briggs Institute reviewers' manual 2015: methodology for JBI scoping reviews2015 April 29, 2019. Available from: <http://joannabriggs.org/assets/docs/sumari/Reviewers-Manual_Methodology-for-JBI-Scoping-Reviews_2015_v1.pdf>.

32. Peters MD, Godfrey CM, Khalil H, McInerney P, Parker D, Soares CB. Guidance for conducting systematic scoping reviews. Int J Evid Based Healthc. 2015;13(3):141-6.

33. Sandelowski M, Barroso J. Handbook for synthesizing qualitative research: Springer Publishing Company; 2006.

34. Sandelowski M BJ. Handbook for synthesizing qualitative research. New York: Springer; 2007.

35. Reed DA, Beckman TJ, Wright SM, Levine RB, Kern DE, Cook DA. Predictive validity evidence for medical education research study quality instrument scores: quality of submissions to JGIM's Medical Education Special Issue. J Gen Intern Med. 2008;23(7):903-7.

36. Tong A, Sainsbury P, Craig J. Consolidated criteria for reporting qualitative research (COREQ): a 32-item checklist for interviews and focus groups. International journal for quality in health care. 2007;19(6):349-57.

37. Hsieh HF, Shannon SE. Three approaches to qualitative content analysis. Qual Health Res. 2005;15(9):1277-88.

38. Braun V, Clarke V. Using thematic analysis in psychology. . Qualitative Research in Psychology 2006;3(2):77-101.

39. Mah ZH, Wong RSM, Seow REW, Loh EKY, Kamal NHA, Ong RRS, et al. A Systematic Scoping Review of Systematic Reviews in Palliative Medicine Education. Palliative Medicine & Care: Open Access. 2020;7(1):1-12.

40. Ong RRS, Seow REW, Wong RSM, Loh EKY, Kamal NHA, Mah ZH, et al. A Systematic Scoping Review of Narrative Reviews in Palliative Medicine Education. Palliative Medicine & Care: Open Access. 2020;7(1):1-22.

41. Kamal NHA, Tan LHE, Wong RSM, Ong RRS, Seow REW, Loh EKY, et al. Enhancing education in Palliative Medicine: the role of Systematic Scoping Reviews. Palliative Medicine & Care: Open Access. 2020;7(1):1-11.

42. Zhou YC, Tan SR, Tan CGH, Ng MSP, Lim KH, Tan LHE, et al. A systematic scoping review of approaches to teaching and assessing empathy in medicine. BMC Med Educ. 2021;21(1):1-15.

43. Ong ZH, Tan LH, Ghazali HZ, Ong YT, Koh JW, Ang RZ, et al. A systematic scoping review on pedagogical strategies of interprofessional communication for physicians in emergency medicine. Journal of medical education and curricular development. 2021;8:23821205211041794.

44. Tan XH, Foo MA, Lim SLH, Lim MBXY, Chin AMC, Zhou J, et al. Teaching and assessing communication skills in the postgraduate medical setting: a systematic scoping review. BMC Med Educ. 2021;21(1):1-19.

45. Wong MK, Hong DZH, Wu J, Ting JJQ, Goh JL, Ong ZY, et al. A systematic scoping review of undergraduate medical ethics education programs from 1990 to 2020. Medical Teacher. 2022;44(2):167-86.

46. Nowell LS, Norris JM, White DE, Moules NJ. Thematic analysis: Striving to meet the trustworthiness criteria. International journal of qualitative methods. 2017;16(1):1609406917733847.

47. Teo YH, Peh TY, Abdurrahman A, Lee ASI, Chiam M, Fong W, et al. A modified Delphi approach to enhance nurturing of professionalism in postgraduate medical education in Singapore. Singapore Med J. 2021.

48. Vig PS, Lim JY, Lee RWL, Huang H, Tan XH, Lim WQ, et al. Parental bereavement - impact of death of neonates and children under 12 years on personhood of parents: a systematic scoping review. BMC Palliat Care. 2021;20(1):136.

49. Sarraf-Yazdi S, Teo YN, How AEH, Teo YH, Goh S, Kow CS, et al. A Scoping Review of Professional Identity Formation in Undergraduate Medical Education. J Gen Intern Med. 2021;36(11):3511-21.

50. Chan NPX, Chia JL, Ho CY, Ngiam LXL, Kuek JTY, Ahmad Kamal NHB, et al. Extending the Ring Theory of Personhood to the Care of Dying Patients in Intensive Care Units. Asian Bioeth Rev. 2021;14(1):1-16.

51. Ng YX, Koh ZYK, Yap HW, Tay KT, Tan XH, Ong YT, et al. Assessing mentoring: A scoping review of mentoring assessment tools in internal medicine between 1990 and 2019. PloS one. 2020;15(5):e0232511.

52. Braun V, Clarke V. Using thematic analysis in psychology. Qualitative Research in Psychology. 2006;3(2):77-101.

53. Voloch K-A, Judd N, Sakamoto K. An innovative mentoring program for Imi Ho'ola Post-Baccalaureate students at the University of Hawai'i John A. Burns School of Medicine. Hawai'i Medical Journal. 2007;66(4):102.

54. Cassol H, Pétré B, Degrange S, Martial C, Charland-Verville V, Lallier F, et al. Qualitative thematic analysis of the phenomenology of near-death experiences. PLOS One. 2018;13(2):e0193001.

55. Hsieh H-F, Shannon SE. Three Approaches to Qualitative Content Analysis. Qualitative Health Research. 2005;15(9):1277-88.

56. Neal JW, Neal ZP, Lawlor JA, Mills KJ, McAlindon K. What makes research useful for public school educators? Administration Policy in Mental Health Mental Health Services Research

2018;45(3):432-46.

57. Wagner-Menghin M, de Bruin A, van Merriënboer JJ. Monitoring communication with patients: analyzing judgments of satisfaction (JOS). Advances in Health Sciences Education. 2016;21(3):523-40.

58. Elo S, Kyngäs H. The qualitative content analysis process. Journal Of Advanced Nursing. 2008;62(1):107-15.

59. Mayring P. Qualitative content analysis. A Companion To Qualitative Research. 2004;1:159-76.

60. Humble ÁM. Technique triangulation for validation in directed content analysis. International Journal of Qualitative Methods. 2009;8(3):34-51.

61. Mann K, Gordon J, MacLeod A. Reflection and reflective practice in health professions education: A systematic review. Adv in Health Sci Educ. 2009;14(4):595-621.

62. Wald HS, Reis SP. Beyond the margins: reflective writing and development of reflective capacity in medical education. Journal of general internal medicine. 2010;25(7):746-9.

63. Mayring P. Qualitative content analysis. Forum Qualitative Sozialforschung / Forum: Qualitative Social Research. 2004;1(2).

64. Kibiswa NK. Directed qualitative content analysis (DQlCA): A tool for conflict analysis. The Qualitative Report. 2019;24(8):2059-79.

65. Vig PS, Lim JY, Lee RWL, Huang H, Tan XH, Lim WQ, et al. Parental bereavement - impact of death of neonates and children under 12 years on personhood of parents: a systematic scoping review. BMC Palliative Care. 2021;20(1):136.

66. Sarraf-Yazdi S, Teo YN, How AEH, Teo YH, Goh S, Kow CS, et al. A Scoping Review of Professional Identity Formation in Undergraduate Medical Education. J Gen Intern Med. 2021;36(11):3511-21.

67. Popay J, Roberts H, Sowden A, Petticrew M, Arai L, Rodgers M, et al. Guidance on the conduct of narrative synthesis in systematic reviews. A product from the ESRC methods programme Version. 2006;1:b92.

68. France EF, Wells M, Lang H, Williams B. Why, when and how to update a meta-ethnography qualitative synthesis. Syst Rev. 2016;5:44.

69. Noblit GW HR. Meta-ethnography : synthesizing qualitative studies. Newbury Park: Sage Publications; 1988.

70. France EF, Wells M, Lang H, Williams B. Why, when and how to update a meta-ethnography qualitative synthesis. Systematic Reviews. 2016;5(1):44.

71. Noblit GW, Hare RD. Meta-ethnography : synthesizing qualitative studies. Newbury Park, Calif.: Sage Publications; 1988.
