# Supplementary material for: A systematic scoping review on group non-written reflections in medical education
Source: BMC Med Educ. 2024 Oct 10;24:1119. doi: 10.1186/s12909-024-06117-3 (PMC11468106; doi:10.1186/s12909-024-06117-3)
Supplement: Supplementary file 3 — Additional file 3. Tabulated Summaries of Included Full-Text Articles [file 12909_2024_6117_MOESM3_ESM.docx]

**Additional File 3: Tabulated Summaries of Included Full-Text Articles**

| **Authors/Year** | **Article Title** | **Type of Study** | **Study Aim** | **Methodology** | **Key Findings** | **Conclusions** | **MERSQI** | **COREQ** |
| --- | --- | --- | --- | --- | --- | --- | --- | --- |
| Alizadeh Et Al. 2017 | Leadership Identity Development Through Reflection and Feedback in Team-Based Learning Medical Student Teams | Quantitative | Studies on leadership identity development through reflection with Team-Based Learning (TBL) in medical student education are rare. We assumed that reflection and feedback on the team leadership process would advance the progression through leadership identity development stages in medical students within the context of classes using | This study is a quasi experimental design with pretest–posttest control group. The pretest and posttest were reflection papers of medical students about their experience of leadership during their TBL sessions. In the intervention group, TBL and a team-based, guided reflection and feedback on the team leadership process were performed at the end of all TBL sessions. In the other group, only TBL was used. The Stata 12 software was used. Leadership Identity was treated both as a categorical and quantitative variable to control for differences in baseline and gender variables. Chi-square, t tests, and linear regression analysis were performed. Context: The population was a cohort of 2015–2016 medical students in a TBL setting at Tehran University of Medical Sciences, School of Medicine. Teams of four to seven students were formed by random sorting at the beginning of the academic year (intervention group n D 20 teams, control group n D 19 teams). | At baseline, most students in both groups were categorized in the Awareness and Exploration stage of leadership identity: 51 (52%) in the intervention group and 59 (55%) in the control group: uncorrected x2(3) D 15.6, design based F(2.83, 108) D 4.87, p D .003. In the posttest intervention group, 36 (36%) were in exploration, 33 (33%) were in L-identified, 20 (20%) were in Leadership Differentiated, and 10 (10%) were in the Generativity. None were in the Awareness or Integration stages. In the control group, 3 (20%) were in Awareness, 56 (53%) were in Exploration, 35 (33%) were in Leader Identified, 13 (12%) were in Leadership Differentiated. None were in the Generativity and Integration stages. Our hypothesis was supported by the data: uncorrected x2(4) D 18.6, design-based F(3.77, 143) D 4.46, p D .002. The mean of the leadership identity in the pretest, intervention group equaled 1.93 (SD D 0.85) and the pretest, control group mean was 2.36 (SD D 0.86), p D .004. The mean of the posttest, intervention group was 3.04 (SD D 0.98) and posttest, control group mean was 2.54 (SD D 0.74), TD¡4.00, design dfD 38, p < .001, and adjusted on baseline and gender TD¡8.97, design dfD 38, p < .001 | Reflection and feedback on the team leadership process in TBL advances the progression in stages of leadership identity development in medical students. Although the TBL strategy itself could have an impact on leadership identity development, this study demonstrates that when a reflection and feedback on leadership intervention are added, there is much greater impact. | 15 | NA |
| C. J. Allison and G. P. Pullen, 1981 | Student discussion groups on doctor-patient relationships: a critical assessment | Qualitative | The main aim was to prevent the alleged dehumanizing effect of medical education. The groups were monitored by attendance records, questionnaires and tape-recordings. | Regular discussion groups for all students were introduced at a new clinical medical school to enable students to discuss freely their responses to patients. | It was concluded that, while organizational  details and inappropriate styles of conducting the groups were partly responsible, the main problem was the apparent conflict in attitudes between the students medical teachers and their group conductors (who were mainly psychiatrists), i.e. between the concepts of modem technological medicine and those of psycho-social medicine. This induced a conflict of motivation in the students, who naturally need to identify with their teachers, which most of them resolved by avoiding the groups | It is suggested that more effective integration between general medical teachers and the organizers of such groups is needed, and that group conductors should include general practitioners and clinical supervisors as well as psychiatrists | NA | 17 |
| Artioli Et Al. 2021 | Health professionals and students’ experiences of reflective writing in learning: A qualitative meta-synthesis | Qualitative | Reflective writing provides an opportunity for health professionals and students to learn from their mistakes, successes, anxieties, and worries that otherwise would remain disjointed and worthless. This systematic review addresses the following question: “What are the experiences of health professionals and students in applying reflective writing during their education and training?” | We performed a systematic review and meta-synthesis of qualitative studies. Our search comprised six electronic databases: MedLine, Embase, Cinahl, PsycINFO, Eric, and Scopus. Our initial search produced 1237 titles, excluding duplicates that we removed. After title and abstract screening, 17 articles met the inclusion criteria. We identified descriptive themes and the conceptual elements explaining the health professionals’ and students’ experience using reflective writing during their academic and in-service training by performing a meta-synthesis. | We identified four main categories (and related sub-categories) through the meta-synthesis: reflection and reflexivity, accomplishing learning potential, building a philosophical and empathic approach, and identifying reflective writing feasibility. We placed the main categories into an interpretative model which explains the users’ experiences of reflective writing during their education and training. Reflective writing triggered reflection and reflexivity that allows, on the one hand, skills development, professional growth, and the ability to act on change; on the other hand, the acquisition of empathic attitudes and sensitivity towards one’s own and others’ emotions. Perceived barriers and impeding factors and facilitating ones, like timing and strategies for using reflective writing, were also identified. | The use of this learning methodology is crucial today because of the recognition of the increasing complexity of healthcare contexts requiring professionals to learn advanced skills beyond their clinical ones. Implementing reflective writing-based courses and training in university curricula and clinical contexts can benefit human and professional development. | NA | NA |
| Bennett Et Al. 2011 | CanMEDS reflect? Mapping student reflections on professionalism to a competency framework | Qualitative | Reflection is core to professional development.1 In practice students and clinicians struggle with perceived vagueness of reflective practice.2 A number of methods are promoted to develop reflective practice.3-5 We introduced the use of parallel charting, a pedagogical tool to provide space and time for reflection and discussion with peers on professional issues.6 The parallel chart is a reflection on some aspect of patient care which does not belong in the traditional medical record. We were interested to discover what issues students would reflect on and how these would relate to models of professionalism. CanMEDS is the competency framework adopted within our university. We decided to investigate whether student reflections on professionalism map to The CanMEDS Physician Competency Framework. | Year 3 students (n = 108) wrote a parallel chart during each of 3 clinical attachments. The charts were transcribed and imported into NVivo9. Two independent coders (MK, DB) performed an initial round of descriptive coding. The CanMEDS framework was used to define pre-ordinate themes to categorise the codes, however responsive categorisation to themes outside the framework was also included within the study design. | Students reflected on a range of topics which map well to CanMEDS. The CanMEDS roles of Communicator and Professional predominated. Collaborator, Medical Expert and Health Advocate roles were less frequently reflected upon. Manager was rarely mentioned. No emergent categories were identified. Examples of student reflections will be presented. | Junior student learning about medical professionalism in the clinical environment is focussed on the elements which are more visible in practice. Other doctor roles, are either less apparent or need to be actively demonstrated. Ringsted et al.8 found that doctors rated the Communicator role the most important, with the Health Advocate role rated least important. Student reflections were consistent with this emphasis. We suggest that the use of a competency framework such as CanMEDs may use a useful strategy to direct reflective practice and help students reflect on the full scope of professional roles. | NA | 10 |
| William T. Branch, Jr. and Maura George  2017 | Reflection-Based Learning for Professional Ethical Formation | Commentary | One way practitioners learn ethics is by reflecting on experience. They may reflect in the moment (reflection-in-action) or afterwards (reflection-on-action). | We illustrate how a teaching clinician may transform relationships with patients and teach person-centered care through reflective learning. We discuss reflective learning pedagogies and present two case examples of our preferred method, guided group reflection using narratives. | This method fosters moral development alongside professional identity formation in students and advanced learners. | Our method for reflective learning addresses and enables processing of the most pressing ethical issues that learners encounter in practice. | NA | NA |
| William T. Branch, Jr.  2010 | The road to professionalism: Reflective practice and reflective learning | Qualitative | This paper describes educational programs designed to create humanistic physicians who are skilled in communicating with patients and committed to professional values. The educational programs addressed these goals in medical students, residents and young faculty members over a 22-year period. Evaluations enhanced understanding and documented outcomes of the programs. | Detailed descriptions of the design and educational methods employed by the programs are given, along with results of their evaluations. | Key features of the educational programs that effectively influenced learners’ skills, values, attitudes, and behaviors include: (a) longitudinal learning in small groups, (b) creating a supportive group process, (c) prominent inclusion of reflective learning, and (d) experiential learning of skills. Qualitative analyses provided an understanding of the learning processes and the transformative nature of the programs. Surveys and questionnaires documented statistically significant outcomes achieving the programs’ goals. | Longitudinal educational programs employing critical reflection alongside mastery of skills enhance humanistic values and may have transformative effects on their learners. Practical implication: The descriptions and outcomes described herein for medical students, residents and young faculty members may suggest blueprints for future educational efforts aimed at producing humanistic professionals. | NA | 19 |
| Al. 2012 | A Curricular Addition Using Art to Enhance Reflection on Professional Values | Mixed Methods | Art and humanities can enhance undergraduate medical education curricular objectives. Most commonly, art is used to help students learn observational skills, such as medical interviewing and physical diagnosis. Educators concurrently struggle to find ways to meaningfully teach professional values within crowded curricula. | This curriculum aimed to combine art and reflection to actively convey tenets of medical professionalism. Setting: Internal medicine clerkship at a single institution. Participants: Third-year students. Program description: Students reviewed an online module describing attributes of medical professionalism before completing a 4-step written exercise stimulated by viewing a work of art and based on a critical incident from their own experiences. A faculty member reviewed the essays and facilitated small group discussion to normalize the students’ emotional responses and generalize their observations to others. | The curriculum was acceptable to students and enthusiastically received by faculty. Efforts to assess the effects and durability of the exercise on student behavior are ongoing. | Artwork can enhance student reflection on professional values. This model efficiently and creatively meets curricular professionalism objectives. | 7 | 12 |
| Chambers Et Al. 2012 | Introducing medical students to reflective practice | Qualitative | Reflective practice is an important skill in clinical practice and is being introduced to medical students early in their undergraduate education. | It is important for GPs involved in teaching and supervising undergraduate medical students to have an understanding of how reflective practice is being taught in undergraduate curricula and how medical students evaluate it.  This paper summarises the literature published around introducing medical students to reflective practice, including its various definitions, students’ attitudes towards it, and the debates over what form it should take, how it should be assessed, and when it should be introduced. | A variety of approaches are currently being used to stimulate medical student reflection. Current research suggests that high-quality guidance and feedback are key to enabling students to develop a positive attitude and understanding of reflection | Further research is needed on when to introduce reflective practice to medical students, how best to nurture reflection, and how best to assess it. | NA | NA |
| Choo Et Al. 2020 | The informal curriculum: what do junior doctors learn from a palliative care rotation? | Qualitative | Junior doctors learn from the formal and informal curriculum. In a palliative care rotation, the informal curriculum may be useful in teaching attitudes like empathy and compassion. Our study aims to explore how the informal curriculum augments the formal curriculum of a palliative care rotation in shaping the professional development of a doctor. | We conducted a qualitative study with seven focus group discussions involving 21 junior doctors (medical officers and residents) who spent at least 2 months in a palliative care setting in a tertiary hospital or an inpatient hospice. Data were analysed using qualitative thematic analysis to identify the themes related to the junior doctors' perceptions of how the informal curriculum impacted their humanistic and professional development, thereby augmenting the formal curriculum in a palliative care setting. | Three main themes illustrated how the informal curriculum influenced the doctors: (1) reconceptualisation of control: shifting perspectives as they grappled with their envisioned control versus reality while caring for dying patients; (2) emergence of professionalism: adapting perspectives as they learnt how to bridge theory and reality while developing professionalism and (3) personal growth: forming new perspectives, as doctors reflected on life, death and their calling through a renewed lens. | This study explored how the informal curriculum influenced doctors’ perceptions about professionalism and personal growth, embodying the values of the profession. Observations and interpersonal interactions with healthcare professionals, patients and their caregivers encouraged the doctors to reflect upon their own calling into medicine. | NA | 21 |
| Chou Et Al. 2011 | A “Safe Space” for Learning and Reflection: One School’s Design for Continuity With a Peer Group Across Clinical Clerkships | Qualitative | The value of continuity in medical education, particularly during clerkships, is increasingly recognized. Previous clerkship-based models have described changes that emphasize continuity in patient care, learner supervision, and curriculum. The creation of continuous student peer groups can foster interactions that enhance mutual support through uncomfortable professional transitions during the clerkship years. | Here, the authors describe a third-year clerkship model based at the San Francisco Veterans Affairs (VA) Medical Center called VA Longitudinal Rotations (VALOR), designed explicitly to establish a supportive learning environment for small peer groups. Seven groups of medical students (42 total) completed VALOR across three academic years between 2007 and 2009. On clerkships during VALOR, one hour per week was designated for faculty-facilitated sessions amongst peer groups. Students’ perceptions of peer group support and overall program satisfaction were determined with immediate post surveys and focus groups at the end of VALOR, and with follow-up surveys 5 to 27 months after completing VALOR. | Students strongly valued several elements of VALOR peer groups, including support through clerkship challenges, meeting for facilitated reflection, and appreciating patient experiences across the continuum of care. Students’ appreciation for their peer group experiences persisted well after the conclusion of VALOR. VALOR students performed the same as or better than traditional clerkship students on knowledge and skill-based outcomes. | The authors demonstrate that their third year clerkship program using peer groups has built supportive learning networks and facilitated reflection, allowing students to develop critical professional skills. Student communication around patient care was also feasible and highly valued. | NA | 18 |
| Chua Et Al. 2019 | The Patient Experience Debrief Interview: How Conversations With Hospitalized Families Influence Medical Student Learning and Reflection | Mixed methods | To determine the effect of patient debrief interviews on pediatric clerkship student depth of reflection and learning. | The authors conducted a multi institutional, mixed-methods, cluster randomized trial among pediatric clerkship students from May 2016 to February 2017. Intervention students completed a debrief interview with a patient–caregiver, followed by a written reflection on the experience. Control students completed a written reflection on a memorable patient encounter. Three blinded authors scored written reflections according to the 4-level REFLECT rubric to determine depth of reflection. Interrater reliability was examined using kappa. REFLECT scores were analyzed using a chi-square test; essays were analyzed using content analysis. | Eighty percent of eligible students participated. One hundred eighty-nine essays (89 control, 100 intervention) were scored. Thirty-seven percent of the control group attained reflection and critical reflection, the 2 highest levels of reflection, compared with 71% of the intervention group; 2% of the control group attained critical reflection, the highest level, compared with 31% of the intervention group (χ2  (3, N = 189) = 33.9,  P < .001). Seven themes were seen across both groups, 3 focused on physician practice and 4 focused on patients. Patient-centered themes were more common in the intervention group, whereas physician-focused themes were more common in the control group. | Patient debrief interviews offer a unique approach to deepen self reflection through direct dialogue and exploration of patient–caregiver experiences during hospitalization. | 15.5 | 20 |
| Daryazadeh Et Al. 2020 | Impact of a narrative medicine program on reflective capacity and empathy of medical students in Iran | Quantitative | Narrative medicine consists of the expression of medical experiences and the reflection on narratives to foster empathic communication with patients. Reflecting on narratives increases self-awareness and recognition of the feelings of the narrator or the story’s main character, which in turn affects the audience. This study was conducted to examine the impact of a narrative medicine program on the reflective capacity and empathy of medical students. | A quasi-experimental study was performed during the 2018–2019 academic year at Isfahan University of Medical Sciences in Iran involving 135 medical interns in 2 groups (control [n=66] and experimental [n=69]). Interns in the experimental group took part in seven 2-hour reflective practice sessions, while those in the control group underwent no educational intervention. Pre-test and post-test assessments were conducted for both groups using 2 valid and reliable tools for the assessment of reflective capacity and empathy. Mean reflection and empathy scores were compared within groups (between pre- and post-test values) and between groups (using the paired-t test and the t-test; P≤0.05). | The mean reflection and empathy scores of the experimental group significantly increased from pre-test to post-test, but those of the control group did not. Moreover, the mean post-test scores were significantly different between the 2 groups (P<0.001). | Narrative medicine is an effective teaching method that can improve reflective capacity and empathy, thereby ultimately promoting professionalism as a core competency in medicine. Consideration of learning conditions and interdisciplinary teaching are necessary for implementing a narrative medicine program. | 13 | NA |
| Dijkhuizen Et Al.  2018 | Encouraging residents’ professional development and career planning: the role of a development-oriented performance assessment | Qualitative | Current postgraduate medical training programmes fall short regarding residents’ development of generic competencies (communication, collaboration, leadership, professionalism) and reflective and deliberate practice. Paying attention to these non-technical skills in a structural manner during postgraduate training could result in a workforce better prepared for practice. A development-oriented performance assessment (PA), which assists residents with assessment of performance and deliberately planned learning activities, could potentially contribute to filling this gap. This study aims to explore residents experiences with the PA. | We conducted a qualitative interview study with 16 residents from four different medical specialties who participated in the PA, scheduled halfway postgraduate training. The PA was conducted by an external facilitator, a psychologist, and focused specifically on professional development and career planning. Residents were interviewed 6 months after the PA. Data were analysed using the framework method for qualitative analysis. | Residents found the PA to be of additional value for their training. The overarching merit was the opportunity to evaluate competencies not usually addressed in workplace-based assessments and progress conversations. In addition, the PA proved a valuable tool for assisting residents with reflecting upon their work and formulating their learning objectives and activities. Residents reported increased awareness of capacity, selfconfidence and enhanced feelings of career-ownership. An important factor contributing to these outcomes was the relationship of trust with the facilitator and programme director. | The PA is a promising tool in fostering the development of generic competencies and reflective and deliberate practice. The participating residents, facilitator and programme directors were able to contribute to a safe learning environment away from the busy workplace. The facilitator plays an important role by providing credible and informative feedback. Commitment of the programme director is important for the implementation of developmental plans and learning activities. | NA | 24 |
| Dixon Et Al. 2021 | A Brief Coaching Pilot Enhances Professional Identity Formation and Clinical Skills Acquisition During Emergency Medicine Clerkships Shortened by COVID-19 | Qualitative | The Covid-19 pandemic limited educational and career development opportunities for medical students, requiring innovative programs to accelerate professional identity formation and clinical skills acquisition. | We developed a brief coaching intervention that took place over the advanced (subinternship) emergency medicine rotation at our institution. We trained coaches using a newly developed workshop, who met with students for an average of 4.5 hours over 3 weeks. | We showed that this coaching program was both feasible and impactful for faculty coaches and medical students. | representation of different subspecialties within the field of EM. Our just-in-time coach training workshop and materials helped position our program to have this impact despite none of our junior faculty coaches having prior coaching experience. Our subjects found the intervention acceptable and impactful. There is significant potential for replication in other specialties or other EM clerkships. | 8 | 11 |
| Goldie Et Al. 2007 | Teaching professionalism in the early years of a medical curriculum: a qualitative study | Qualitative | Despite the growing literature on professionalism in undergraduate medical curricula, few studies have examined its delivery. This study investigated tutors’ and students’ perspectives of the delivery of professionalism in the early years of Glasgow’s learner-centred, problem-based learning (PBL), integrated medical curriculum. | A qualitative approach was adopted involving semistructured interviews, on a 1 in 6 sample of tutors involved in teaching in the early curricular years, and 3 student focus groups. The findings were subjected to between-method triangulation. | Involvement in teaching raised students’ and tutors’ awareness of their professionalism. Learning activities promoting critical reflection were most effective. The integration of professionalism across the domains of Vocational Studies (VS) was important for learning; however, it was not well integrated with the PBL core. Integration was promoted by having the same tutor present throughout all VS sessions. Early patient contact experiences were found to be particularly important. The hidden curriculum provided both opportunities for, and threats to, learning. The small-group format provided a suitable environment for the examination of  pre-existing perspectives. The portfolio was an effective learning tool, although its assessment should be formalised. | Reflection is integral to professional development. Early clinical contact is an important part of the process of socialisation, as it allows students to enter the community of practice that is the medical profession. Role models can contribute powerfully to students’ learning and identity formation. As students move towards fuller participation, the clinical milieu should be controlled to maximise the influence of role models, and opportunities for guided reflection should be sustained. | NA | 23 |
| Gordon, J. 2003 | Assessing students’ personal and professional development using portfolios and interviews | Quantitative | Medical schools are placing more emphasis on students’ personal and professional development (PPD) and are seeking ways of assessing student progress towards meeting outcome goals in relation to professionalism. The Faculty of Medicine at the University of Sydney sought an assessment method that would demonstrate the value of reflection in attaining PPD, provide feedback and encourage students to take responsibility for setting and achieving high standards of performance. | The instruments used to assess Year 1 students in PPD are a portfolio and interview. This assessment format encourages students to explore ideas and values that are important to them and relevant to the PPD theme. A confidential interview, based on the PPD goals, is held with a faculty member who has read the student’s portfolio. | In 1997 ⁄98, 96% of students agreed that they had engaged in useful reflection on their approach to the course and 91% agreed that the experience was worthwhile. A further 76% of students agreed that they could see opportunities to modify their approach in some ways as result of this exercise. | Sustained PPD is essential in equipping doctors for the varied stresses of careers in medicine. Despite, or perhaps because of, the latitude in the Year 1 assessment, both students and faculty members found the process of value. This form of assessment acknowledges that the most valid assessment formats cannot always be made reliable and that in some parts of the curriculum it is more important to demonstrate trust in students’ own motivation to become competent and mindful practitioners. The fact that the portfolio and interview are the only summative assessments in the first year emphasises the importance that the Faculty places on PPD. | 8.5 | NA |
| Michael J Green  2015 | Comics and Medicine: Peering Into the Process of Professional Identity Formation | Qualitative | Medical students experience transformative personal and professional changes during medical school. The medical education community has much to learn about how students perceive these changes, which can be dramatic and profound. | Over the past six years (2009–2014), the author has taught a course on medical graphic narratives (or comics) to fourth-year medical students. Comics synergistically combine words and images to tell stories and provide an effective vehicle for helping students reflect on and give voice to varied experiences. In this course, students critically read and discuss medically themed comics and create their own original comic depicting a formative experience from medical school. | To date, 58 students have taken the course, and each has produced an original comic. The author conducted a thematic analysis of their comics and identified the following themes: (1) how I found my niche, (2) the medical student as patient, (3) reflections on a transformative experience, (4) connecting with a patient, and (5) the triumphs and challenges of becoming a doctor. Pre/post course assessments indicate that students believe creating a comic can significantly improve a variety of doctoring skills and attitudes, including empathy, communication, clinical reasoning, writing, attention to nonverbal cues, and awareness of physician bias. Students’ comics reveal the impact of formative events on their professional identity formation. | Medical educators should explore additional ways to effectively integrate comics into medical school curricula and develop robust tools for evaluating their short- and long-term impact. | NA | 19 |
| Haidet Et Al. 2008 | The role of relationships in the professional formation of physicians: Case report and illustration of an elicitation technique | Qualitative | Studies of physicians’ professional development highlight the important effect that the learning environment has in shaping student attitudes, behaviors, and values. The objective of this study was to better understand the interplay among relationships and experiences in mediating the effects of the learning environment. | We randomly recruited 2nd- and 4th-year students from among volunteers at each of five medical schools. One interviewer at each school conducted a face-to-face, open-ended, semi-structured interview with each student. The interviewers used a method called ‘life-circle diagramming’ to direct the student to draw a picture of all of the relationships in his/her life that had an influence on the sort of doctor that each student saw him/herself becoming. Interviews lasted between 60 and 120 min. Using a narrative framework that focuses on elements of students’ stories (e.g., setting, characters, plot), we analyzed transcripts through an iterative process of individual reading and group discussion to derive themes and relationships among themes. | Twenty students completed interviews. These students are embedded in complex webs of relationships with colleagues, friends, family, role models, patients, and others. Most students entered medical school with formed notions of what they wanted to ‘be like’ as physicians. While students generally gravitated toward relationships with like-minded people, their experiences varied, and some students could sense themselves changing as they moved through school. Such changes were often related to important events or issues. The relationships that students found themselves in during the context of these events had an important effect on students’ beliefs about what kinds of behaviors and attitudes were possible and desirable in their future practice. | Students proceed through medical school embedded in complex webs of relationships that exert a powerful influence (both positive and negative) on their formation as physicians. Practice Implications: Educational interventions that foster adoption of professional values need to acknowledge the influence of relationships, and assist students to harness and shape relational effects on their growth and development. The life-circle diagramming activity holds potential to promote reflection and self-knowledge, and to provide a foundation for professional growth. | NA | 22 |
| Hall Et Al. 2012 | Developing a Sustainable Electronic Portfolio (ePortfolio) Program That Fosters Reflective Practice and Incorporates CanMEDS Competencies Into the Undergraduate Medical Curriculum | Commentary | The University of Ottawa (uOttawa) Faculty of Medicine in 2008 launched a revised undergraduate medical education (UGME) curriculum that was based on the seven CanMEDS roles (medical expert, communicator, collaborator, health advocate, manager, scholar, and professional) and added an eighth role of person to incorporate the dimension of mindfulness and personal well-being. | In this article, the authors describe the development of an electronic Portfolio (ePortfolio) program that enables uOttawa medical students to document their activities and to demonstrate their development of competence in each of the eight roles. The ePortfolio program supports reflective practice, an important component of professional competence, and provides a means for addressing the “hidden curriculum.” It is bilingual, mandatory, and spans the four years of UGME. It includes both an online component for students to document their personal development and for student–coach dialogue, as well as twice-yearly, small-group meetings in which students engage in reflective discussions and learn to give and receive feedback. | The authors reflect on the challenges they faced in the development and implementation of the ePortfolio program and share the lessons they have learned along the way to a successful and sustainable program. | These lessons include switching from a complex information technology system to a user-friendly, Webbased blog platform; rethinking orientation sessions to ensure that faculty and students understand the value of the ePortfolio program; soliciting student input to improve the program and increase student buy-in; and providing faculty development opportunities and recognition. | NA | NA |
| Lack Et Al. 2019 | Evaluation of a compulsory reflective group for medical students | Mixed Methods | The ability to reflect – reflection – taking time to stop, think and evaluate is an important professional skill to develop.  To evaluate a compulsory reflective group activity to determine whether compulsory participation enabled students to constructively share emotional clinical experiences and develop ethical and professional behaviour. | This was a case study with mixed methodology. Participants were Years 5 and 6 medical students at the University of Auckland, New Zealand. Data collection included pre- and postreflective group questionnaires with Year 5 and 6 students, questionnaires with general practice academic facilitators, and audiotapes of the reflection group discussions. | Students shared emotional experiences that were organised into three themes: (i) witnessing unprofessional behaviour; (ii) meeting difficult clinical scenarios for the first time; and (iii) the hierarchy of medicine. They reported positive learning experiences relevant to their future practice and valued the opportunity to share their experiences safely. Facilitators thought the groups provided unique educational opportunities that students appreciated. Eighty-two percent of participants would like to repeat the activity during their medical school training. | Self-reflection is an essential condition for professionalism. Use of reflective groups can help students become ethical and professional doctors. | 11 | 22 |
| Lutz Et Al. 2017 | Enhancing medical students’ reflectivity in mentoring groups for professional development – a qualitative analysis | Qualitative | Professional competence is important in delivering high quality patient care, and it can be enhanced by reflection and reflective discourse e.g. in mentoring groups. However, students are often reluctant though to engage in this discourse. A group mentoring program involving all preclinical students as well as faculty members and co-mentoring clinical students was initiated at Witten-Herdecke University. This study explores both the attitudes of those students towards such a program and factors that might hinder or enhance how students engage in reflective discourse. | A qualitative design was applied using semi-structured focus group interviews with preclinical students and semi-structured individual interviews with mentors and co-mentors. The interview data were analyzed using thematic content analysis. | Students’ attitudes towards reflective discourse on professional challenges were diverse. Some students valued the new program and named positive outcomes regarding several features of professional development. Enriching experiences were described. Others expressed aversive attitudes. Three reasons for these were given: unclear goals and benefits, interpersonal problems within the groups hindering development and intrapersonal issues such as insecurity and traditional views of medical education. Participants mentioned several program setup factors that could enhance how students engage in such groups: explaining the program thoroughly, setting expectations and integrating the reflective discourse in a meaningful way into the curriculum, obliging participation without coercion, developing a sense of security, trust and interest in each other within the groups, randomizing group composition and facilitating group moderators as positive peer and faculty role models and as learning group members. | A well-designed and empathetic setup of group mentoring programs can help raise openness towards engaging in meaningful reflective discourse. Reflection on and communication of professional challenges can, in turn, improve professional development, which is essential for high quality patient care. | NA | 28 |
| Lutz Et Al. 2013 | A reflective practice intervention for professional development, reduced stress and improved patient care—A qualitative developmental evaluation | Qualitative | Professional capabilities, such as empathy and patient-centeredness, decline during medical education. Reflective practice is advocated for teaching these capabilities. The Clinical Reflection Training (CRT) is a reflective practice intervention using the professional dilemmas faced by medical students during clinical practice. The aim of this study was to evaluate students’ perceptions of the helpfulness of the CRT and its effects on their medical education. | Eighteen semi-structured interviews were conducted with medical students who had participated in the CRT. Content analysis was used to analyze the interview data. | Medical students did not feel adequately prepared to manage the difficult personal and interpersonal problems frequently encountered in clinical practice. They reported that the CRT reduces stress, improves patient care and serves as a tool for professional development. Conclusion: The CRT may be a useful tool for developing professionalism during medical education, reducing stress and enhancing the quality of patient care. | Providing students with reflective practice training that draws on their current personal clinical problems in order to improve their clinical work may be a productive investment in personal professional development, physician health, and quality improvement. | NA | 25 |
| Maitra Et Al. 2021 | Balancing Forces: Medical Students’ Reflections on Professionalism Challenges and  Professional Identity Formation | Qualitative | Professionalism is essential in medical education, yet how it is embodied through medical students’ lived experiences remains elusive. Little research exists on how students perceive professionalism and the barriers they encounter. This study examines attitudes toward professionalism through students’ written reflections. | Family medicine clerkship students at Stanford University School of Medicine answered the following prompt: “Log a patient encounter in which you experienced a professionalism challenge or improvement opportunity.” We collected and analyzed free-text responses for content and themes using a grounded theory approach. | One hundred responses from 106 students generated a total of 168 codes; 13 themes emerged across four domains: challenging patients, interpersonal interactions, self-awareness, and health care team dynamics. The three most frequently occurring themes were interacting with emotional patients, managing expectations in the encounter, and navigating the trainee role. | Medical students view professionalism as a balance of forces. While many students conceived of professionalism in relation to patient encounters, they also described how professionalism manifests in inner qualities as well as in health systems. Interpersonal challenges related to communication and agenda-setting are predominant. Systems challenges include not being seen as the “real doctor” and being shaped by team behaviors through the hidden curriculum. Our findings highlight salient professionalism challenges and identity conflicts for medical students and suggest potential educational strategies such as intentional coaching and role-modeling by faculty. Overall, students’ reflections broaden our understanding of professional identity formation in medical training. | NA | 15 |
| Markham Jr Et Al. 2021 | The Effect of Reflection Rounds on Medical Students’ Empathy | Quantitative | Students’ decline in empathy during medical school raises concerns. Empathic physician-patient interactions positively impact health outcomes and patient satisfaction, while improving job satisfaction and reducing physician burnout. Reflection Rounds may be suitable for maintaining student empathy during training. Our hypothesis was Reflection Rounds would increase empathy scores of MS3s and results would vary by gender. | MS3s completed the Jefferson Scale of Empathy upon starting and finishing their clerkship. Students in the experimental group attended four 1-hour sessions, led by a clinician and pastoral team member. The control group did not attend sessions. | No significant difference in baseline empathy scores was found between the two groups. There was a significant increase in student empathy scores among the experimental group, improving mean score from 114.1 to 116.5, p-value of 0.04. There were no significant changes among the students who did not participate in Reflection Rounds. Differences were found according to gender, with women scoring higher overall than men at baseline and increasing after sessions. | These results indicate that Reflection Rounds can improve the empathy of medical students, and warrant further investigation into their effects and utility within medical education. | 10 | NA |
| Matsuyama Et Al. 2021 | Limited effects from professional identity formation-oriented intervention on selfregulated learning in a preclinical setting: a randomized-controlled study in Japan | Quantitative | Developing self-regulated learning in preclinical settings is important for future lifelong learning. Previous studies indicate professional identity formation, i.e., formation of self-identity with internalized values and norms of professionalism, might promote self-regulated learning. We designed a professional identity formation oriented reflection and learning plan format, then tested effectiveness on raising self-regulated learning in a preclinical year curriculum. | A randomized controlled crossover trial was conducted using 112 students at Jichi Medical University. In six one-day problem-based learning sessions in a 7-month pre-clinical year curriculum, Groups A (n = 56, female 18, mean age 21.5y ± 0.7) and B (n = 56, female 11, mean age 21.7y ± 1.0) experienced professional identity formation oriented format: Group A had three sessions with the intervention format in the first half, B in the second half. Between-group identity stages and self-regulated learning levels were compared using professional identity essays and the Motivated Strategies for Learning Questionnaire | Two-level regression analyses showed no improvement in questionnaire categories but moderate improvement of professional identity stages over time (R2 = 0.069), regardless of timing of intervention. | Professional identity moderately forms during the pre-clinical year curriculum. However, neither identity nor self-regulated learning is raised significantly by limited intervention. | 12 | NA |
| Maxwell Et Al. 2002 | Experience with Hospice: Reflections from Third-Year Medical Students | Qualitative | Nationally, there is a growing emphasis on experiential education and an interest in palliative care for health professionals. | Hospice visits were added to the family medicine community rotation for third-year medical students to provide them with first-hand exposure to the hospice experience. | Seven significant themes emerged from the students’ reflection papers: (1) the value of hospice and the supportive role of the hospice team, (2) the value of empathy, (3) the distinction between acceptance and resignation, (4) the changing face of hope, (5) an understanding of death as a natural event, (6) the quality of family caregiving, and (7) the role of the physician in caring for the dying. | The implications for medical education discussed include the value of home visits as a setting for experiences in palliative care and the effectiveness of narrative assignments for encouraging student reflection. | NA | 14 |
| McNeill Et Al. 2010 | First year specialist trainees’ engagement with reflective practice in the e-portfolio | Mixed Methods | Doctors in specialist training posts in the Mersey Deanery are expected to reflect on their clinical practice and to document their learning experiences in an e-portfolio. This study aims specifically to explore how they have engaged in reflection on their practice and how they utilise their learning portfolio to document evidence of this | A modified Delphi technique was used to develop a grading system to identify the level of reflection recorded by participants in the e-portfolio. Transcripts of the reflective accounts were then analysed using a qualitative approach which involved coding and categorising the data. | This study demonstrated a wide variation in both the quantity and quality of reflection. Of particular note in the qualitative data analysis were themes relating to clinical knowledge and skills, learning in practice, communication, feelings, types of learning experience reflected on and wider aspects of medical practice. Findings indicate there is variation is the extent to which doctors both engage in and document evidence of reflection. | Further research is needed to explore factors that enable or inhibit the use of the e-portfolio for reflection and whether recorded reflection is a true picture of the cognitive process involved. | 11 | 14 |

| **Article Title** | **Authors/ Year** | **Type of Study** | **Study Aim** | **Methodology** | **Key Findings** | **Conclusions** | **MERSQI** | **COREQ** |
| --- | --- | --- | --- | --- | --- | --- | --- | --- |
| Becoming a practitioner: workplace learning during the junior doctor's first year | D. Sheehan, T. J. Wilkinson and E. Bowie | Qualitative | To document the reflections and perceptions of first year junior doctors in order to reveal and chronicle their informal and often tacit learning in the workplace within a practice methodology framework. | New Zealand interns, from three sites, participated in group interviews modelled on a conversation and joint enquiry style. | We found that learning in the first year after graduation falls into three broad themes: (1) concrete tasks, (2) project management and (3) identity formation. Identity formation appeared the most challenging and included getting used to being seen by others as a doctor. | All themes have implications for curriculum development and clinical supervision in both undergraduate programmes and during internship. The third theme (identify formation) is the most complex. We draw on a model from management literature, to describe intern education as a process of becoming: as an unfolding and as a transformation of the self over time. We argue that reconfiguring internship as a period of identity formation, and as a self-determined, active process of ‘becoming a doctor’ provides a wider perspective than enculturation or socialisation theories to understand this significant transition. | NA | 17 |
| Competency-based curriculum for family medicine | D. Saucier, E. Shaw, J. Kerr, J. Konkin, I. Oandasan, A. J. Organek, et al. | Review article | This article is one in a series explaining the Triple C initiative. It discusses how competency-based education is at the very heart of this endeavour and provides a solid educational rationale, an organized approach, and a series of practical strategies to better reach the very goal of residency training: “to develop professional competence to the level of a physician ready to begin practice in the specialty of family medicine.” | NA | NA | Triple C describes the unique features of a competency-based residency program as they  apply specifically to a family medicine residency context. These features represent the strategies best suited to efficiently train competent future family physicians. Moving to Triple C seems to be the most fitting way to prepare future family physicians in accordance with international educational trends and societal expectations. | NA | NA |
| Developing critical reflection within an interprofessional learning programme | S. Smith and K. Karban | Descriptive study | This paper will challenge traditional uniprofessional models of reflection through the development of an approach to interprofessional learning informed by models of critical practice that seek to critique and transcend traditional professional boundaries. Attention will also be paid to the central importance of service users as the essential focus of collaborative team working. | NA | The implications of embedding critical reflection as a key component of an integrated strategy for interprofessional learning will be discussed with reference to the development of a new interprofessional learning strategy for pre registration students within which reflective practice is introduced as both a discrete unit and a continuing theme throughout the curriculum. | The paper will conclude that a model of critical and reflective practice will enable future practitioners to respond to the transformation of previously compartmentalised ways of thinking and working and the challenge of new ways of working. | NA | NA |
| Digital storytelling for reflection in undergraduate medical education: a pilot study | J. Sandars and C. Murray | Qualitative | Our hypothesis was that digital storytelling had the potential to offer an innovative approach to engage first-year undergraduate medical students in reflection. The aim of this pilot study was to test our hypothesis. | Twelve first-year undergraduate medical students volunteered to use digital storytelling for a reflective learning exercise on a personal and professional development module in October 2007. The aim of this module was to encourage students to reflect on their experience of first meeting a patient | Digital storytelling can effectively engage undergraduate medical students in reflection. The process of creating a digital story can stimulate students to carefully consider and reflect upon why  they collect, select and present the various photographs. | Digital storytelling appeared to effectively engage first-year undergraduate medical students in reflection. All stages of the digital storytelling process, from initial collection and selection of the photographs to the final presentation of the story, appeared to stimulate deeper, and more meaningful, reflection. The authors propose that digital storytelling provides an innovative and useful approach for the development of reflection and reflective learning in medical education. Further research is recommended to inform future teaching and learning practice. | NA | 13 |
| Does Mindfulness Training Enhance the Professional Development of Residents? A Qualitative Study | H. Verweij, H. van Ravesteijn, M. L. M. van Hooff, A. L. M. Lagro-Janssen and A. E. M. Speckens | Qualitative | The aim of our qualitative study was to explore the influence of MBSR on residents’ professional lives—how they work and develop as a physician, how they manage stress at work, and in the balance of their home and work responsibilities. | Between 2014 and 2016, the authors conducted 19 in-depth, face-to-face interviews with residents who had participated in an MBSR course at Radboud university medical center, the Netherlands. Medical and surgical residents, across a range of disciplines, participated. The authors used the constant comparison method to analyze the data. | The analysis of the data resulted in five themes: awareness of thoughts, emotions, bodily sensations, and behavior; increased self-reflection; acceptance and nonjudgment; increased resilience; and relating to others. Residents indicated that the MBSR training increased their awareness and self-reflection at work, and they were more accepting toward themselves and toward their limitations. Furthermore, they mentioned being more resilient and better at setting priorities and limits. They improved their self-care and work–life balance. In addition, residents indicated that the training made them more aware of how they communicated. They asked for help more often and seemed to be more open toward feedback. Lastly, they indicated an increased sense of compassion for others. | This study indicated that mindfulness training can serve as a tool to cultivate important professional competencies for residents. | NA | 27 |
| Harnessing the hidden curriculum: a four-step approach to developing and reinforcing reflective competencies in medical clinical clerkship | C. L. Holmes, I. B. Harris, A. J. Schwartz and G. Regehr | Review article | There is a powerful hidden curriculum that perpetuates not only desired attitudes and behaviors but also those that are less than desirable. So, how do we educate medical students to resist adopting unprofessional practices they see modeled by supervisors and mentors in the clinical environment? This paper explores these issues and, informed by the literature, we propose a specific set of reflective competencies for medical students as they transition from classroom curricula to clinical practice in a four-step approach | NA | (1) Priming—stu- dents about hidden curriculum in their clinical environment and their motivations to conform or comply with external pressures; (2) Noticing—educating students to be aware of their motivations and actions in situations where they experience pressures to conform to practices that they may view as unprofessional; (3) Processing—guiding students to analyze their experiences in collaborative reflective exercises and finally; (4) Choosing— supporting students in selecting behaviors that validate and reinforce their aspirations to develop their best professional identity. | By exploring these dis- courses, we have evolved a proposal for a four-step approach to development and reinforcement of reflective competencies in medical students starting prior to and continuing through their apprenticeship in clinical medicine. In this way, our students can contribute to positive change in our profession characterized by a culture of respect. Informed by the literature we have proposed a model for designing, implementing and evaluating a reflective competency curriculum that can be applied to professionalism content in medical training with possible implications for other health professional education. | NA | NA |
| Implementation of online peer feedback for student self-reflection–first steps on the development of a feedback culture at a medical faculty | B. Raski, A. Eissner, E. Gummersbach, S. Wilm, L. Hempel, M. Dederichs, et al. | Mixed study | The aim was therefore to structurally integrate feedback into the curriculum of a model study course in order to develop a feedback culture in which students can develop personally and professionally with the help of regular and constructive feedback. | Following an initial pilot phase in 2009, (peer) feedback was gradually integrated into the curriculum at the medical faculty, in the first instance through checklists and subsequently through an online questionnaire and direct interviews. The activities were regularly analyzed on the basis of student evaluations using the EvaSys evaluation software and semi-standardized questionnaire-based interviews with six students in 2009 and 13 students each in 2012 and 2013. | Qualitative data showed that students had a more positive perception or assessment of the location’s feedback concept as well as indications of improvements in the culture of trust at the location. The proportion of constructive free-text comments increased significantly by 11% to 99.4% com- pared to the previous year (t(3)=-3.79, p=0.04). Thus, in terms of the objective, an increase in feedback activities and their quality at the faculty was achieved. | Feedback, its acceptance as well as the quality, can be positively influenced at a faculty. Change measures should be tested repeatedly in discussion with users regarding practicability in order to directly pick up implementation issues and obstacles so they can be remedied in the interests of the users. This can influence the development of a culture of trust and feedback and should promote the personal and professional development of students in the long term. | 8.5 | 13 |
| A participant perspective on collaborative reflection: video-stimulated interviews show what residents value and why | M. van Braak, E. Giroldi, M. Huiskes, A. D. Diemers, M. Veen and P. van den Berg | Qualitative | In our study, therefore, we explore participants’ views on the value of an educational activity of which the aim is to collaboratively reflect on professional practice (van Braak et al. submitted). We focus particularly on their views about the mechanisms that explain why certain aspects of the activity do or do not create educational value. | To identify valuable and less valuable features of collaborative reflection, we conducted one-on-one video-stimulated interviews with Dutch general practice residents about collaborative reflection sessions in their training program. Residents were invited to comment on any aspect of the session that they did or did not value. | We synthesized all positively and negatively valued features and associated explanations put forward in residents’ narratives into shared normative orientations about collaborative reflection: what are the shared norms that residents display in telling about positive and negative experiences with collaborative reflection? These normative orientations display residents’ views on the aim of collaborative reflection (educational value for all) and the norms that allegedly contribute to realizing this aim (inclusivity and diversity, safety, and efficiency). These norms are also reflected in specific educational activities that ostensibly contribute to educational value. | The current synthesis of normative orientations displayed in residents’ narratives about valuable and less valuable elements of collaborative reflection deepen our understanding of reflection and its supposed connection with educational outcomes. Moreover, the current empirical endeavor illustrates the value of video-stimulated interviews as a tool to value features of educational processes for future educational enhancements. | NA | 20 |
| Professional formation: extending medicine's lineage of service into the next century | M. W. Rabow, R. N. Remen, D. X. Parmelee and T. S. Inui | Commentary | NA | NA | Key elements include experiential and reflective processes, use of personal narratives, integration of self and expertise, and candid discussion within a safe community of learners. Committing to professional formation within medical education will require transformation of formal and informal curricula and will necessitate a rebalancing of attention and financial support within schools of medicine. | Professional formation education offers both students and faculty the support to make their foundational values the principles of action in daily life and to bring their whole selves to work. The personal rewards of healing the divided life are integrity, self-respect, and connection. By weaving all of Flexner’s remarkable vision into medical education, we will reclaim in the present the values that have distinguished the lineage of medicine over generations: compassion, healing, and service. | NA | NA |
| Professional identity formation in medical education for humanistic, resilient physicians: pedagogic strategies for bridging theory to practice | H. S. Wald, D. Anthony, T. A. Hutchinson, S. Liben, M. Smilovitch and A. A. Donato | Review article | NA | NA | The authors present three pedagogic innovations contributing to the PIF  process within undergraduate and graduate medical education (GME) at  their institutions. These are (1) interactive reflective writing fostering reflective capacity, emotional awareness, and resiliency (as complexities within physician– patient interactions are explored) for personal and professional development; (2) synergistic teaching modules about mindful clinical practice and resilient responses to difficult interactions, to  foster clinician resilience and enhanced well-being for effective professional functioning; and (3) strategies for effective use of a professional development e-portfolio and faculty development of reflective coaching skills in GME. | These strategies as “bridges from theory to practice” embody and integrate key elements of promoting and enriching PIF, including guided reflection, the significant role of relationships (faculty and peers), mindfulness, adequate feedback, and creating collaborative learning environments. Ideally, such pedagogic innovations can make  a significant contribution toward enhancing quality of care and caring with resilience for the being, relating, and doing of a humanistic health care professional. | NA | NA |
| Reflection as a learning tool in graduate medical education: a systematic review | A. F. Winkel, S. Yingling, A.-A. Jones and J. Nicholson | Systemic review | A systematic review of the literature examined interventions reporting the use of reflection in graduate medical education. | The authors searched Medline/PubMed, Embase, Cochrane CENTRAL, and ERIC for studies of reflection as a teaching tool to develop medical trainees’ capacities. Key words and subject headings included reflection, narrative, residents/GME, and education/teaching/learning. No language or date limits were applied. The search yielded 1308 citations between inception for each database and June 15, 2015. A total of 16 studies, encompassing 477 residents and fellows, met eligibility criteria. Study quality was assessed using the Critical Appraisal Skills Programme Qualitative Checklist. The authors conducted a thematic analysis of the 16 articles. | Outcomes studied encompassed the impact of reflection on empathy, comfort with learning in complex situations, and engagement in the learning process. Reflection increased learning of complex subjects and deepened professional values. It appears to be an effective tool for improving attitudes and comfort when exploring difficult material. | Critical reflection is a tool that can amplify learning in residents and fellows. Added research is needed to understand how reflection can influence growth in professional capacities and patient-level outcomes in ways that can be measured. | NA | NA |
| Reflection in Medical Education | K. Hargreaves | Perspective paper | This paper offers a medical-education perspective that I will hope complement other disciplinary perspectives in examining the value of reflection for learning in tertiary education. | NA | NA | The paper concludes with the proposition that collaborative forms of reflection need to include more involvement of patients, interprofessional communities of practice and the use of learning from other disciplines; all of these require different levels of thinking and different ways of working. | NA | NA |
| Reflection in Rural Family Medicine Education | R. Ohta and C. Sano | Qualitative | This study aimed to investigate the framework of effective reflection in rural family medicine education. | This qualitative research was conducted to clarify if reflection in rural family medicine education improves residents’ development. Ethnography and interviews were conducted from 1 April 2020 to 31 December 2021. | The contents of reflection include clinical issues regarding knowledge and skills, professionalism in clinical decisions, and work-life balance. The settings of reflection include conference rooms, clinical wards, residents’ desks, and hospital hallways. The timing of educational reflection includes during and after patient examination and discussion with various professionals, before finishing work, and during “doorknob” times (right before going back home) | Rural medical teachers need competence as clinicians and medical educators to promote learning in medical residents and sustain rural medical care. Furthermore, medical teachers must communicate and collaborate with medical residents and nurses for educational reflection to take place in rural family medicine education, especially regarding professionalism. In rural family medicine education, reflection can be performed in various clinical situations through collaboration with learners and various medical professionals, aiding the enrichment of residents’ learning and sustainability of rural medical care. | NA | 17 |
| Reflective practice as a tool to teach digital professionalism | J. W. Kung, R. L. Eisenberg and P. J. Slanetz | Quantitative | Digital professionalism is increasingly being integrated into postgraduate medical education. We developed a small-group, reflective practice–based session incorporating radiology-specific cases to heighten residents’ awareness about digital professionalism. | Case-based, radiology-specific scenarios were created for a small-group, reflective practice–based session on digital professionalism. Anonymous pre- and postsession surveys evaluating residents’ use of social media and their thoughts about the session were administered to the radiology residents. | Twenty-five of 38 (66%) residents responded to the presession survey with 40% (10/25) reporting daily social media use; 50% (12/24) witnessing an unprofessional posting on Facebook; and 8% (2/25) posting something unprofessional themselves. Of the 21 residents who attended the session, 13 (62%) responded to the postsession survey. Residents reported that the session added to their under- standing of professionalism 3.92, 95% CI (3.57–4.27). As a result of the session, residents stated that they were more aware of protecting patient privacy and confidentiality on social media sites 3.92, 95% CI (3.47–4.37), and would take a more active role in ensuring professional use of social media as it relates to patient care 4.00, 95% CI (3.66–4.34). | Residents favorably viewed the reflective case–based session on digital professionalism as a means to be more aware of ways to avoid unprofessional interactions on the internet. Our results suggest that such reflective sessions are an effective method to educate residents on key concepts regarding digital professionalism. | 11 | NA |
| The reflective practitioner : how professionals think in action | D. A. Schön | Book review | NA | NA | NA | Nonetheless, despite the criticism, I think this is an extremely valuable book that anyone interested in the education of managers would do well to consider. Moreover, for those with little background in contemporary philosophy of science, this book might serve as the beginning of a useful corrective for what has become, in their own role as research professionals, embarrassingly an achronistic methodology. | NA | NA |
| "Safe Harbor": Evaluation of a Professionalism Case Discussion Intervention for the Gross Anatomy Course | C. M. Spampinato, C. M. Wittich, T. J. Beckman, S. S. Cha and W. Pawlina | Quantitative | The aim of this study was to determine if professionalism case discussions during a Gross Anatomy course improve students’ reflections using a validated reflection instrument (12 items; five-point Likert scale where 1 5 Disagree, 2 5 Disagree with reservation, 3 5 Neutral, 4 5 Agree with reservation, 5 5 Agree). | Reflection on professionalism scores were determined using a validated reflection instrument before and after the curriculum intervention. The previously reported instrument included 12 items based on a five-point scale that stratified  reflection into low, moderate, and high levels (Wittich et al., 2013). Additionally, post-intervention reflection scores were compared with a historical control of first-year gross anatomy students (N 5 95) at the Mayo Medical School from 2009 and 2010, to whom small group discussion sessions were not offered. | Results did not show a significant change between pre-and postintervention reflection scores (3.45 6 0.61 vs. 3.48 6 0.51; P 5 0.82). Historical control students were found to have significantly higher reflection scores when compared with postintervention students (3.91 6 0.53 vs. 3.48 6 0.51; P < 0.001). However, the historical control students were found to have significantly higher professionalism scores (P 5 0.001) as com- pared with the intervention students. Student satisfaction was high, with 25 of 28 (89.2%) students reporting that the sessions should be included as a component of future anatomy courses. | While reflection scores were not significantly increased as a result of the intervention, students expressed appreciation for the opportunity to discuss professionalism issues related to the dissection of cadavers. Additionally, the intervention students had both lower professionalism scores and lower reflection scores, which supports the idea that highly professional students are more capable of reflecting on professional- ism. Future studies should determine whether this case discussion intervention improves objective measures of professionalism. | 11 | NA |
| (Self-)Reflexion and training of professional skills in the context of "being a doctor" in the future - a qualitative analysis of medical students' experience in LET ME ... keep you real! | L. Scheide, D. Teufel, M. Wijnen-Meijer and P. O. Berberat | Qualitative | This paper seeks to assess how medical students can be trained in medical studies seminars to examine their own professional role as doctors. | The source data is statements made by medical students who took part in the LET ME ... keep your real! seminar from 2016- 2018. Student perspectives were analyzed after five focus group discussions with a total of 26 medical student participants and two individual interviews. Based on the interpretative paradigm and following the credo of a methodological exploration of medical students’ lifeworld, their specific learning experience as well as their ability for (self-) reflection were mapped out. | From the students’ standpoint, five skills can be identified that make this behavior possible:  1. questioning and doubting,  2. recognizing relevant perspectives,  3. classifying viewpoints,  4. maintaining communal exchanges and  5. deciding on a (different) position.  Situatively, these skills are often used in combination and challenge students on an intellectual, communicative and emotional level. | The ability to (self-)reflect should be more strongly integrated in university medical training by providing appropriate support, especially since it presents students with specific challenges to (self-)reflexively approach their own future as doctors. The skills mapped out here can be used as orientation to develop seminars on professional (self-)reflexive identity development for medical students. | NA | 18 |
| A systematic scoping review of reflective writing in medical education | J. Y. Lim, S. Y. K. Ong, C. Y. H. Ng, K. L. E. Chan, S. Y. E. A. Wu, W. Z. So, et al. | Systematic scoping review | A Systematic Scoping Review is proposed to map current understanding of RW programs. It is hoped that this SSR will also identify gaps in knowledge and regnant practices, pro- grams and assessment methods to guide the design of RW programs. | A Systematic Evidence-Based Approach guided Systematic Scoping Review (SSR in SEBA) was adopted to guide and structure the two concurrent reviews. Independent searches were carried out on publications featured between 1st January 2000 and 30th June 2022 in PubMed, Embase, PsychINFO, CINAHL, ERIC, ASSIA, Scopus, Google Scholar, OpenGrey, GreyLit and ProQuest. The Split Approach saw the included articles analysed separately using thematic and content analysis. Like pieces of a jigsaw puzzle, the Jigsaw Perspective combined the themes and categories identified from both reviews. The Funnelling Process saw the themes/categories created compared with the tabulated summaries. The final domains which emerged structured the discussion that followed. | A total of 33,076 abstracts were reviewed, 1826 full-text articles were appraised and 199 articles were included and analysed. The domains identified were theories and models, current methods, benefits and shortcomings, and recommendations. | This SSR in SEBA suggests that a structured approach to RW shapes the physician’s belief system, guides their practice and nurtures their professional identity formation. In advancing a theoretical concept of RW, this SSR in SEBA proffers new insight into the process of RW, and the need for longitudinal, personalised feedback and support. | NA | NA |
| Teaching humanism | D. T. Stern, J. J. Cohen, A. Bruder, B. Packer and A. Sole | Descriptive paper | By describing educational experiences that both promote and sustain humanism in doctors, we hope to stimulate the thinking of other medical educators and to disseminate the impact of these innovative educational programs to help the profession meet its obligation to provide the public with humanistic physicians. | NA | NA | Common elements of programs that effectively teach humanism include: (1) opportunities for students to gain perspective in the lives of patients; (2) structured time for reflection on those experiences; and (3) focused mentoring to ensure that these events convert to positive, formative learning experiences. | NA | NA |
| Twelve tips for teaching reflection at all levels of medical education | L. Aronson | Literature review | The following tips outline an approach to the design, implementation, and evaluation of reflection in medical education. | The method is based on the available literature and the author’s experience. They are organized in the sequence that an educator might use in developing a reflective activity. | The 12 tips provide guidance from conceptualization and structure of the reflective exercise to implementation and feedback and assessment. The final tip relates to the development of the faculty member’s own reflective ability. | With a better understanding of the conceptual frameworks underlying critical reflection and greater advance planning, medical educators will be able to create exercises and longitudinal curricula that not only enable greater learning from the experience being reflected upon but also develop reflective skills for life-long learning. | NA | NA |

| **Authors/Year** | **Article Title** | **Type of Study** | **Study Aim** | **Methodology** | **Key Findings** | **Conclusions** | **MERSQI**  **(quantitative)** | **COREQ**  **(qualitative)** |
| --- | --- | --- | --- | --- | --- | --- | --- | --- |
| N. Lumlertgul, N. Kijpaisalratana, N. Pityaratstian and D. Wangsaturaka | Cinemeducation: A pilot student project using movies to help students learn medical professionalism | Qualitative | This article reports a student project, ‘‘Cinemeducation’’, which aimed to utilize feature films as a tool to help promote the students’ professionalism. | Five movies with professionalism issues were screened with 20–30 students attending each session. After the show, participants then were asked to reflect on what they had learned in terms of professionalism. Two students led group discussion emphasizing questioning and argumentation for 60 min. Additional learning issues emerging from each session were also explored in more depth and arranged into a report. | In the Cinemeducation Project, medical students have learned five main ethical issues in each film, which were the doctor–patient relationship, informed consent and clinical trials in patients, management of genetic disorders, patient management, and brain death and organ transplantation. In addition to issues of professionalism, they also developed critical thinking and moral reasoning skills. | Using a case-based scenario in movies has proven to be an effective and entertaining method of facilitating students with learning on professionalism. | NA | 11 |
| S. Schrempf, L. Herrigel, J. Pohlmann, J. Griewatz and M. Lammerding-Köppel | Everybody is able to reflect, or aren't they? Evaluating the development of medical professionalism via a longitudinal portfolio mentoring program from a student perspective | Qualitative | This study examines medical students' attitudes toward professional reflection and toward the program in general to draw conclusions about conditions as well as the needs-based design of the program. | In winter semester 2017/18, a retrospective questionnaire survey with free text fields was conducted (total sample: N=1.405; students S 1-9; response 37%; S 1-4 “Pre-clinic”: n=231; S 5-9 “Clinic”: n=241). Opinion trends of semester groups were identified through seven semi-structured interviews with semester speaker and peer tutors. | Differences in understanding and attitudes resulted in three positions: 1=approval, 2=ambivalence, 3=rejection. All three groups included individuals from pre-clinical and clinical settings with varying levels of experience. Prior experience and hidden curriculum influenced the position. Opinion trends confirmed the feedback. | Although reflection appears in the National Competency based Learning Objectives Catalogue for Medicine (NKLM), reflective competence is not regarded as equivalent to other study content. Motivation, commitment on the part of the mentors, and a trusting mentor mentee relationship are effective. The flexibility of the portfolio in terms of content and methodology, as well as the curricular integration of the program are also beneficial. | NA | 16 |
| A. Riskin, G. Yakov and A. A. Flugelman | Group Mentoring for Junior Medical Students-the Mentor in the Reflection Cycle | Quantitative | To describe the experience and perceptions of physicians involved in group mentoring of undergraduate pre-clinical medical students | We conducted a cross-sectional questionnaire-based survey on perceptions of mentors regarding their motivation, personal development, reflective experience, and burnout. All the participants were mentors to undergraduate pre-clinical medical students in the course "Becoming a Physician." This unique course focuses on various aspects of medical professionalism and aims to increase awareness and sensitivity to patients, especially of disadvantaged populations, and to promote sensitive effective communication skills. Mentors in the course are expected to serve as role models to their students. | Of 36 mentors, 33 (91.7%) responded. The most frequent motivations to join the course were to contribute to students' personal, social, and professional development and to contribute to educating more compassionate physicians. The topics discussed most in the groups were the meaning of being a physician and ethical dilemmas. Mentors expressed that they gained professional growth and opportunities to reflect on the complexity of physicians' training and work. They perceived their highest success as being able to serve as role models for their students and provide them broad perspectives. Mentors stated that they failed in trying to facilitate content learning, and were disturbed by students' lack of punctuality. Group mentors scored relatively low on the Maslach Burnout Inventory for Physicians | This study provides insights on the experience of mentorship of medical school students, and on mentors' perceptions regarding their teaching experience and personal and professional development | 12 | NA |
| J. Park, S. I. Woodrow, R. K. Reznick, J. Beales and H. M. MacRae | Observation, reflection, and reinforcement: Surgery faculty members' and residents' perceptions of how they learned professionalism | Qualitative | To explore perceptions of how professionalism is learned in the current academic environment. Professionalism is a core competency in surgery (as in all of medical practice), and its presence or absence affects all aspects of clinical education and practice, but the ways in which professional values and attitudes are best transmitted to developing generations of surgeons have not been well defined. | The authors conducted 34 semistructured interviews of individual surgery residents and faculty members at two academic institutions from 2004 to 2006. Interviews consisted of open-ended questions on how the participants learned professionalism and what they perceived as challenges to learning professionalism. Two researchers analyzed the interview transcripts for emergent themes by using a grounded-theory approach. | Faculty members’ and residents’ perceptions of how they learned professionalism reflected four major themes: (1) personal values and upbringing, including premedical education experiences, (2) learning by example from professional role models,  (3) the structure of the surgery residency, and (4) formal instruction on professionalism. Of these, role modeling was the dominant theme: Participants identified observation, reflection, and reinforcement as playing key roles in their learning from role models and in distinguishing the sometimes blurred boundary between positive and negative role models. | The theoretical framework generated out of this study proposes a focus on specific activities to improve professional education, including an active approach to role modeling through the intentional and explicit demonstration of professional behavior during the course of everyday work; structured, reflective self-examination; and timely and meaningful evaluation and feedback for reinforcement. | NA | 21 |
| Stephanie W. Zuo*, Cody Cichowitz, Robert Shochet, MD, Arun Venkatesan, MD, PhD | Peer-Led, Postanatomy Reflection Exercise in Dissection  Teams: Curriculum and Training Materials | Qualitative | he importance of emphasizing empathy, reflection, and professionalism during anatomy dissection has been well established. This small-group curriculum was developed to fulfill a need for structured reflection at the end of anatomy. | In this 45-minute reflection session, each dissection team of first-year medical students in anatomy is led by one or two peer facilitators recruited from the second-year medical student class. The session is designed to include a time for sharing of personal reflections, a clinical observation activity about the cadaver’s cause of death, and an appreciative inquiry approach to the dissection team experience. In addition to the reflection session curriculum, materials also include a 1-hour presession training module, containing a small-group facilitator skill-building and role-play and a 30-minute postsession debrief for peer facilitators. | e found that the majority of anatomy students felt that the end-of-course reflection was a meaningful way to conclude the course and that the session had a positive impact on their relationship with their dissection team. Fifteen peer facilitators participated in focus groups, and common themes included the value of giving back, making meaning of past experiences, countering burnout by recognizing one’s own growth, and continued learning through peer teaching. | This anatomy reflection curriculum has been incorporated into our 7-week anatomy course and has been well received by both anatomy students and peer facilitators. We believe that peer-led small-group reflection sessions after intensive experiences in medical school can promote personal and professional growth among both junior and senior medical students. | NA | 14 |
| M. Siedsma and L. Emlet | Physician burnout: Can we make a difference together? | .Quantitative | . To test the hypothesis that an intervention involving a facilitated physician small-group curriculum would result in improvement in well-being | A randomized clinical trial of practicing physicians. Additional data were collected on non-trial participants responding to annual surveys timed to coincide with the trial surveys. | The intervention involved 19 biweekly facilitated physician discussion groups incorporating elements of mindfulness, reflection, shared experience, and small-group learning for 9 months. Protected time (1 hour of paid time every other week) for participants was provided by the institution.. | Meaning in work, empowerment and engagement in work, burnout, symptoms of depression, quality of life, and job satisfaction were assessed using validated metrics. | 10 | NA |
| G. R. Tait and B. D. Hodges | Residents Learning from a Narrative Experience with Dying Patients: A Qualitative Study | Qualitative | In our current study we designed an educational intervention for psychiatry and family medicine residents that aimed to address gaps in the psychological, spiritual, and existential care of dying patients. Specifically, we used the dignity interview, an empirically based psychotherapeutic intervention that was developed, based on patients’ perspectives, to address these very concerns (Chochinov et al. 2005). | Seven family medicine residents (five female and two male) and five psychiatry residents (three female and two male), all in their first year of residency, were recruited during a first year rotation in palliative care. Participants were provided with a brief learning guide that introduced the intervention, its significance, and the protocol for the study. On the day of the intervention, a brief session with the principal investigator occurred to review this material and prepare for the inter- view. While the principal investigator was trained in dignity therapy at one of the trial sites, Memorial Sloan Kettering Cancer Center, the intention was not to train the residents to be experts in the interview. | Most residents reported that their actual comfort level was greater than anticipated.  Residents expressed a sense of ownership and responsibility for editing and creating the physical document. Editing the documents often stimulated reflection on the stories and even further learning about historical events, such as the Holocaust. Words such as ‘‘honor’’, ‘‘privilege’’, and ‘‘reward’’ described residents’ experiences of being part of the intervention. | In addition to the influence of formal learning about communication, the residents in our study pointed to powerful messages embedded in the hidden curriculum. They described a lack of role modeling and feedback on difficult conversations, and a message that there isn’t time to listen to a person’s story. Further, they are learning that getting ‘emotional’ or  ‘close’ may actually be seen as unprofessional or even dangerous, especially when it puts ‘‘self preservation’’ at risk (Borgstrom et al. 2010). | NA | 16 |
| S. Parker and A. Leggett | Teaching the clinical encounter in psychiatry: a trial of Balint groups for medical students | Qualitative | Balint groups are being trialled as a method to facilitate understanding of the relational aspects of student encounters with psychiatric patients. This paper reports on the establishment, processes and trends in the student evaluations of these groups. | The groups have been introduced as part of the medical student curriculum at a tertiary referral hospital. In six of the eight weeks of the clinical rotation in psychiatry, students meet in a group led by the authors, to discuss relational aspects of their interactions with patients. Ten third-year postgraduate medical students participate in the group each rotation. The educational value of each meeting and the group overall is assessed using questionnaires. | The groups tended to be rated positively by the participants. However, students were less certain of the relevance to their clinical practice. Vignettes demonstrating aspects of group process are presented in the context of the leaders’ experiential account. | Short-term clinical reflection groups can be effectively implemented for medical students in a hospital environment. These groups have the potential to support students in the process of learning to work in doctor– patient relationships, but may encounter significant challenges necessitating adaptation of method and process to context. | NA | 13 |
| D. A. Taylor, V. Gorski and S. K. Burge | Using Reflections to Evaluate the STFM Behavioral Science/Family Systems Educator Fellowship | Qualitative | A group of family medicine educators identified a need and developed a 1-year fellowship for early career behavioral science educators. This occurred in response to a reduction in previous opportunities and resources. The program was designed to shape and men- tor new behavioral science faculty teaching in family medicine departments and programs. Quantitative data analysis from pre- and post-fellowship survey data from years 1-4 confirmed fellowship objectives were met. | The 1-year fellowship, developed by senior faculty in STFM features a blend of classroom style learning, mentored small-group interactions, reflective writing and a scholarly project requirement. As one aspect of pro- gram evaluation, reflective writings submitted by fellows and faculty were analyzed using qualitative methodology for themes related to curricular objectives. | From 2010-2013, 44 fellows completed the program. Authors analyzed reflective writings from 15 fellows and 6 small-group mentors. Four overarching themes emerged: emerging professional competence, evolving professional identity, connectedness, and generativity. An unexpected finding was that the fellowship mentors benefited in ways parallel to that of the fellows. | A qualitative analytical approach to examining the reflective writings of fellowship participants yielded confirmation that program goals were achieved. In addition, a commitment to “paying it forward” as ongoing and future leaders in family medicine education resulted for both fellows and faculty mentors. | NA | 17 |

| **Authors/Year** | **Article Title** | **Type of Study** | **Study Aim** | **Methodology** | **Key Findings** | **Conclusions** | **MERSQI** | **COREQ** |
| --- | --- | --- | --- | --- | --- | --- | --- | --- |
| Zumwalt, A. C. | Anticipatory Feelings About Dissection: An Exercise for the First Day of a Gross Anatomy Course | Qualitative | The exercise described here provides an opportunity to start a conversation about the complexity of students’ emotional reactions to the anatomy experience. The intention of this exercise is to normalize the variety of emotions that anatomy students experience, both to demonstrate to students that their emotions are normal and to encourage the empathy for others’ reactions which may differ from their own. | In the lecture hall setting before the first day of dissection, students are asked to draw how they feel about the dissection experience and are provided an opportunity to discuss their drawings with their peers. The course director then provides a slide show demonstration of drawings from previous years, and experienced anatomy faculty facilitate a large group discussion in which students react to the drawing exercise and slide show and ask questions which are addressed by the faculty. | A number of the focus group participants expressed that they found the drawing aspect of the exercise to be particularly challenging. They had difficulty identifying and dis- tilling their feelings into something they could draw, and one student felt particularly challenged by this task due to a preference for writing rather than drawing. One response to this feedback could be to modify the exercise to allow the students more modalities (e.g., writing, images, and music) to respond to the question. However, part of the value in the exercise is in the challenge of this assignment. The unusual act of drawing an emotion creates a challenge that compels full engagement with the task, which is arguably one of the reasons that projective drawing exercises are powerful (Oster and Crone, 2004). In addition, by obliging students to express themselves through a simple line drawing, the focus | In summary, the exercise described here is a useful tool for anatomy educators to acknowledge the complex emotions elicited by the cadaver dissection experience. It encourages student introspection and contemplation of their anticipatory feelings about the anatomy laboratory experience and can be useful to establish communication within the class about topics that can be otherwise difficult to discuss. It is simple to implement and scalable to many types of student populations and curriculum structures, and also has potential to be expanded in numerous ways. Exercises such as the one described here foster a culture within the anatomy course that normalizes students’ emotional reactions to the dissection experience and the practice of acknowledging those experiences. This practice in turn facilitates student empathy for others’ experiences and provides the opportunity to practice healthy mechanisms for coping with emotionally complex situations. Ultimately this exercise provides a foundation for numerous important aptitudes and skills that serve a healthcare professional. | NA | 17 |
| Rucker, L.  Shapiro, J. | Becoming a physician: Students' creative projects in a third-year IM clerkship | Qualitative | Medical educators have only limited understanding of how integrating humanities-based components into standard curricula contributes to the medical students’ professionalism. This study qualitatively analyzed how students used a creative-project assignment during their third-year internal medicine clerkships to explore various aspects of their professional development. | A total of 277 students from three consecutive classes (1999–2002) at the University of California, Irvine, College of Medicine each completed a creative project reflecting on a particularly problematic or meaningful illness-related incident. Process and content analyses of the 221 projects submitted for analysis were performed. | Students’ projects employed a wide range of formats, tones, and styles to examine the process of socialization into medicine. Within this framework, their work tended to explore issues such as the proper relationship of medical students to patients, coming to terms with death and dying, understanding the patient’s experience of illness, and coping with professional and personal stress. | A creative-projects course component can be a valuable adjunct to traditional clerkship activities in helping students to reflect on the process of becoming a physician. | NA | 18 |
| Riskin, A.  Kerem, N. C.  Van-Raalte, R.  Kaffman, M.  Yakov, G.  Aizenbud, D.  Ben-Barak, A.  Shachor-Meyouhas, Y.  Edery, R.  Shabtai Musih, Y.  Sagi, S.  Eidelman, O.  Rotschild, A. | 'Becoming a Physician'-medical students get acquainted with disadvantaged populations, and practise sensitive and effective communication | Qualitative | The three-year pre-medical programme ‘Be- coming a Physician’ focuses on different aspects of medical professionalism. Objectives are to increase awareness and sensitivity to disadvantaged populations, and practise sensitive effective communication skills. | The curriculum includes: (1) Visits to treatment centres for people with special needs, mental illnesses, sub- stance abuse issues, physically or sexually abused, and prisoners. Students tour the facility, hold discussions with resi- dents, and discuss ethical professional interrelations to the medical world. Students then write ‘reflective diaries’ summarizing their thoughts and emotions. (2) Participation in a communication course that focuses on learning by practising patient-oriented communication. Qualitative data were collected from three sources: reflective diaries, students’ course evaluations, and interviews with the students’ tutors. | Data indicated that the students were very satisfied with the programme. They indicated an increase in aware- ness of the special needs of diverse populations, and in the sense of efficacy for conducting interviews tailored to patients' needs. Tutors reported a sense of ‘personal growth’ following their role as mentors. | Interactions of medical students with diverse populations, when accompanied by appropriate feedback mechanisms and strengthening of communication skills, can improve awareness and sensitivity to patients' special needs. This could help students become more sensitive and thoughtful physicians. | NA | 8 |
| Seddon, Kate  Anderson, Liz | Benefits of reflection: A study into the value of small group tutorials to promote reflection in Psychiatry students | Qualitative | A reflective style of learning can increase the level of student engagement with learning that encompasses complex relationships between clinical facts, clinical practice and professional identity (Goldie 2008). However, learning environments that stimulate reflection can be limited by the context in which teaching and learning take place. One factor that may contribute to this is the experience of the medical educator who might be less used to attending to learners’ affective domains (Weurlander and Stenfors-Hayes 2008). This may explain the underutilisation of reflective styles of teaching. In Bristol the delegation of clinical teaching to seven Academies has enabled small groups of students to meet the same clinical tutor weekly. We sought to evaluate the potential of this arrangement to allow the Psychiatry tutors in a local Trust (AWP) to promote a reflective learning environment. | Non-participatory observational methods were used in an ethnographic design (Denzin and Lincoln 2005). The sample was purposive: six tutors in a local Mental Health Trust where both researchers worked and who were familiar with the students and tutors. The literature informed the development of an observation guide, which eventually provided the basis for our coding frame. Observation of one tutorial session (~60 minutes) per tutor was carried out. Data were detailed field notes to which the coding frame was applied (Figure 1). Following primary coding and collation of the data from all field notes the researchers met and through an iterative process developed the broad categories into more robust themes. | Three interrelated themes were identified. A reflective space is a learning environment (the physical and emotional space) that promotes student self-awareness (Clandinin and Cave 2008). In our sample we found that it was collaboratively created by the tutors and students. However, the tutor appears to have far greater influence on the quality and quantity of the reflective space. The tutor could adopt many roles (see Table 1) associated with different teaching approaches. Constructivist strategies created more reflective space for the students to occupy, while providing knowledge or information about exams, for example, was less successful and unconsciously ‘blocked’ opportunities for reflection to occur. Students were engaged in the tutorial at different levels but if they were ‘given’ space to reflect then they did so and at high levels. | The potential to create reflective space requires explicit awareness of its potential. Tutors may wish to have further support in developing the necessary (and complex) teaching skills to extend their practice (Roebuck 2007). More work is required to elicit tutors’ accounts of their teaching role to complement our observations. However, our findings suggest that in order to promote reflection and critical thinking in our students, medical educators need to use teaching strategies that encourage students to share their experiences (openly) and then build on this to develop deeper understanding. | NA | 8 |
| Olive, K. E.  Abercrombie, C. L. | Developing a Physician׳s Professional Identity Through Medical Education | Not primary study | This report advocates for expansion of educational objectives to include a consideration of the evolution of professional identity [3,8,13]. It has been proposed that educational interventions can be guided to support and assist learners in PIF, and this reframing will shift the interpretation of professionalism from one of “doing the work of a physician” toward a broader focus of “being a physician” [3,13,14]. | Nil | Observations of resident and attending physicians have shown that sentinel events in medicine often trigger personal growth [24,25]. There have been recommendations to focus on crises because these are crucial points where there is increased vulnerability. Reflection in such settings may result in heightened potential for growth; old values or perspectives are re- examined and construction of a new identity and deconstruction of the old must occur [13]. Using reflective narrative statements and small group discussions (online or in person), many of these impactful events can be targeted intentionally to provide opportunities for PIF | CanMEDS specifies that a physician is accountable in their role as medical expert, communicator, collaborator, manager, health advocate and scholar [7]. As a secondary competency, professionalism is a component of each of these roles. To ensure professional behavior and adequate PIF in future physicians, appropriate measures should be taken to intentionally place opportunities for reflection and normative justification in the curriculum by focusing on sentinel events. Appropriate assessment tools are necessary to demonstrate that the learner has achieved competency and the tool should be appropriate for the learner and the prescribed learning environment. Many unanswered questions remain surrounding remediation, and publication of outcomes-based interventions are needed. Once identification of learners with deficiencies has occurred, remediation is a necessary intervention. Deficiencies must be identified through assessment tools to allow development of individualized learning plans, and deliberate practice, feedback, and reflection must occur before a final reassessment is done to certify competency has been achieved. Faculty development is necessary to ensure a clear understanding of professionalism, the appropriate methods of teaching and assessment in various environments, and the steps shown to produce a successful remediation. | NA | NA |
| Sargeant, Joan  Mann, Karen  van der Vleuten, Cees  Metsemakers, Job | "Directed" Self-Assessment: Practice and Feedback within a Social Context | Review article | Accurate self-assessment appears to be difficult and, some would propose, even impossible. Re- cent reviews suggest that peer assessment may be more accurate and that multisource feedback (MSF) may inform self-assessment. We had conducted a series of studies of family physicians in an MSF program including assessments from patients, medical colleagues, and coworkers and self-assessment. Using this body of research, this article explores self-assessment within the social context of multisource feedback and investigates the influ- ence of feedback from peers and others upon self-assessment. | This is a review article in which we synthesized findings of the series of studies with respect to self- assessment, used conclusions to propose a model for self-assessment within a social context, and suggest prac- tical and research implications. | Physicians compared peers’ and others’ assessment feedback with global self-perceptions of perfor- mance. Negative feedback, especially from medical colleagues, that was inconsistent with self-perceptions was not readily reconciled with self-assessments. Multiple internal and environmental factors influenced reconciliation and assimilation of negative feedback. Reflection upon feedback and self-perceptions appeared to be instrumental to reconciliation, and reflection could be facilitated. | We propose a model of “directed” self-assessment to facilitate the integration of external feedback, especially negative feedback, with self-perceptions and enable its use for practice improvement. Implications for education and research include increasing understanding of ways physicians assimilate external feedback and of the role of educators as facilitators of “directed” self-assessment and self-learning to assist physicians in integrat- ing external feedback. | *NA* | *NA* |
| van Braak, M.  de Groot, E.  Veen, M.  Welink, L.  Giroldi, E. | Eliciting tacit knowledge: The potential of a reflective approach to video-stimulated interviewing | Review article | ‘How to know what others know?’ is a pertinent question debated extensively in education [1]. The *tacit knowledge of professionals*, the seldom expressed knowledge that guides professional practice [2, 3], has received particularly ample attention [4] for good reason. Explicating tacit knowledge can aid professionalization in several ways: it can improve performance by encouraging professionals to reflect on their behaviour; it can help communicate knowledge to others; it can facilitate evaluation by linking aspects of behaviour to outcomes of that behaviour; and finally it can aid construction of ‘artefacts’ that can assist in daily practice. | Nil | In both studies, eliciting tacit knowledge was not men- tioned as the primary aim. We now show two of our own studies as examples of how medical education research us- ing reflective VSI can uncover tacit knowledge. In one of our current research projects we use video-recordings of educational sessions for general practitioners in training to stimulate teachers of the recorded sessions to reflect on the tacit knowledge that guides their behaviour when they teach these sessions. These interviewee contributions can be seen as descriptions of professional craft knowledge [18]. When shared with teachers, these descriptions can facilitate teacher professionalization [5]. In another study, we asked clinician pharmacists to reflect on boundary-crossing con- versations with their supervising general practitioner [28]. In both studies, we were interested in participants’ *actual reflection on particular professional behaviour.* The reflective VSI functioned accordingly, stimulating reflective discussion on the issues of interest. | Reflective VSI, unlike traditional applications of VSI as a tool to stimulate recall, can benefit medical education researchers who aim to uncover participants’ implicit theories, reflections on key events, or seemingly mundane, possibly unconscious, routines in a real situation of interest. By eliciting this tacit knowledge, reflective VSI captures the knowledge and expertise of educators, medical professionals, medical students, residents, and patients for later access by, for example, inexperienced professionals. Reflective VSI can also serve a powerful practical purpose, as it can help participants develop insights into their own participation, interaction, and behaviour in the settings in- volved, ultimately leading to improved medical or educational conduct.  Researchers applying reflective VSI, however, will encounter several hurdles along the way. For a good ap- plication of the reflective VSI tool, researchers should make explicit their own non-neutrality, design apt inter- view prompts, and analyze the interview as collaborative meaning construction. Each of these measures strengthens reflection on past experience. These reflections can help us find answers to questions about the tacit knowledge possessed by others, thus making reflective VSI a powerful tool in the continuing debate on ‘how to know what others know’. | NA | NA |
| Nothnagle, M.  Reis, S.  Goldman, R. E.  Anandarajah, G. | Fostering professional formation in residency: development and evaluation of the "forum" seminar series |  | The authors created a seminar series, the “Forum,” to support resident professional formation and address the hidden curriculum as part of a larger intervention to support self-directed learning skills such as goal setting and re- flection. | Ninety-minute sessions with senior residents and faculty held every other month include opportunities for individual reflection, small- and large-group discussion, and brief didac- tic components focused on skills such as teaching and leadership. The qualitative program evaluation included analyses of individual semistructured interviews with resident and faculty participants from 2008 to 2011 and of notes recorded by an observer during the 1st year’s sessions. | Residents appreciated the focus on relevant issues, presence of faculty, opportunities for reflection and interactivity, and inclusion of practical skills. Effects attributed to the Forum included gaining practical skills, feeling a deeper connection to one another and a sense of community, and recognizing progress in their own professional development and growth. Elements described in the literature as essential to professional formation, including encouraging reflection, use of narrative, role modeling, addressing the hidden curriculum, and fostering an au- thentic community, were recognized by participants as integral to the Forum’s success | A group forum for reflection and discussion with peers and role models, tailored to local needs, offers an effective structure to foster professional formation in residency. | **NA** | **13** |
| Schulz, C.  Wenzel-Meyburg, U.  Karger, A.  Scherg, A.  In der Schmitten, J.  Trapp, T.  Paling, A.  Bakus, S.  Schatte, G.  Rudolf, E.  Decking, U.  Ritz-Timme, S.  Grünewald, M.  Schmitz, A. | Implementation of palliative care as a mandatory cross-disciplinary subject (QB13) at the Medical Faculty of the Heinrich-Heine-University Düsseldorf, Germany | Quantitative | By means of the revision of the Medical Licensure Act for Physicians (ÄAppO) in 2009, undergraduate palliative care education  (UPCE) was incorporated as a mandatory cross sectional examination subject (QB13) in medical education in Germany. Its implementation still constitutes a major challenge for German medical faculties. There is a discrepancy between limited university resources and limited patient availabilities and high numbers of medical students. Apart from teaching theoretical knowledge and skills, palliative care education is faced with the particular challenge of imparting a professional and adequate attitude towards incurably ill and dying patients and their relatives. | Against this background, an evidence-based longitudinal UPCE curriculum was systematically developed following Kern’s Cycle [1] and partly implemented and evaluated by the students participating in the pilot project. Innovative teaching methods (virtual standardised/simulated patient contacts, e-learning courses, interdisciplinary and interprofessional collaborative teaching, and group sessions for reflective self-development) aim at teaching palliative care- related core competencies within the clinical context and on an interdisciplinary and interprofessional basis. | After almost five years of development and evaluation, the UPCE curriculum comprises 60 teaching units and is being fully implemented and taught for the first time in the winter semester 2014/15. The previous pilot phases were successfully concluded. To date, the pilot phases (n=26), the subproject “E-learning in palliative care” (n=518) and the blended-learning elective course “Communication with dying patients” (n=12) have been successfully evaluated. | All conducted development steps and all developed pro- grammes are available for other palliative care educators (Open Access). The integrated teaching formats and methods (video, e-learning module, interprofessional education, group sessions for reflexive self-development) and their evaluations are intended to make a contribution to an evidence-based development of palliative care curricula in Germany. | 10.5 | NA |
| Pinto-Powell, R.  Lahey, T. | Just a Game: the Dangers of Quantifying Medical Student Professionalism | Review article | A medical student on her internal medicine clerkship says her numerical medical professionalism grade was Bjust a game.^ Building on this anecdote, we suggest there is good reason to believe that numerical summative assessments of medical student professionalism can, paradoxically, undermine medical student professionalism by sap- ping internal motivation and converting conversations about core professional values into just another hurdle to residency. We suggest better ways of supporting medical student professional development, including a portfolio comprised of written personal reflection and periodic 360° formative assessment in the context of longitudinal faculty coaching. |  | We should avoid, steadfastly, the temptation to debase these nuanced, humble, evolving assessments by converting them into oversimplified numerical summative scores that emphasize hierarchy and gaming the residency application system over true reflection about the core values of medicine. | he time is long past to find better and more nuanced ways to assess professionalism in students than the current numerical system pervasive in medical education to- day. We must model the behaviors and attitudes we expect in our students and be willing to listen to the feedback we are receiving. Only then can we move forward, designing evaluative measures that will create and support the future we envision. | NA | NA |
| Svantesson, M.  Löfmark, R.  Thorsén, H.  Kallenberg, K.  Ahlström, G. | Learning a way through ethical problems: Swedish nurses' and doctors' experiences from one model of ethics rounds | Qualitative | To evaluate one ethics rounds model by describing nurses’ and doctors’ experiences of the rounds | Philosopher-ethicist-led interprofessional team ethics rounds concerning dialysis patient care problems were applied at three Swedish hospitals. The philosophers were instructed to promote mutual understanding and stimulate ethical reflection, without giving any recommendations or solutions. Interviews with seven doctors and 11 nurses were conducted regarding their experiences from the rounds, which were then analysed using content analysis. | The goal of the rounds was partly fulfilled. Participants described both positive and negative experiences. Good rounds included stimulation to broadened thinking, a sense of connecting, strengthened confidence to act, insight into moral responsibility and emotional relief. Negative experiences were associated with a sense of unconcern and alienation, as well as frustration with the lack of solutions and a sense of resignation that change is not possible. The findings suggest that the ethics rounds above all met the need of a forum for crossing over professional boundaries. The philosophers seemed to play an important role in structuring and stimulating reasoned arguments. The nurses’ expectation that solutions to the ethical problems would be sought despite explicit instructions to the contrary was conspicuous. | When assisting healthcare professionals to learn a way through ethical problems in patient care, a balance should be found between ethical analyses, conflict resolution and problem solving. A model based on the findings is presented. | NA | 10 |
| Seymour, P.  Watt, M.  MacKenzie, M.  Gallea, M. | Professional Competencies ToolKit: Using Flash Cards to Teach Reflective Practice to Medical Students in Clinical Clerkship | Qualitative | Early clinical experiences can be overwhelming to medical students. The Professional Competencies ToolKit (ProComp ToolKit) gives medical students a framework on which to build these early experiences and reflect on issues related to professionalism as each patient encounter unfolds. | The ProComp ToolKit is a set of 28 flash cards, grouped within six domains of professional competency. Each flash card is a tool for learning, defining a topic in a catchy title and laying out a specific set of skills to be acquired within the clinical setting. Tasks and tips on each flash card guide students through the process of clinical observation, self-observation, and patient interactions. Students meet in small groups with facilitators every 2 weeks throughout the year to discuss these experiences. At the end of the year, students write a narrative based on the flash card that was most meaningful to their professional identity development | We demonstrated how the student narratives that emerged from using the flash cards, exchanged in a small-group setting, led to group problem solving and validation of students’ experiences and values. In the narratives, students discussed the origin of negative behaviors and attitudes that can become normalized in patient care while asserting the primacy of patient- centered care and devising self-awareness strategies | Our experience using the ProComp ToolKit shows that teaching reflective practice can successfully be integrated into students’ clinical experiences. Professionalism skills can be reflected upon such that they become habitual and integral to students’ developing professional identities. | NA | 7 |
| Roberts, C.  Stark, P. | Readiness for self-directed change in professional behaviours: Factorial validation of the Self-reflection and Insight Scale | Quantitative | Self-reflection, the practice of inspecting and evaluating one’s own thoughts, feelings and behaviour, and insight, the ability to understand one’s own thoughts, feelings and behaviour, are central to the self-regulation of behaviours. The Self-Reflection and Insight Scale (SRIS) measures three factors in the self-regulation cycle: need for reflection; engagement in reflection, and insight | We used structural equation modelling to undertake a confirmatory factor analysis of the SRIS. We re-specified our model to analyse all of the data to explain relationships between the SRIS, medical student characteristics, and responses to issues of teaching and learning in professionalism. | The factorial validity of a modified SRIS showed all items loading significantly on their expected factors, with a good fit to the data. Each subscale had good internal reliability (> 0.8). There was a strong relationship between the need for reflection and engagement in reflection (r = 0.77). Insight was related to need for reflection (0.22) and age (0.21), but not to the process of engaging in reflection (0.06). | Validation of the SRIS provides researchers with a new instrument with which to measure and investigate the processes of self-reflection and insight in the context of students’ self-regulation of their professionalism. Insight is related to the motive or need for reflection, but the process of reflection does not lead to insight. Attending to feelings is an important and integral aspect of self-reflection and insight. Effective strategies are needed to develop stu- dents’ insight as they reflect on their professionalism. | 9 | NA |
| Raut, A. V.  Gupta, S. S. | Reflection and peer feedback for augmenting emotional intelligence among undergraduate students: A quasi-experimental study from a rural medical college in central India | Quantitative | Professionalism is the foundation of the doctor–patient relationship with emotional competency at its core. This competency is based on emotional intelligence (EI), contributing to effective work performance. As EI is reasonably new in health professions education in India, this study was conducted to assess the effect of reflection and peer feedback on EI scores among undergraduate medical students and explore facilitating and hindering factors for augmenting EI | This pre‐ and post interventional study was conducted among 94 final‐year (Part I) medical students from a rural medical college in Central India. Baseline assessment of EI scores was done using a self‐reported validated EI scale, and then, a mini‐workshop was conducted to sensitize students on EI and to train them in writing reflection and giving peer feedback. Between the pretest and posttest, students were asked to write weekly reflection and take monthly feedback from the “peer” of their choice. Posttest assessment was done at 1 week, 1 month, and 3 months. Force‐field analysis was undertaken with 10 students each with highest and lowest EI scores to assess facilitating and hindering factors. | There was a significant improvement (P < 0.0001) in the EI scores at each subsequent time point from baseline. Students who were male, from nuclear families and considered themselves spiritual had significantly higher median EI scores. Students reported self‐motivation, social support, and openness to learn new things as enabling forces for augmenting EI. Hindering factors were time constraint and lack of mutual trust in the relationship between students. | Based on the findings, it can be concluded that personal introspection methods such as self‐reflection and peer feedback help to improve the EI of undergraduate students. Therefore, it is imperative that the students are trained in these skills for building their emotional competencies. | 12.5 | NA |
| Pololi, L.  Clay, M. C.  Lipkin, M., Jr.  Hewson, M.  Kaplan, C.  Frankel, R. M. | Reflections on integrating theories of adult education into a medical school faculty development course | Qualitative and quantitative | he purpose of this study was to test a three-day course model for medical school faculty designed to promote self- directed learning, teaching skills, personal awareness and inter- disciplinary collegiality. | The training program described was conducted three times in our medical school. Fifty-eight faculty from 11 clinical departments have participated in this intensive experience of learning how to teach, based on principles of learner-centered learning and adult education theory. Participants defined their own learning objectives and worked collaboratively in facilitated small groups to develop teaching skills. Reflection groups engaged in discussion on critical inci- dents of experience as teachers and learners, and promoted awareness regarding personal approaches to teaching. | Qualitative and quantitative data showed that the course was effective in: (1) providing an academically and emotionally safe environment for learning; (2) enabling participants to recognize and value learner-centered learning; (3) increasing participant personal awareness, and (4) promoting interdisciplinary collegiality. End-of-course data assessing the following course attributes, using a five-point scale, where 1 was ‘not effective’ and 5 was ‘very effective’, showed: (a) exploration of needs: mean 4.20 ± SD 0.91; (b) interactive sharing of ideas; mean 4.60 ± SD 0.58; (c) opportunity to receive feedback: mean 4.26 ± SD 0.80; (d) opportunity to practice new skills; mean 4.11 ± SD 0.72. In terms of participation in further faculty development, 92% of participants committed themselves to continue the work begun at the course. | It was concluded that the faculty development program created a safe, learner-centered environ- ment for participants that promoted both awareness of and commitment to self-directed learning, and facilitated teaching skill development and interdisciplinary collegiality. Our three- day course appears to be highly effective in initiating a long-term faculty development process. Additionally, we conclude that there is a need for longitudinal follow-up to support and expand mastery of these teaching skills. | 10.5 | 11 |
| Frankford, D. M.  Patterson, M. A.  Konrad, T. R. | Transforming practice organizations to foster lifelong learning and commitment to medical professionalism | Opinion piece | The authors maintain that if these activities are properly structured within practice organizations, they can become powerful engines of socialization to enhance physicians’ lifelong learning and commitment to medical professionalism. They propose that this promise can be realized if new or reformed practice organizations combine education and service delivery and institutionalize processes of individual and collective reflection. | Nil | Nil | Overall, this institutionalization of reflective practice would enrich practice with education and education with practice, and accomplish the ideals of what the authors call ‘‘responsive medical professionalism.’’ The medical profession would both con- tribute and be responsive to social values, and medical work would be valued intrinsically and as central to practitioners’ self-identity and as a contribution to the public good. | NA | NA |
| Jarvis-Selinger, S.  MacNeil, K. A.  Costello, G. R. L.  Lee, K.  Holmes, C. L. | Understanding Professional Identity Formation in Early Clerkship: A Novel Framework | Qualitative | Medical educators should foster students’ professional attitudes because individuals are more likely to act in accordance with medicine’s professional values if these values have been internalized. Still,  there is much to be learned about how students examine and negotiate their emerging identities. This study examined third-year medical students’ experiences of professional identity formation (PIF) during clinical clerkship. | The authors relied on an interpretivist perspective, informed by a grounded theory approach, to analyze data, which were collected from a pilot course designed to support medical students’ efforts to “unhide” the hidden curriculum in relation to their development as medical students and emerging professionals. | Twelve third-year medical students engaged in 10 collaborative discussions with 3 faculty members, a resident, and a fourth-year student (2015–2016). Discussions facilitated students’ reflection on their professional journeys. Analysis of transcribed discussions resulted in  a conceptual framework useful for exploring and understanding students’ reflections on their PIF. Through analyzing students’ experiences, the authors identified 4 components that constituted PIF stories: context, focus, catalyst, process. | The analysis resulted in the development of a conceptual framework and distinct identity formation themes. Discrete reflections focused on either students’ current identity (being) or their sense  of future self (becoming). The study identified catalysts that sparked participants’ introspection about, or their processing of, identity. The moments that generate profound feelings of awareness in students are often moments that would not be recognizable (even post hoc) as remarkable by others. | NA | 13 |
| Sandars, J. | The use of reflection in medical education: AMEE Guide No. 44 | Opinion article | Reflection is a metacognitive process that creates a greater understanding of both the self and the situation so that future actions can be informed by this understanding. Self-regulated and lifelong learning have reflection as an essential aspect, and it is also required to develop both a therapeutic relationship and professional expertise. There are a variety of educational approaches in undergraduate, postgraduate and continuing medical education that can be used to facilitate reflection, from text based reflective journals and critical incident reports to the creative use of digital media and storytelling. The choice of approach varies with the intended outcomes, but it should also be determined by the user since everyone has a preferred style. Guided reflection, with supportive challenge from a mentor or facilitator, is important so that underlying assumptions can be challenged and new perspectives considered. Feedback also has an important role to enhance reflection. There is little research evidence to suggest that reflection improves quality of care but the process of care can be enhanced. | Nil | Reflection is a metacognitive process that creates greater understanding of self and situations to inform future action.  . Reflection has a variety of intended outcomes. Self- regulated and lifelong learning have reflection as an essential aspect, and it is also required to develop both a therapeutic relationship and professional expertise.  . There are a variety of educational approaches in undergraduate, postgraduate and continuing medical education that can be used to facilitate reflection but these should be determined by the user.  . Guided reflection and feedback are important for effective reflection.  . Although there is no evidence to suggest that reflection actually does improve patient care it seems logical and likely since the process of care can be influenced. | Reflection is an essential component of medical education and it has a variety of intended outcomes and approaches. Important aspects of reflection include its use before, during and after experiences. Reflection can be developed by individuals but guided reflection with a supervisor or mentor is important so that underlying beliefs and assumptions can be challenged within a supportive relationship. The approach to reflection should be determined by the individual since there are different preferred approaches, especially in medical students. Although there is no evidence to suggest that reflection actually does improve patient care it seems logical and likely since the process of care can be influenced. | NA | NA |
| Wittich, C. M.  Pawlina, W.  Drake, R. L.  Szostek, J. H.  Reed, D. A.  Lachman, N.  McBride, J. M.  Mandrekar, J. N.  Beckman, T. J. | Validation of a method for measuring medical students' critical reflections on professionalism in gross anatomy | Quantitative | The aims of this prospective validation study at the Mayo Medical School and Cleveland Clinic Lerner College of Medicine were: (1) to develop and validate a new instrument for measuring reflection on professionalism, and (2) determine whether learner variables are associated with reflection on the gross anatomy experience. | n instrument for assessing reflections on gross anatomy, which was comprised of 12 items structured on five-point scales, was developed. Factor analysis revealed a three-dimensional model including low reflection (four items), moderate reflection (five items), and high reflection (three items). Item mean scores ranged from 3.05 to 4.50. | The overall mean for all 12 items was 3.91 (SD 5 0.52). Internal consistency reliability (Cronbach’s a) was satisfactory for individual factors and overall (Factor 1 a 5 0.78; Factor 2 a 5 0.69; Factor 3 a 5 0.70; Over- all a 5 0.75). Simple linear regression analysis indicated that reflection scores were negatively associated with teamwork peer scores (P 5 0.018). | The authors report the first validated measurement of medical student reflection on professionalism in gross anatomy. Critical reflection is a recognized component of professionalism and may be important for behavior change. This instrument may be used in future research on professionalism among medical students. | 12.5 | NA |

| **Authors/Year** | **Article Title** | **Type of Study** | **Study Aim** | **Methodology** | **Key Findings** | **Conclusions** | **MERSQI** | **COREQ** |
| --- | --- | --- | --- | --- | --- | --- | --- | --- |
| Cavazos Montemayorr Et Al. 2020 | Understanding the dimensions of a strong professional identity: a study of faculty developers in medical education | Qualitative | The objective of this study was to understand how the professional identity of faculty developers is formed. | A qualitative approach was used, with a semi-structured interview. The sample consisted of 10 medical educators. A deductive thematic analysis based on Bolivar et al. (2004a) model of professional identity formation for medical educators was carried out. | Self-image was impacted favorably through social recognition from students and peers, and the belief of having demonstrated professional competence through job assignments and enrollment in different leadership positions. The social relations to the center or department in which the faculty developer participates were strongly related to job satisfaction. Expectations about the future of the profession included positive attitudes toward change brought by generational differences. Regarding the process of construction of professional identity, life stories and dissimilar professional careers converge in the same educational setting. Faculty developers regularly resort to self-reflection, with a desire to continue learning and developing. They are resilient and purposeful, even in negative experiences that they have faced as identity crises. They share an awareness in building a legacy for the patients, their families, and the community through nurturing new generations of health-care practitioners. | The interviewed faculty developers have a strong-professional identity that is characterized by a stable sense of self, strong behavioral repertoire, and key associations with a community of practice. | NA | 20 |
| CLARE DELANY and HEATHER GAUNT, 2018 | “I Left the Museum Somewhat Changed” Visual Arts and Health Ethics Education | Commentary article | This article aims to contribute to discussions about ethics education pedagogy and teaching, by presenting and evaluating the use of the visual arts as an educational approach designed to facilitate students’ moral imagination and independent critical thinking about ethics in clinical practice. | We describe a sequence of ethics education strategies over a 3 year Doctor of Physiotherapy program, focusing on the final year professional ethics assessment task, which involved the use of visual arts to stimulate the exploration of ethics in healthcare. The data (in the form of student essays about their chosen artwork) were analyzed using both thematic and content analysis. | Two key themes centered on emotional responses and lateral thinking. The use of artwork appeared to facilitate imaginative, emotional, and conceptual thinking about ethics and clinical experience (both past and future). This study provides some evidence to support the effectiveness of the use of the visual arts in promoting students’ recognition of ethical dimensions within their clinical experience and reflection on their emerging professional identity. | Based on our analysis of students’ essays, integrating visual art into an ethics education framework did seem to assist in fostering access to feelings, not just intellectual understanding of ethical principles, and to facilitate students’ engagement with a broader literature to frame and make sense of the ethical dimensions of their clinical practice. Students’ analysis and commentary about the experience suggested that the opportunity to use images and art as a prompt enabled them to bring independent thought and choice, and from our perspective, they incorporated their own emerging professional identity into the ethical analysis and reflection. | NA | NA |
| Kung Et Al, 2015 | Reflective Practice:  Assessing Its Effectiveness to Teach Professionalism in a Radiology Residency | Quantitative | Professionalism can be challenging to teach during residency training. We undertook this study to evaluate the impact of a case-based, reflective practice (RP) curriculum on the attitudes of radiology residents about professionalism. | We developed a case-based radiology-specific RP curriculum focused on topics related to professionalism and ethics. This year-long curriculum was comprised of six individual sessions and was attended by radiology residents. We assessed the program using the Penn State College of Medicine Professionalism Questionnaire, a validated instrument designed to assess attitudes toward professionalism, with anonymous responses collected before and after completion of the 1-year curriculum. We also obtained feedback on individual sessions. | Our curriculum affected the professional attitudes of residents in 7 of 36 sample items on the professionalism questionnaire (P <0.05), when analyzed as unpaired data. When stratified into seven specific elements of professionalism, significant differences in resident response were identified in the areas of accountability, honor and integrity, enrichment, and duty. Furthermore, residents generally agreed that the individual sessions were meaningful and were important to their future careers (3.8–4.4 on a five-point scale). | A case-based, RP curriculum centered about professionalism offers a unique practical approach to expose residents to the concepts of professionalism and ethics in a small group setting. Based on a widely used validated survey instrument, our results indicate that this method raises resident awareness about professionalism and impacts the way in which residents think about this topic and their eventual career | 9 | NA |
| Benjamin H. Levi & Michael J. Green, 2009 | Humanities In Full Retreat | Mixed | Professionalism is a crucial component that sustains the status and practice of clinical medicine. | At the Penn State College of Medicine, Milton S. Hershey Medical Center, the Department of Humanities has developed a cross-disciplinary, annual retreat for resident leaders to help them reflect on the nature of professionalism and on what it means to be a role model. The retreat also provides resident leaders the opportunity to discuss common challenges such as dealing with the stress of residency, giving bad news, working with “difficult” patients, dealing with one’s mistakes, and finding meaning and purpose in medicine. | The retreat is evaluated by resident participants using a Likert scale and open-ended responses and has received consistently excellent evaluations. | We have developed an effective, consistently successful retreat for providing residents across the spectrum of medical training with an enriching humanities experience. Such a retreat could be easily exported to other institutions to promote professionalism during residency. | 7 | 6 |
| Torgeir Gilje Lid, Rune Eraker, Kirsti Malterud, 2004 | “I recognise myself in that situation . . .” Using photographs to encourage reflection in general practitioners | Qualitative | We developed a  photography based strategy to facilitate and stimulate reflections on clinical practice and on what it means to be a general practitioner. | We chose four general practitioners as models to cover both sexes and different nationalities, ages, and practice location. The photographer spent five to eight days with each doctor, capturing encounters with patients in the practice, on call, in nursing homes, and at a child health centre.  The reflective strategy was developed on the basis of experiences and discussions in three groups.5 | The participants stated that the photographs had stimulated many thoughts and memories. Some said that the images had also encouraged them to reflect on situations never experienced.  Based on this reflective strategy we have produced  a package of16 of the photographs. To avoid the need to have technical equipment to view the photographs, we opted for prints (20 cm×30 cm). Three copies of each image were produced to accommodate being used in groups of 6-12 participants. The package has now been made available by the Norwegian Medical Association for the tutors of specialisation groups in general practice. | Narrative based reflection in groups is a powerful  method for teaching empathy. In our group meetings everyone was encouraged to give their views on several photographs. The photographs themselves did not tell a story, but provided a prompt for new stories. Photographs may capture a moment in time and are open to interpretation, but everyone’s story is equally valid. | NA | 12 |
| Farnan Et Al, 2007 | Promoting Professionalism via a Video-Based Educational Workshop for Academic Hospitalists and Housestaff | Quantitative | Unprofessional behavior can compromise care and detract from the hospital learning environment. Discrepancy between professional behaviors formally taught and what is witnessed has become increasingly evident | With funding from the American Board of Internal Medicine Foundation, a workshop was developed to address unprofessional behaviors related to inpatient care previously identified in a multi-institution survey. The aims were to utilize video-based education to illustrate unprofessional behaviors, how faculty play a role in promoting such behaviors, and facilitate reflection regarding motivation for and prevention of these behaviors. Hospitalists and housestaff at 3 Chicago-area academic hospitals and 1 community teaching affiliate participated. Videos were debriefed, identifying barriers to professional behavior and improvement strategies. A postworkshop survey assessed beliefs on behaviors and intent to change practice. | Forty-four (53%) faculty and 244 (68%) residents (postgraduate year 1 and greater) participated. The workshop was well received, with 89% reporting it “useful and effective.” Two-thirds expressed intent to change behavior. Most (86%) believed videos were realistic and effective. Those who perceived videos as “very realistic” were more likely to report intent to change behavior (93% vs 53%, P50.01). | Video-based education is a feasible way to promote reflection and address unprofessional behaviors among providers and may positively impact the learning environment. | 18 | NA |
| O’Sullivan Et Al, 2021 | Exploring attributes of high-quality clinical supervision in general practice through interviews with peer-recognised GP supervisors | Qualitative | Clinical supervision in general practice is critical for enabling registrars (GP trainees) to provide safe medical care, develop skills and enjoy primary care careers. However, this largely depends on the quality of supervision provided. There has been limited research describing what encompasses quality within GP clinical supervision, making it difficult to promote best practice. This study aimed to explore the attributes of high-quality clinical supervision for GP registrars. | In 2019–20, 22 semi-structured interviews were conducted with GP supervisors who were peer nominated as best practice supervisors, by Regional GP Training Organisations and GP Colleges in Australia. Purposeful sampling sought respondents with diverse characteristics including gender and career stage, practice size, state/territory and rurality. Interviews were conducted by video-consultation and recorded. De-identified transcripts were independently coded using iterative, inductive thematic analyses to derive themes that reflected quality in GP supervision | Seven themes emerged. Participants understood the meaning of quality supervision based on their experience of being supervised when they were a registrar, and from reflecting and learning from other supervisors and their own supervision experiences. Quality was reflected by actively structuring GP placements to optimise all possible learning opportunities, building a secure and caring relationship with registrars as the basis for handling challenging situations such as registrar mistakes. Quality also encompassed sustaining and enhancing registrar learning by drawing on the input of the whole practice team who had different skills and supervision approaches. Strong learner-centred approaches were used, where supervisors adjusted support and intervention in real-time, as registrar competence emerged in different areas. Quality also involved building the registrar’s professional identity and capabilities for safe and independent decision-making and encouraging registrars to reflect on situations before giving quality feedback, to drive learning. | This study, although exploratory, provides a starting point for understanding the quality of supervision in general practice from the perspective of GP supervisors who are peer recognised for their supervision work. | NA | 15 |
| Kayhan Parsi, JD, and Justin List, 2008 | Preparing Medical Students for the World: Service Learning and Global Health Justice | Commentary | In this article, we discuss the growth of international service learning in undergraduate medical education and tie it to a burgeoning interest among students and educators in global health justice. | We pay particular attention to how service learning can affect medical students' understanding of global health justice and why justice should be a core component of service learning curricula. | The process of experience, reflection, and action is the cornerstone of cultivating a sense of social justice among students. Finally, we examine both risks and benefits to international service learning for medical students. | There are numerous other benefits to service learning opportunities for students engaged in international health. Students improve their communication and listening skills, rely less on technology and more on their clinical physical diagnosis skills, improve their knowledge of diseases prevalent in the developing world and become more sophisticated with regard to public health issues in resource-poor settings. The students develop important clinical skills for themselves as physicians in training and play an important role in partnering with clinicians in these settings. | NA | NA |
| Jennifer L. Quaintance, Louise Arnold, and George S. Thompson, 2010 | What Students Learn About Professionalism From Faculty Stories: An “Appreciative Inquiry” Approach | Qualitative | To develop a method for teaching professionalism by enabling students and faculty members to share positive examples of professionalism in a comfortable environment that reflects the authentic experiences of physicians. Medical educators struggle with the teaching of professionalism. Professionalism definitions can guide what they teach, but they must also consider how they teach it, and constructs such as explicit role modeling, situated learning, and appreciative inquiry provide appropriate models. | The project consisted of students interviewing faculty members about their experiences with professionalism and then reflecting on and writing about the teachers’ stories. In 2004, 62 students interviewed 33 faculty members, and 193 students observed the interviews. Using a project Web site, 36 students wrote 132 narratives based on the faculty’s stories, and each student offered his or her reflections on one narrative. The authors analyzed the content of the narratives and reflections via an iterative process of independent coding and discussion to resolve disagreements. | Results showed that the narratives were rich and generally positive; they illustrated a broad range of the principles contained in many definitions of professionalism: humanism, accountability, altruism, and excellence. The students’ reflections demonstrated awareness of the same major principles of professionalism that the faculty conveyed. The reflections served to spark new ideas about professionalism, reinforce the values of professionalism, deepen students’ relationships with the faculty, and heighten students’ commitment to behaving professionally. | Narrative storytelling, as a variant of appreciative inquiry, seems to be effective in deepening students’ understanding and appreciation of professionalism. | NA | 16 |
| Shmuel P. Reis, and Hedy S. Wald, 2015 | Contemplating Medicine During the Third Reich: Scaffolding Professional Identity Formation for Medical Students | Article | The moral failures of physicians and the medical establishment in Germany and Austria during the Third Reich challenge medicine and medical education in a way few other events do. They compel medical educators to ensure that lessons learned from contemplating medicine during the Third Reich be integrated into current and future physicians’ professional identities. Most health professions education programs, however, have not adopted this study domain in their curricula. | The authors describe a new curriculum module—“The Holocaust and Medicine”—and its implementation in October 2013 at Bar-Ilan University Faculty of Medicine in the Galilee, Safed, Israel, as a requirement for all medical students (starting with the class of 2017). This innovative module integrates historical facts, guided reflection, flipped classroom pedagogy, and program evaluation efforts. It spans 20 months of the preclinical curriculum, embedded within a doctoring course and a medical humanities longitudinal course and integrated within the clinical sciences blocks. | The evaluation approach will seek to measure changes in learners’ knowledge and attitudes, capture their experience with the module, and assess the module’s contribution to their identities as future healers. | This module aims to sensitize learners to medicine’s fundamental dilemmas (e.g., prejudice, assisted reproduction and suicide, physicians in war), ideally enhancing critical reflection on the potential danger of “slippery slopes.” The authors propose that contemplation of medicine after the Holocaust and the implications for contemporary practice should be an integral component of health professions education to promote humanistic, ethically responsible practice. | NA | NA |
| Shiozawa Et Al, 2019 | An Insight into Professional Identity Formation: Qualitative Analyses of Two Reflection Interventions During the Dissection Course | Mixed | The professional behavior of future doctors is increasingly important in medical education. One of the first subjects in the curriculum to address this issue is gross anatomy. The Tuebingen Medical Faculty implemented a learning portfolio and a seminar on medical professionalism during the dissection course. The aims of this research project are to get an overview of how students form a professional identity in the dissection course and to compare the content of both their oral and written reflections on the course. | A qualitative analysis was conducted of the oral and written reflections on the dissection laboratory experience. This study was conducted during winter term 2013/2014 with a cohort of 163 participants in the regular dissection course. Written reflection texts (from n = 96 students) and audio recordings from four oral reflection seminar discussions (with n = 11 students) were transcribed and deductively categorized with Mayring’s qualitative content analysis method. | Both qualitative analyses show that students reflected on many topics relevant to professional development, including empathy, respect, altruism, compassion, teamwork, and self-regulation. Quantitative analysis reveals that students who attended the oral reflection wrote significantly more in their written reflection than students who did not. There is, however, no difference in the reflection categories. Reflection content from students corresponds with categories derived from existing competency frameworks. | Both the seminar (oral reflections) and the learning portfolio (written reflections) present excellent opportunities to foster professional development during anatomy education; the key is using them in conjunction with the dissection course | 8 | 14 |
| Soo Et al, 2015 | At the precipice: a prospective exploration of medical students’ expectations of the pre-clerkship to clerkship transition | Mixed | Medical learners face many challenging transitions. | We prospectively explored students’ perceptions of their upcoming transition to clerkship and their future professional selves. In 2013, 160/165 end-of-second-year medical students wrote narrative reflections and 79/165 completed a questionnaire on their perceptions oftheir upcoming transition to clerkship. Narratives were separately analyzed by four authors and then discussed to identify a final thematic framework using parsimonious category construction. | We identified two overarching themes: (1) ‘‘Looking back’’: experiences which had helped students feel prepared for clerkship with subthemes focused on of patient care, shadowing, classroom teaching and the pre-clerkship years as foundational knowledge, (2) ‘‘Looking forward’’: anticipating the clerkship experience and the journey of becoming a physician with subthemes focused on death and dying, hierarchy, work-life balance, interactions with patients, concerns about competency and career choice. Questionnaire data revealed incongruities around expectations of minimal exposure to death and dying, little need for independent study and limited direct patient responsibility. Weconfirmed that internal transformations are happening in contemplative time even before clerkship. By prospectively exploring pre-clerkship students’ perceptions of the transition to clerkship training we identified expectations and misconceptions that could be addressed with future curricular interventions. While students are aware of and anticipating their learning needs it is not as clear that they realise how much their future learning will depend on their own inner resources. | We suggest that more attention be paid to professional identity formation and the development of the physician as a person during these critical transitions. | 6.5 | 7 |
| Stanley, 2022 | Students-As-Teachers: Fostering medical educators | Mixed | While mounting evidence supports various benefits of Students-As Teachers (SAT) curricula in preparing students to teach, limited SAT electives are offered across Canada. We developed a 4-week SAT selective for fourth-year medical students at the University of Toronto to enhance medical education knowledge and teaching skills. This study aimed to evaluate the SAT programme and its impact on students’ development as educators, their experience as learners and educators, and their future plans for involvement with medical education. | Students participated in highly interactive small group seminars and teaching opportunities in nonclinical and clinical environments. Course evaluation consisted of pre-selective and post-selective surveys and written reflections on the selective experience and future career aspirations. A theory-based evaluation approach was utilized to compare the SAT programme’s theory with course outcomes. | Post-SAT selective, students self-reported greater knowledge and confidence in teaching methods, provision of feedback, medical education scholarship, and interest in further medical education training. Student reflections highlighted three key themes. Identity formation as educators and the importance of mentorship in medical education aligned with our programme theory, while an unexpected outcome included a shifting perception on teaching and feedback from a learner to an educator lens. | This study’s findings demonstrate the ability of SAT curricula to build capacity for future medical educators. Positive factors contributing to the programme’s outcomes included cohort size, course and seminar structure, and active group participation. Future iterations may explore use of flipped classroom models, additional clinical teaching opportunities, and near-peer teaching. | 7 | 9 |
| Stocker Et Al, 2018 | Schwartz rounds in undergraduate medical education facilitates active reflection and individual identification of learning need | Qualitative | Strategies applying Schwartz Rounds to improve wellbeing of medical students has focused on the clinical years of study. | This pilot study investigates whether Schwartz Rounds could be effective in developing students’ reflective practice in Year 2 undergraduates. | Engagement with the Schwartz Round was high with over 50% of the students identifying learning needs through reflection on the Round. | Schwartz Rounds promoted recognition of the value of reflective practice and increased self-awareness of student needs. | NA | 7 |
| Teo Et Al, 2022 | Assessing professional identity formation (PIF) amongst medical students in Oncology and Palliative Medicine postings: a SEBA guided scoping review | Review Article | Introduction to a multi-professional team who are working and caring for the dying, and facing complex moral and ethical dilemmas during Oncology and Palliative Medicine postings influence a medical student’s professional identity formation (PIF). However, limited appreciation of PIF, inadequate assessments and insufficient support jeopardise this opportunity to shape how medical students think, feel and act as future physicians. To address this gap, a systematic scoping review (SSR) of PIF assessment methods is proposed. | A Systematic Evidence-based Approach (SEBA) guided SSR of assessments of PIF in medical schools published between 1st January 2000 and 31st December 2021 in PubMed, Embase, ERIC and Scopus databases was carried out. Included articles were concurrently content and thematically analysed using SEBA’s Split Approach and the themes and categories identified were combined using SEBA’s Jigsaw Perspective. The review hinged on the following questions: “what is known about the assessment of professional identity formation amongst medical students?”, “what are the theories and principles guiding the assessment of professional identity formation amongst medical students?”, “what factors influence PIF in medical students?”, “what are the tools used to assess PIF in medical students?”, and “what considerations impact the implementation of PIF assessment tools amongst medical students?”. | Two thousand four hundred thirty six abstracts were reviewed, 602 full-text articles were evaluated, and 88 articles were included. The 3 domains identified were 1) theories, 2) assessment, and 3) implementation in assessing PIF. Differing attention to the different aspects of the PIF process impairs evaluations, jeopardise timely and appropriate support of medical students and hinder effective implementation of PIF assessments. | The Krishna-Pisupati model combines current theories and concepts of PIF to provide a more holistic perspective of the PIF process. Under the aegis of this model, Palliative Care and Oncology postings are envisaged as Communities of Practice influencing self-concepts of personhood and identity and shaping how medical students see their roles and responsibilities as future physicians. These insights allow the forwarding of nine recommendations | NA | NA |
| Uygur Et Al, 2019 | A Best Evidence in Medical Education systematic review to determine the most effective teaching methods that develop reflection in medical students: BEME Guide No. 51 | Review article | Reflection is thought to be an essential skill for physicians. Although much has been written about it, there is little concurrence about how to best teach reflection in medical education. The aim of this review was to determine: (i) which educational interventions are being used to develop reflection, (ii) how is reflection being assessed, and (iii) what are the most effective interventions. | Inclusion criteria comprised: (i) undergraduate medical students, (ii) a teaching intervention to develop reflection, and (iii) assessment of the intervention. A review protocol was developed and nine databases were searched. Screening, data extraction, and analysis procedures were performed in duplicate. Due to the heterogeneity of studies, a narrative synthesis approach was performed for the study analysis. | Twenty-eight studies met the inclusion criteria. The interventions in these studies had at least of two of the following components related to reflection: (i) introduction, (ii) trigger, (iii) writing, (iv) guidelines, (v) small group discussion, (vi) tutor and (vii) feedback. Three validated rubrics were used to assess reflective writing in these studies. | The strongest evidence from studies in this review indicates that guidelines for, and feedback on, reflective writing improve student reflection. | NA | NA |
| Wen Et Al, 2014 | Exploratory study of the characteristics of feedback in the reflective dialogue group given to medical students in a clinical clerkship | Mixed | Structured narrative reflective writing combined with guided feedback is an efficient teaching method for enhancing medical students’ reflective capacity. However, what kinds of feedback offered and reflection presented in a reflective group remain unclear. The aim of this study was to investigate the characteristics of feedback in a reflective dialogue group. | Fifth-year medical students on a monthly interval rotation at the pediatric department of a medical center in eastern Taiwan during the 2012 academic year completed their reflective writing regarding patient and family psychosocial issues, and were subsequently debriefed in a 2-h group discussion session to receive feedback from a clinical tutor and peers. Content analysis was conducted to explore the characteristics of feedback and reflection presented in the reflective dialogue. The evaluative questionnaire regarding the benefits of reflection with others was administrated following the group session. | Forty students participated in five reflective groups and 108 psychosocial issues were discussed and identified. The tutor played an initiating role in the group discussion by providing six equal feedback types involving exploring new knowledge, initiating advanced discussion, highlighting the issues, and encouraging the students. The students provided eight types of feedback that involved a substantial deep discussion on psychosocial issues and action plans based on the complex interactive ecological networkofclinical encounters. Each student attained 1.25 times the depth or breadth ofreflection after receiving feedback and experienced the benefits of reflection with others. | Through structured narrative reflective writing combined with pluralistic group discussion with a tutor and peers, the medical students had time to think deeply and broadly about psychosocial issues among patients and their family members. Facilitative feedback providing new knowledge, deeper discussion, and exploring new ways of action planning for psychosocial issues was recommended to promote students’ reflective capacity. | 6 | 14 |
| Wyatt Et Al, 2020 | Linking Patient Care Ownership and Professional Identity Formation through Simulation | Qualitative | The link between ownership of patient care and professional identity formation (PIF) has not been formally established, yet PIF researchers frequently cite clinical experiences as powerful contributions to PIF. Hypothesis: Using clinical simulation, this study aimed to explore the relationship between patient care ownership and the cognitive processes involved in the creation of a professional identity. | In 2018–2019, 189 third-year students participated in a simulation in which they were placed in the role of a physician treating a patient in respiratory distress. Data were collected from 12 focus groups (n¼84; 44% of the third-year class), each lasting 15–25minute. Students were asked four questions designed to identify moments when they felt like a physician and experienced feelings of ownership. Each focus group was transcribed and analyzed for the presence of known elements that contribute to feelings of psychological ownership, and then inductively for how students related their feelings of ownership to their professional identity. | When students were asked to take ownership of their patient’s care, they underwent a three-step process: (1) experiencing disorientation, (2) reconceptualizing roles and responsibilities, and (3) reorientation to professional goals. Patient care ownership was disorienting because it marked a departure from the clinical roles the students had previously experienced. While disoriented, students engaged in a process of reflection during which they asked themselves who they were, who they were becoming, and who they needed to become to effectively serve in the role of a physician. This process prompted students to realize the limitations of their clinical reasoning abilities and that the role of a physician requires new ways of thinking. | This study advances a conceptual model of PIF that identifies patient care ownership as a catalyst in developing a professional identity. Assuming responsibility for patients places students into an unfamiliar role, which opens a channel for students to access new perspectives in their development as physicians. | NA | 18 |
| Xu Et Al, 2019 | Reflecting on exchange students’ learning: Structure, objectives and supervision | Qualitative | The increasing opportunities for medical students to participate in international electives may improve students’ professionalism and cultural competence. However, the students’ overall experiences may be unpredictable, unstructured and lack supervision. There is scant evidence with respect to their learning outcomes. These reflections demonstrate that short-term supervised elective can provide students with structured learning experiences to achieve specific learning objectives. | We carried out daily debriefs and a weekly summary with seven Curtin Medical School students from Perth, Australia during an 18-days supervised elective in the First Affiliated Hospital, Sun Yat-sen University, Guangzhou, China. The daily debriefs and the weekly summary in different disciplines become the content of the reflections discussed in this article. | The main themes identified in the feedback were as follows: Skills in history taking and physical examination; clinical reasoning; diagnosis and management of diseases rarely seen in Australia; awareness of clinical ethics; merits and demerits of different systems of healthcare; sensitivity to issues in doctor-patient relationships; work ethics; enhancement of cultural competence; and personal development. | These reflections provide insight into how overseas electives may be structured to improve students’ clinical reasoning skills in this hospital. These students achieved their learning outcomes under joint supervision from both institutions. The clinical skills learned from these experiences enhanced the students’ professionalism and cultural competence, giving students the opportunities to appreciate the multitude healthcare model of bio-psycho-social-political-economical-spiritual dimensions. | NA | 10 |
| Michael J. Green, MD, MS | Comics and Medicine: Peering Into the Process of Professional Identity Formation | Qualitative | Medical students experience transformative personal and professional changes during medical school. The medical education community has much to learn about how students perceive these changes, which can be dramatic and profound. | Over the past six years (2009–2014), the author has taught a course on medical graphic narratives (or comics) to fourth-year medical students. Comics synergistically combine words and images to tell stories and provide an effective vehicle for helping students reflect on and give voice to varied experiences. In this course, students critically read and discuss medically themed comics and create their own original comic depicting a formative experience from medical school. | To date, 58 students have taken the course, and each has produced an original comic. The author conducted a thematic analysis of their comics and identified  the following themes: (1) how I found  my niche, (2) the medical student as patient, (3) reflections on a transformative experience, (4) connecting with a patient, and (5) the triumphs and challenges of becoming a doctor. Pre/post course assessments indicate that students believe creating a comic can significantly improve a variety of doctoring skills and attitudes, including empathy, communication, clinical reasoning, writing, attention  to nonverbal cues, and awareness of physician bias. Students’ comics reveal the impact of formative events on their professional identity formation. | Medical educators should explore additional ways to effectively integrate comics into medical school curricula and develop robust tools for evaluating their short- and long-term impact. | NA | 9 |
| Dhónaill, R. N. | Physician associate student and their experiences human cadaveric dissection | Qualitative | The role of physician assistant/associate (PA) has expanded from its inception in the United States over 50 years ago, to European countries including Ireland. While there is an increasing body of evidence exploring the role and training of PAs in clinical settings, there is a scarcity of research exploring PA students’ perspectives in relation to their experience of anatomy dissection, or how these experiences may contribute to the development of their core professional identity. | Students in the first two cohorts of PA Program at the Royal College of Surgeons in Ireland program were invited to interviews which solicited them to reflect and report on their own experiences of anatomical dissection during their course. Participants’ responses were analyzed using a thematic inductive approach; common themes and patterns were organized into a hierarchical structure, which generated the final framework of themes. Ten participants took part in the study; only one had previous personal experience of dissection, while two further participants had some familiarity with prosected specimens. | The first theme concerned the participants’ expectation of anatomical dissection, with sub-themes of preconceptions, smell, and emotions. The second theme involves discussion of coping strategies that the participants used, including talking, viewing the cadaver as their first patient, and naming (or not naming) the cadaver. The third theme includes how the participants’ talked about respect and compassion in the dissection room, development of team working skills, and awareness of bereavement and organ donation. A number of recommendations were also made for the experience and orientation of future students in such a program. |  | NA | 9 |
| Byars, L. | Using art to enhance reflection on professional attributes | Qualitative | Art and humanities can enhance undergraduate medical education curricular objectives. Most commonly, art is used to help students learn observational skills, such as medical interviewing and physical diagnosis. Educators concurrently struggle to find ways to meaningfully teach professional values within crowded curricula. Aim: This curriculum aimed to combine art and reflection to actively convey tenets of medical professionalism. Setting: Internal medicine clerkship at a single institution. | Participants: Third-year students. Program description: Students reviewed an online module describing attributes of medical professionalism before completing a 4-step written exercise stimulated by viewing a work of art and based on a critical incident from their own experiences. A faculty member reviewed the essays and facilitated small group discussion to normalize the students’ emotional responses and generalize their observations to others. | The curriculum was acceptable to students and enthusiastically received by faculty. Efforts to assess the effects and durability of the exercise on student behavior are ongoing. Discussion: Artwork can enhance student reflection on professional values. | This model efficiently and creatively meets curricular professionalism objectives. | NA | 10 |
| Cohn, R. J. | A Cloud With a Silver Lining: Helping Students Learn About Professionalism | Qualitative | Educators face many challenges in teaching professionalism. Despite attempts to define professionalism, it remains abstract for students and often not fully appreciated until they are in clinic. Without a way to make it personally relevant, students will likely be less motivated to learn. Intervention: We used student-generated word clouds to facilitate reflection and discussions, thereby helping students make their perceptions of professionalism more explicit. Group discussion was followed by a 5-minute written reflection. Word clouds created in Semesters 1 and 7 enabled students to compare perceptions at two points in the curriculum. | The George Washington University Doctor of Physical Therapy Program is a 3-year, 8-semester, 109-credit postgraduate program that includes 34 weeks of clinical practice. Reflection is foundational to the curriculum, and students reflect on their learning and professional growth each semester. Historically, students were introduced to professionalism in Semester 1 using explicit instructional strategies. Despite the wealth of resources, readings, and discussions, engagement remained challenging. | Student-generated word clouds created a personally relevant visual from which uniquely designed prompts were created to facilitate discussion. Having students compare word clouds across semesters enabled them to identify what, when, how, and where they learned about professionalism. Word clouds, categorized words, and 5-minute written reflections provided evidence of individual and collective changes in student perceptions. | Students will engage in rich discussions on professionalism if it is personally relevant. Anonymity can foster discussion on personal characteristics and biases. Visualization of student-generated, narrative data enhanced reflection and discussion. Comparing word clouds from two points in time helped students articulate changes in their perceptions of professionalism. A 5-minute reflection can be a powerful learning tool for students and faculty. Outcomes demonstrated the value of designing interventions grounded in the educational principles. | NA | 7 |
