# Supplementary material for: A systematic scoping review on group non-written reflections in medical education
Source: BMC Med Educ. 2024 Oct 10;24:1119. doi: 10.1186/s12909-024-06117-3 (PMC11468106; doi:10.1186/s12909-024-06117-3)
Supplement: Supplementary file 4 — Additional file 4. Summary of Extracted Data [file 12909_2024_6117_MOESM4_ESM.docx]

**Additional File 4: Summary of Extracted Data**

| **Themes** | **Examples** |
| --- | --- |
| Purpose of using GNRW | \| General principles of use \| To counter \| Maladaptive coping mechanisms to events they were uncomfortable with   - Ethical erosion \| \| --- \| --- \| --- \| \| Hidden curriculum \| \| Difficulty in teaching professionalism which requires qualitative approach \| \| Emotional toll due to nature of healthcare   - Burnout \| \| Changing demands of the healthcare system \| \| To foster \| Reflective learning \| \| Supportive group learning culture \| \| Desirable attributes of a doctor   - Patient safety - Patient communication - Lifelong learning - Professionalism - Improve emotional capacity - Attitudes - Receiving feedback well \| \| Understanding of more complex ethical issues \| \| Medical knowledge \| \| Acceptance of the hardships along the medical journey \| \| Modality specific \| Narratives \| Provides students with structure and makes abstract concepts more relatable \| \| Small group discussions \| Higher value discussion in smaller groups \| \| Difficulty in conducting such lessons in lecture format \| \| In-depth discussion of students’ experience \| \| Reflective writing \| Allows for self-expression \| \| Enhances reflective skill that may not be intuitive \| \| Portfolios \| Assessment over time to compare with \| \| Performance assessments \| Helps identify strengths and weaknesses to enable student to understand and work on themselves better \| \| Anonymous peer feedback \| Honest assessments to help direct more accurate reflection and growth \| \| Mentoring \| Benefits both mentor and mentee \| \| Photography \| Evoking memories, feelings, conflicts that are points of discussion \| \| Anatomy class \| First contact with issue of life and death and other related topics \| \| Emotional experience \| \| Schwartz Rounds \| Show support to healthcare workers for them to maintain standard of good care \| \| Dignity interview \| Making meaning of life \| \| Holocaust lessons \| Questioning ingrained convictions \| \| Projective techniques \| Evokes creative freedom to improve insight \| \| Time-based \| Times of transition \| Typically involves more reflection \| \| Before transition \| Learning can also happen before major transitions \| |
| Structure of the GNRW process | \| Content \| Outlining topics and concepts to focus on \| \| --- \| --- \| \| Using prompts to guide reflection and discussions   - narratives - drawing - life cycle diagrams - photographs - movies - Discussions with patients/caregivers - Performance assessments - Peer feedback - Museum visit \| \| Reviewing reflections with facilitator/assessor/senior   - in group setting vs 1-1 - encouraging but not forcing students to share in the group setting \| \| Follow-up action after the intervention to promote maintenance of the learning   - watching out for certain phenomena, such as dissonance \| \| Review of faculty/programme \| \| Pre-empting students with topics to stimulate thought and facilitate better reflective process   - pre-presentation brainstorming with group \| \| Environment \| Maintaining favourable environment   - rapport - comfort \| \| Time \| Dedicated time for reflective process   - entire structured 2 day retreat - curriculum time \| \| Targeting specific time points, eg. Midway of course  regular scheduling to build rapport \| |
| Benefits of GNRW | \| Benefits to students \| Professionalism/  Desirable attributes \| Identifying experiences that hold value in teaching professional behaviour, improving professionalism \| \| --- \| --- \| --- \| \| Builds resilience \| \| empathy \| \| Skills/Knowledge \| Surgical \| \| Managing difficult emotions \| \| Medical knowledge \| \| Lifelong learning \| \| Leadership \| \| Reflection \| \| Organization and planning skills \| \| Critical thinking and problem-solving skills \| \| Moral reasoning \| \| Emotional awareness/perception \| \| Developing own practice \| \| Self-awareness \| Counters cognitive dissonance through identifying conflicting values, current state of mind, etc. \| \| Improved perception of healthcare responsibilities \| \| Improves confidence \| \| Improved understanding of environment factors \| Identifying hidden curriculum and resisting its negative impacts \| \| Complexity of the life of a doctor \| \| Experiences are common and burden is shared \| \| Feedback from patients and how to respond \| \| Social structure of the hospital \| \| Benefit to other people \| Patients \| Better understanding of the principles in patient care \| \| Reduce medical errors and improve quality of care \| \| Medical team \| Better teamwork \| \| Greater appreciation \| \| Stronger commitment \| \| Peers \| More supportive \| \| Learning from shared experiences \| \| Improved communication \| \| Benefits to institution \| Facilitates/enhances interventions \| Strengthened mentor-mentee bond \| \| Strengthens peer-peer bond \| \| Improves perception of faculty \| |
| Cons of GNRW | \| Unwanted side-effects   - Doubts born from programme negatively impacting patient care - Accepting indifference as a way to protect on self - Reinforcing stereotypes - Worries over exposing fellow participants \| \| \| --- \| --- \| \|  \| \|  \| \|  \| \|  \| \| Limited efficacy – thus a waste of resources/time \| Intervention factors   - Scenarios not fully encapsulated - Inappropriate modalities such as art, poetry that may not be suitable for all - Time constraints - No guarantee that interventions will evoke positive change \|  \| \|  \| \|  \| \|  \| \| Student factors   - Pandering to politically correct answers - Lack of follow-up by student - Influences by previous reflective experiences \|  \| \|  \| \|  \| |
| Enabling factors in implementing GNRW | - Tools for reflection   - Visual – flashcards, drawing, photographs, TV programmes/movies, comics   - Portfolio     - Proper documentation and tracking of progress     - Source material for follow-up with mentors   - Seminars and forums – reflections on discussion points serve as learning opportunities   - Patient-based – reflecting on patients’ stories improve relatability - Format of reflections   - Oral     - Deeper and more personal insights as nothing is recorded   - No fixed format     - Reflective modalities that are best suited for the circumstance can be employed     - Personal preferences that allow for creativity and flexibility     - Dynamic interactions - Group-related factors   - Size – keep to smaller groups to encourage quitter members   - Dynamics – cooperative and collaborative, familiarity with group   - Participant contributions – equal contributions by all - Facilitator-participant relationship – chemistry between facilitator and participant, building trust to facilitate reflections - Programme structure   - Adequate frequency and time allocated for reflections   - Preparation before reflection session   - Introductory and closing sessions - Reflection environment   - Suitable atmosphere – relaxed setting, good group dynamics   - Perceived environment by participants – safety, confidentiality, collaboration and support - Participant factors   - Attitude and desire to participate   - Attitude towards others in group settings   - Willingness to learn from sessions - Facilitator and faculty related factors - Facilitator skills   - Trained facilitators   - Awareness of the reflective process, giving feedback and summarizing   - Able to provide different perspectives - Facilitator commitment   - Continuity across multiple terms   - Adequate time for facilitation - Facilitator background   - Non-authoritative figure   - Non-medical   - Role model   - Mentor - Faculty   - Understanding of purpose of the reflection   - Understanding of the social aspect of the medical fraternity |
| Challenges in implementing GNRW | - Facilitators   - Facilitator training     - No formal training     - Unfamiliarity with role     - Uncomfortable attending to affective domains   - Facilitator attitude     - Negativity     - Insensitivity     - Carelessness   - Facilitator commitment     - Poor availability     - Competing clinical demands   - Varying personal approaches to coaching     - Inconsistencies confuse students     - Lack of time - Programmes   - Duration of sessions     - Inadequate time for intervention   - Timing of sessions     - During undergraduate medical curriculum       - High learning pressure and exams limit reflective capability       - High stress leading to avoidance of reflections     - During residency       - Brief encounters       - Infrequent reflection       - Lack of experience to reflect on when done too early in residency   - Structure     - Reflection process       - Clear instructions required       - Repetitive nature can be frustrating     - Authenticity of reflections       - Reduced by compulsory nature of reflections       - Poor framing of the programme affecting the programme’s importance to student     - Defining reflections       - Hard to understand reflection well       - Being guided by “common sense” versus being guided by literature     - Unclear programme goals       - Outcomes versus processes       - Poor understanding of programme goals leading to poor engagement     - Programme assessment       - Inadequate, incomplete assessments       - Uncertainty of how assessments may affect medical career   - Modalities of reflection employed     - Technology-based modalities can cause user frustration when there is inadequate technical support     - Group-based modalities may require more time for discourse for adequate reflection     - Art-based modality may be too difficult to employ due to skill limitation   - Staffing limitation     - Difficulties hiring coaches     - Lack of administrative staff support resulting in poor implementation and scheduling     - Supervisors and facilitators present may skew reflection responses   - Participation and follow-up     - Low pick-up rates     - No time to realize developmental goals identified through reflection - Participants   - Safety and confidentiality     - Possible compromises in learning environment     - Worries about broken confidentiality     - Reluctance in engaging personal stressors and problems   - Group related issues     - Difficulty opening up to bigger groups     - Feels as though there is less support     - Less emotional engagement     - Aversive attitudes   - Participants openness to receiving feedback     - Reflections skewed by what others might think     - Difficulty accepting criticism regarding own behaviour   - Competing commitments     - Exams – pushing reflections to “last minute”     - Clinical work – calls, work responsibilities   - Preconceived notions of reflections     - Perceived lack of value in reflection – waste of time, strange     - Negative attitude towards reflections   - Inexperience in using reflections     - Lack of understanding on how to reflect     - Intertwining of reflection and other cognitive concepts   - Lack of self-awareness causing misleading feelings   - Distracted discussions – side-tracking and not accomplishing goals of reflection   - Poor application to daily life     - Time constraints     - Competing work interests |
| Topics of reflection in GNRW | \| Clinical related Experience \| Rural clinical medicine \| \| --- \| --- \| \| Early clinical experience \| \| Students as teachers programme \| \| Clinical experience \| \| IM internship \| \| Psychiatry tutorial \| \| Experiences from Practice \| \| Patient’s life stories \| \| Hidden curriculum \| \| Transitional years \| Pre-clinical to clinical \| \| Good death from various perspective – respect for pts wishes, impt of open communication \| \| Non-clinical related experiences \| Relationships \| \| Study Experience \| \| International \| \| Interactions of med students with diverse populations \| \| Life experience \| \| Clinical scenario \| \| Anatomy course \| \| Historical related – background historical facts \| \| Reflection on self \| Personal and core values \| \| Peer feedback on one’s own behaviour, one’s effect on others and one’s own abilities \| \| Students behaviours and thoughts \| \| Professionalism \| Reflection on issues of profersionalism \| \| Digital professionalism \| \| Interactions with other stakeholders \| \| Clinical skillsets \| \| Professional formation \| \| Individual development \| Self-development during palliative care course \| \| Work life balance \| \| Leadership \| \| Self-directed learning \| |
| Impact on participants | \| Reflection teaches students how to behave in a professional manner \| Increases skill competency levels \| \| --- \| --- \| \| Reinforces the standards that medical students should uphold as future medical professionals \| \| Improves ability to tackle challenging clinical scenarios \| \| Reflection enables students to undergo personal growth \| Self-discovery \| \| Development of empathy \| \| Improved understanding of the medical profession \| \| Leadership development \| \| Reflection improves students’ communicative abilities \| Increased expressiveness \| \| Increased socialization \| \| Reflection was not beneficial in helping students to develop their professional identity \| Lack of participation \| \| Lack of appropriate interventions \| \| Difference in educational backgrounds \| |
| Enabling factors in assessing reflections | \| Establish standards for evaluation \| Guidelines \| \| --- \| --- \| \| Student profile \| Understanding students better \| |
| Challenges in assessing reflections | \| Inaccurate data was collected \| Subjective responses \| \| --- \| --- \| \| One-off intervention \| \| Small sample size \| \| Lack of standard for assessment \| \| Difficulties with expression \| \| Insufficient scope \| \| Technical difficulties \| Inaccessibility of portals for submission and review \| \| Administrative issues \| Insufficient allocation of resources \| \| Inappropriate intervention timing \| \| Factors associated with relevant personnel \| Lack of seriousness towards intervention \| |
| Remediation for unsatisfactory reflections | \| Students are required to make up for their “poor” reflection \| Remedial work \| \| --- \| --- \| \| Students are provided with more support \| Extra guidance \| \| Help students tackle their challenges \| \| Assess students’ stressors \| |
